# Metabolite Genome-Wide Association Study (mGWAS) and Gene-Metabolite Interaction Network Analysis Reveal Potential Biomarkers for Feed Efficiency in Pigs

**DOI:** 10.3390/metabo10050201

**Published:** 2020-05-15

**Authors:** Xiao Wang, Haja N. Kadarmideen

**Affiliations:** Quantitative Genomics, Bioinformatics and Computational Biology Group, Department of Applied Mathematics and Computer Science, Technical University of Denmark, Richard Petersens Plads, Building 324, 2800 Kongens Lyngby, Denmark; xiwa@dtu.dk

**Keywords:** feed efficiency, biomarkers, SNPs, GWAS, metabolomics, RFI, pigs, pathways

## Abstract

Metabolites represent the ultimate response of biological systems, so metabolomics is considered the link between genotypes and phenotypes. Feed efficiency is one of the most important phenotypes in sustainable pig production and is the main breeding goal trait. We utilized metabolic and genomic datasets from a total of 108 pigs from our own previously published studies that involved 59 Duroc and 49 Landrace pigs with data on feed efficiency (residual feed intake (RFI)), genotype (PorcineSNP80 BeadChip) data, and metabolomic data (45 final metabolite datasets derived from LC-MS system). Utilizing these datasets, our main aim was to identify genetic variants (single-nucleotide polymorphisms (SNPs)) that affect 45 different metabolite concentrations in plasma collected at the start and end of the performance testing of pigs categorized as high or low in their feed efficiency (based on RFI values). Genome-wide significant genetic variants could be then used as potential genetic or biomarkers in breeding programs for feed efficiency. The other objective was to reveal the biochemical mechanisms underlying genetic variation for pigs’ feed efficiency. In order to achieve these objectives, we firstly conducted a metabolite genome-wide association study (mGWAS) based on mixed linear models and found 152 genome-wide significant SNPs (*p*-value < 1.06 × 10^−6^) in association with 17 metabolites that included 90 significant SNPs annotated to 52 genes. On chromosome one alone, 51 significant SNPs associated with isovalerylcarnitine and propionylcarnitine were found to be in strong linkage disequilibrium (LD). SNPs in strong LD annotated to *FBXL4*, and *CCNC* consisted of two haplotype blocks where three SNPs (ALGA0004000, ALGA0004041, and ALGA0004042) were in the intron regions of *FBXL4* and *CCNC*. The interaction network revealed that *CCNC* and *FBXL4* were linked by the hub gene *N6AMT1* that was associated with isovalerylcarnitine and propionylcarnitine. Moreover, three metabolites (i.e., isovalerylcarnitine, propionylcarnitine, and pyruvic acid) were clustered in one group based on the low-high RFI pigs. This study performed a comprehensive metabolite-based genome-wide association study (GWAS) analysis for pigs with differences in feed efficiency and provided significant metabolites for which there is significant genetic variation as well as biological interaction networks. The identified metabolite genetic variants, genes, and networks in high versus low feed efficient pigs could be considered as potential genetic or biomarkers for feed efficiency.

## 1. Introduction

Large populations are generally essential for genome-wide association study (GWAS) to obtain sufficient statistical power for the identification of genetic polymorphisms [1]. However, some intermediate phenotypes like metabolites could potentially avoid this problem, as they are directly involved in metabolite conversion modification [2,3]. As the end products of cellular regulatory processes, metabolites represent the ultimate response of biological systems associated with genetic changes, so metabolomics is considered the link between genotypes and phenotypes [4]. Metabolomics refers to the measurements of all endogenous metabolites, intermediates, and products of metabolism and has been applied to measure the dynamic metabolic responses in pigs [5,6] and dairy cows [7,8]. Additionally, metabolites could provide details of physiological state, so genetic variant-associated metabolites are expected to display larger effect sizes [9]. Gieger et al. (2008) firstly used metabolite concentrations as quantitative traits in association with genotypes and found their available applications in GWAS [9]. Do et al. (2014) [10] conducted GWAS using residual feed intake (RFI) phenotypes to identify single-nucleotide polymorphisms (SNPs) that explain significant variation in feed efficiency for pigs. Our previous study found two metabolites (i.e., α-ketoglutarate and succinic acid) in a RFI-related network of dairy cows which could represent biochemical mechanisms underlying variation for phenotypes of feed efficiency [8].

In this study, we aimed to identify genetic variants (SNP markers) affecting concentrations of metabolites and to reveal the biochemical mechanisms underlying genetic variation for pigs’ feed efficiency. Our study is based on two of our previously published papers and datasets used therein [6,11]. Briefly, the experiment consisted of 59 Duroc and 49 Landrace pigs with data on feed efficiency (RFI), genotype (PorcineSNP80 BeadChip) data, and metabolomic data (45 final metabolite datasets derived from liquid chromatography-mass spectrometry (LC-MS) system). While our previous studies only looked at metabolome-phenotype associations [6], we report an integrated systems genomics approach to identify quantitative trait loci (QTLs) or SNPs affecting metabolite concentration via metabolite GWAS methods (mGWAS), where each metabolite is itself a phenotype. To the best of our knowledge, this is the first study to link the genomics with metabolomics to identify significant genetic variants associated with known metabolites that differ in pigs with different levels of feed efficiency. Main aims of our study are as follows:

1. Find significant SNP markers associated with all the metabolites in the metabolomics dataset using mGWAS method and then reveal the biochemical mechanisms underlying genetic variation for porcine feed efficiency using 108 Danish pigs in low and high RFI conditions, genotyped by 68K PorcineSNP80 BeadChip array.
2. Annotate identified significant SNP markers to porcine genes.
3. Annotate metabolites and identify enriched metabolic pathways and gene-metabolite networks to find the potential biomarkers that were strongly associated with feed efficiency.

## 2. Results

### 2.1. First Component Score and Significant Metabolic Pathways of 45 Metabolites

**Figure 1:**
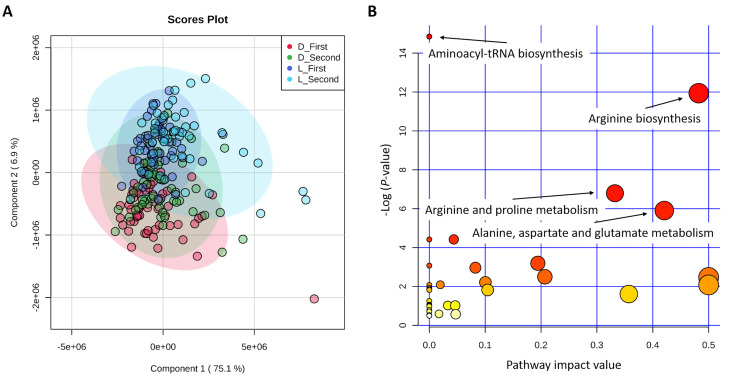
(**A**) Partial least squares-discriminant analysis (PLS-DA) of 45 metabolites. Note: D/L with first/second indicates the sampling time of Duroc/Landrace pigs. (**B**) Metabolic pathways for 45 metabolites using *Homo sapiens* as the library. Note: The size and color of the circles for each pathway indicate the matched metabolite ratio and the −log (*p*-value), respectively.

The partial least squares-discriminant analysis (PLS-DA) results revealed that the first component score (component 1) explained more than 75% variation of all 45 metabolites (Figure 1A). It showed that metabolite values of Duroc were higher than those of Landrace, the same as metabolites from second sampling time higher than those from first sampling time. In addition, the Duroc and Landrace pigs were clearly stratified, especially using the metabolite values between Duroc from first sampling time and Landrace from second sampling time (Figure 1A). The most significant metabolic pathways were the aminoacyl-tRNA biosynthesis; following by the arginine biosynthesis; the arginine and proline metabolism; and the alanine, aspartate, and glutamate metabolism (Figure 1B). As the pathway impact of the aminoacyl-tRNA biosynthesis was zero, we discarded this significant pathway and only used the metabolites enriched in the other three significant pathways for GWAS (Table 1). Thus, the metabolite means for 5 compounds in the arginine biosynthesis (arginine, aspartic acid, citrulline, glutamic acid, and ornithine); 5 compounds in the arginine and proline metabolism (arginine, glutamic acid, ornithine, proline, and pyruvic acid); and 4 compounds in the alanine, aspartate, and glutamate metabolism (alanine, aspartic acid, glutamic acid, and pyruvic acid) metabolites were calculated and shown in Table 1.

### 2.2. Genome-Wide Significant SNPs and Gene Annotation

**Figure 2:**
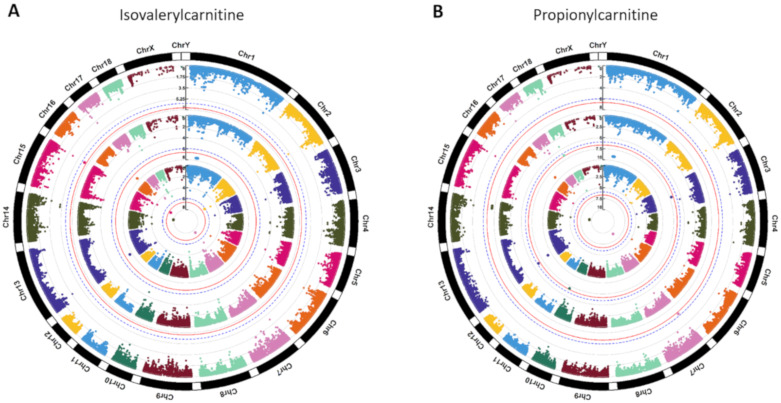
Manhattan plots of genome-wide association for (**A**) isovalerylcarnitine and (**B**) propionylcarnitine. Note: *Y*-axis indicates the log_10_ (*p*-value). Blue dotted and red solid lines indicate the genome-wide threshold of 0.05 and 0.01 after Bonferroni multiple testing, respectively. The three tracks indicate the metabolites from first sampling time, second sampling time, and combined two sampling times from outside to inside.

Metabolite based GWAS for first, second, and combined two sampling times revealed 152 genome-wide significant SNPs (Supplementary Table S1) in association with 17 metabolites (Supplementary Table S2). Unfortunately, no significant SNP was detected in association with first component scores (*p*-values ≥ 2.78 × 10^−6^) and metabolites enriched in the significant metabolic pathways (*p*-values ≥ 1.74 × 10^−4^); thus, GWAS results of these two scenarios were not listed. Manhattan plots of genome-wide association for isovalerylcarnitine and propionylcarnitine are shown in Figure 2, and Manhattan plots for the other 43 metabolites are shown in the Supplementary Figure S1. Along the whole genome, significant SNPs associated with isovalerylcarnitine and propionylcarnitine from the second sampling time were mainly located on the chromosome one (Figure 2). The overlapped significant SNPs associated with more than two different metabolites were shown in Table 2, where 57 significant SNPs on genome level were associated with isovalerylcarnitine and propionylcarnitine from the second sampling time. In addition, another 3 metabolites (1-hexadecyl-sn-glycero-3-phosphocholine, 1-myristoyl-sn-glycero-3-phosphocholine, lysoPC (16:0)) were also significantly associated with 10 SNPs (Table 2). 

After annotation of significant SNPs to the neighboring genes and gene components (Sscrofa10.2/susScr3), we found that 90 significant SNPs were within a 500-kb window of 52 neighboring genes (Supplementary Table S1) and that 6 significant SNPs were directly located in the gene components of 5 genes (Table 3). For example, if we only consider the SNPs on chromosome one, we found 29 significant SNPs were near 9 genes (Supplementary Table S1), whereas ALGA0004000, ALGA0004041, and ALGA0004042 were located in the introns of *FBXL4* and *CCNC* (Table 3). These results show that these genes may be involved in regulating abundance of the metabolites that are significantly different between high and low RFI pigs. Between using porcine RefSeq database of Sscrofa10.2/susScr3 and Sscrofa11.1/susScr11, the results of significant SNPs annotated to the genes overlapped greatly, but SNPs had different distances to the annotated genes between two versions (Supplementary Table S1). In Table 3, we found that the annotations of ALGA0004042 and ALGA0061605 to *CCNC* and *MTRF1* were changed from 9th intron and 5th intron to 8th intron and 9th intron, respectively, when we used the Sscrofa11.1/susScr11 database.

**Figure 3:**
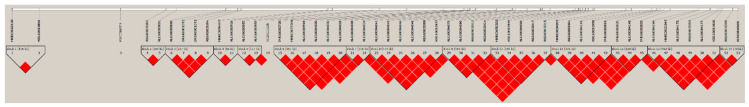
Linkage disequilibrium (LD) pattern for 53 significant SNPs on chromosome one. Note: the solid line triangle indicates LD. One square indicates LD level (r^2^) between two SNPs, and the squares are colored by the D’ & LLOR standard scheme. D’ & LLOR standard scheme is that red indicates LLOR > 2, D’ = 1; pink indicates LLOR > 2, D < 1; blue indicates LLOR < 2, D’ = 1; and white indicates LLOR < 2, D’ < 1. LLOR is the logarithm of likelihood odds ratio and the reliable index to measure D’.

The linkage disequilibrium (LD) pattern for all significant SNPs is shown in the Supplementary Figure S2. From the LD results for 58 significant SNPs on chromosome one, we found that 51 significant SNPs associated with isovalerylcarnitine (*p*-value = 2.79 × 10^−8^) and propionylcarnitine (*p*-value = 8.32 × 10^−10^) from second sampling time were in strong LD (Figure 3). Among the 58 significant SNPs, five of them were not in LD with the other 53 significant SNPs (Supplementary Figure S2), so they were excluded in the haplotype visualization in the Figure 3. In detail, SNPs annotated to *LOC780435* (NM_001078684), *FHL5* (NM_001243314), *FBXL4* (NM_001171752), *CCNC* (NM_001190160)/*MCHR2* (NM_001044609), and *SIM1* (NM_001172585) were in block 2, block 4, block 6, block 8, and block 9/10, respectively. Furthermore, ALGA0004000 in the 6th intron of *FBXL4* was in LD of block 6, together with another five SNPs (INRA0002726, MARC0075306, ALGA0003995, ALGA0004002, and ALGA0004005) that were located in the intergenic regions of *FBXL4*. Especially, three SNPs in strong LD consisted of block 8 with two SNPs (ALGA0004041 and ALGA0004042) located in the second and ninth intron of *CCNC* (Figure 3, Table 3, and Supplementary Table S1). The number of significant SNPs in strong LDs of the other chromosomes was less than the significant SNP number on chromosome one (Supplementary Figure S2). Notably, MARC0110390 in the 7th intron region of *SFXN1* (NM_001098602) on chromosome two and ALGA0061605 in the 5th intron region of *MTRF1* (NM_001243580) on chromosome eleven were not in the LD with other SNPs. However, ASGA0093565 in the 8th intron region of *DNAJC6* (NM_001145378) was in strong LD with WU_10.2_6_135312468 that was annotated to *LEPROT* (NM_001145388) (Supplementary Table S1 and Supplementary Figure S2).

### 2.3. Gene and Metabolite Interaction Network

**Figure 4:**
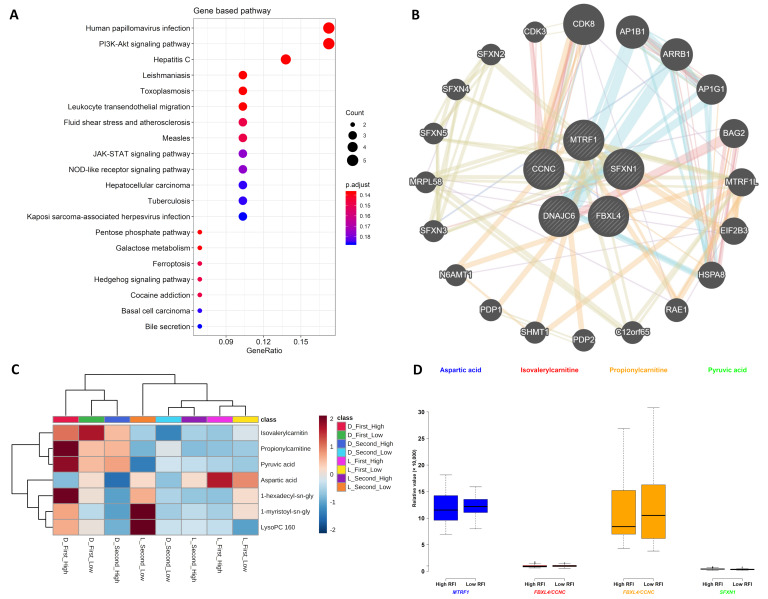
Gene pathway, metabolite cluster, and the interaction network: (**A**) Pathway for significant SNP-related genes. (**B**) Network for the genes in which significant SNPs were annotated to gene components. (**C**) Heatmap cluster for the metabolites that were associated with significant SNPs annotated to gene components. (**D**) Metabolites (i.e., aspartic acid, isovalerylcarnitine, propionylcarnitine, and pyruvic acid) values in high and low residual feed intake (RFI) pigs associated with the genes in which significant SNPs annotate to gene components. Note: The high RFI pigs and low RFI pigs were from left and right parts of all the pigs (n = 108) with one SD of actual RFI values.

The most significantly enriched gene-based pathways were the human papillomavirus infection (ssc05165) with five genes (i.e., *CCND2*, *CTNNB1*, *JAK1*, *LAMC1*, and *NFKB1*), followed by the PI3K-Akt signaling pathway (ssc04151) with five genes (i.e., *CCND2*, *F2R*, *JAK1*, *LAMC1*, and *NFKB1*) and the hepatitis C (ssc05160) with four genes (i.e., *CLDN8*, *CTNNB1*, *JAK1*, and *NFKB1*) (Figure 4A). Based on the gene–gene interaction network analysis, *CCNC* was in good connection with *CDK8*, *CDK3*, and *N6AMT1* whereas *N6AMT1* was linked to *FBXL4* (Figure 4B). Unfortunately, no gene–metabolite interaction network was found in this study. After the clustering of the SNP-related gene component-associated metabolites (Table 3), we found that aspartic acid, 1-hexadecyl-sn-glycero-3-phosphocholine, 1-myristoyl-sn-glycero-3-phosphocholine, and lysoPC(16:0) were clustered in the lower cluster while the upper cluster included the metabolites of isovalerylcarnitine, propionylcarnitine, and pyruvic acid (Figure 4C). Results show that metabolites from Duroc pigs have higher values in the upper cluster than those from lower cluster, but the metabolite values of Landrace pigs are higher in the lower cluster (Figure 4C). Afterwards, we investigated the metabolite values of aspartic acid, isovalerylcarnitine, propionylcarnitine, and pyruvic acid for which the associated significant SNPs were in the introns of *MTRF1*, *FBXL4*/*CCNC*, *SFXN1* (Table 3). Generally, propionylcarnitine from the low RFI group had higher values while other three metabolite values in the high RFI group seemed higher, but they are not significantly different between low and high RFI groups (*p*-value > 0.05) (Figure 4D).

## 3. Discussion

### 3.1. Metabolites in the PLS-DA and Metabolic Pathways of Pigs

The previous study reported that different breed types performed differently in RFI variation [8], so RFI-related metabolomics could be breed specific. Therefore, different breeds tend to exhibit different metabolite abundance values, for example, in studies involving the colostrum of pigs [12,13], the milk and plasma of cattle [8,14], the yolk and albumen of chickens [15,16], the plasma of dogs [17], and the fruit metabolite content of tomatoes [18]. In pigs, the heritability and genetic correlation of production traits of Duroc, Landrace, and Yorkshire pigs vary. Duroc pigs showed lower heritability of feed efficiency but greater performance of growth traits [19,20]. The metabolomics of this study showed that metabolite values vary between two pig breeds and between the sampling times (Figure 1A and Figure 4C), as the metabolite profiles would change according to the breeds and time points [6]. Metabolites of Duroc from first sampling time and Landrace from second sampling time were apparently stratified, probably because metabolite values of these two groups and their metabolite profiles were different. However, metabolites of Duroc from second sampling time and Landrace from first sampling time were very close, probably because metabolite values of these two groups and their metabolite profiles were very similar (Figure 1A). Hence, the breed differences between Duroc and Landrace pigs were driven both by genetic and metabolic factors.

The arginine biosynthesis pathway (ssc00220), where arginine, aspartic acid, citrulline, glutamic acid, and ornithine were significantly enriched in our study (Table 1), plays a crucial role in amino acid metabolism, particularly in the synthesis of citrulline and proline in pigs [21]. By linking arginine, glutamate, and proline in a bidirectional way, the arginine and proline metabolism pathway (ssc00330) biosynthesizes arginine and proline by glutamate. It is observed that proline metabolism is associated with metastasis formation of breast cancer [22]. In dairy cattle, the alanine, aspartate, and glutamate metabolism (ssc00250) identified in the gene-based pathways of our study (Table 1) is the potential metabolic biomarker between the low and high feed efficient conditions [8].

### 3.2. Genome-Wide Significant SNP-Related Genes Associated with Metabolites

The previous GWAS for Duroc pigs identified two pleiotropic QTLs on chromosome one and seven for feed efficiency [20]. Do et al. (2014) [10] revealed 19 significant SNPs located on several chromosomes (e.g., one, three, seven, eight, nine, ten, fourteen, and fifteen) that were highly associated with feed efficiency in Yorkshire pigs. In addition, other studies also found significant SNPs associated with RFI on other chromosomes, for example, SNPs on chromosome five in the growing Piétrain–Large White pigs [23], on chromosome two in a crossed populations [24], on chromosome six in Large White pigs [25], etc. [26,27].

In this study, significant SNPs were mainly located on chromosome one (58/152), but the associated metabolites only mapped to 1-hexadecyl-sn-glycero-3-phosphocholine (1.7%), 1-myristoyl-sn-glycero-3-phosphocholine (1.7%), isovalerylcarnitine (47.0%), isoleucyl proline (0.9%), propionylcarnitine (47.0%), and lysoPC(16:0) (1.7%). Obviously, isovalerylcarnitine and propionylcarnitine primarily derived from amino acid catabolism were the major metabolites that associated with nine significant SNP-related genes (i.e., *CCNC*, *FBXL4*, *FHL5*, *LOC780435*, *MAT2B*, *MCHR2*, *PNISR*, *RRAGD*, and *SIM1*) on chromosome one (Supplementary Table S1). A previous study indicated that the amount of isovalerylcarnitine could decrease in the plasma and liver tissues but greatly increased in the muscle tissue, as a byproduct of leucine catabolism [28]. The isovalerylcarnitine compound was reported to be found in high amounts in the colostrum and milk of sows [29]. As a key role in the mitochondrial fatty acid transport and high-energy phosphate exchange, propionylcarnitine could improve cardiac dysfunction by reducing myocardial ischaemia [30].

### 3.3. Gene and Metabolite Interaction Network

Based on the gene interaction node *N6AMT1*, one gene–gene interaction was found to connect *CCNC* with *FBXL4* (Figure 4B), in which significant SNPs were annotated to gene components and associated with isovalerylcarnitine and propionylcarnitine (Table 3). As the members of *CDK8* mediator complex that can regulate β-catenin-driven transcription, *CCNC* encodes the cell cycle regulatory protein cyclin C and results in the protein dysfunction due to the mutations of *CCNC* [31,32]. *CCNC* is also believed to increase the quiescent cells to maintain *CD34* expression after knocking down *CCNC* expression in human cord blood [33], while the amplification of *CCNC* was in a relationship with the unfavorable prognosis [34]. *FBXL4* is considered to participate in oxidative phosphorylation, mitochondrial dynamics, cell migration, prostate cancer metastasis, circadian GABAergic cyclic alteration, etc. [35,36,37,38,39]. The association results in pigs found that blood and immune traits were associated with the SNPs of *FBXL4* [40]. The node *N6AMT1* is responsible for DNA 6mA methylation modification as the first glutamine-specific protein methyltransferase characterized in mammals; thus, glutamine could be regulated by the genes that promote porcine growth performance [41,42].

### 3.4. Associations Linking SNP Genotypes, Metabolites, and RFI

To investigate the direct association between SNP genotypes and RFIs, we also conducted GWAS for RFI (i.e., where the GWAS model included RFI as phenotype and SNPs as fixed effect/explanatory variable) in the mixed linear model. Unfortunately but as expected due to small sample size, the results showed that no genome-wide significant associations were found between SNPs and RFI values (*p*-values ≥ 2.09 × 10^−4^). However, the top SNP was DRGA0008061 (*p*-values = 2.09 × 10^−4^), and we found five genes (*ANGPTL2*, *AUTS2*, *GRIFIN*, *PTRH1*, and *SIRT5*) in which the top ten SNPs were annotated (Supplementary Table S3). In our previous studies, Banerjee et al. (2020) [11] also revealed that DRGA0008061 was one of the top significant SNPs associated with RFI after genome-wide epistatic interaction network analysis for feed efficiency using the same genotypes and pig populations as used in our current study. Meanwhile, Carmelo et al. (2020) [6] used Kolmogorov–Smirnov test to identify the significant metabolites associated with feed efficiency traits at two time points in Duroc and Landrace pigs. They found that 1-hexadecyl-sn-glycero-3-phosphocholine, 1-myristoyl-sn-glycero-3-phosphocholine, isovalerylcarnitine, lysoPC(16:0), and phosphocholine were significantly (*p*-value < 0.05) associated with RFI and other feed efficiency traits [6]. By matching the results from Carmelo et al. (2020) [6] with our results, we found that these five metabolites were also our main significant SNP-associated findings in GWAS (Table 3). Therefore, the triangular association of genotypes (SNP), metabolomics (metabolite), and feed efficiency (RFI) is established via our mGWAS (SNPs affecting metabolites) and GWAS (SNPs affecting RFI) and is linked with the previous studies [6,11].

## 4. Materials and Methods

### 4.1. Animals, Metabolites, and Genotypes

A total of 108 pigs were involved in this study including 59 Duroc and 49 Landrace pigs that were part of our own previous published studies [6,11]. The detailed description of the animal experiment and phenotype, metabolite, and genotypes data collection are available from our previously published studies, and all data used in this study were derived from our datasets that were already made public. Metabolite data [6] were accessed using MetaboLights accession ID MTBLS1384 with a link: https://www.ebi.ac.uk/metabolights/MTBLS1384. Genotype data [11] were accessed from National Center for Biotechnology Information (NCBI) GEO accession number: GSE144064 with the following link: https://www.ncbi.nlm.nih.gov/geo/query/acc.cgi?acc=GSE144064. The genotype data was sequenced using GeneSeek-Neogen PorcineSNP80 BeadChip containing 68,528 loci based on the version Sscrofa10.2/susScr3 [11].

**Figure 5:**
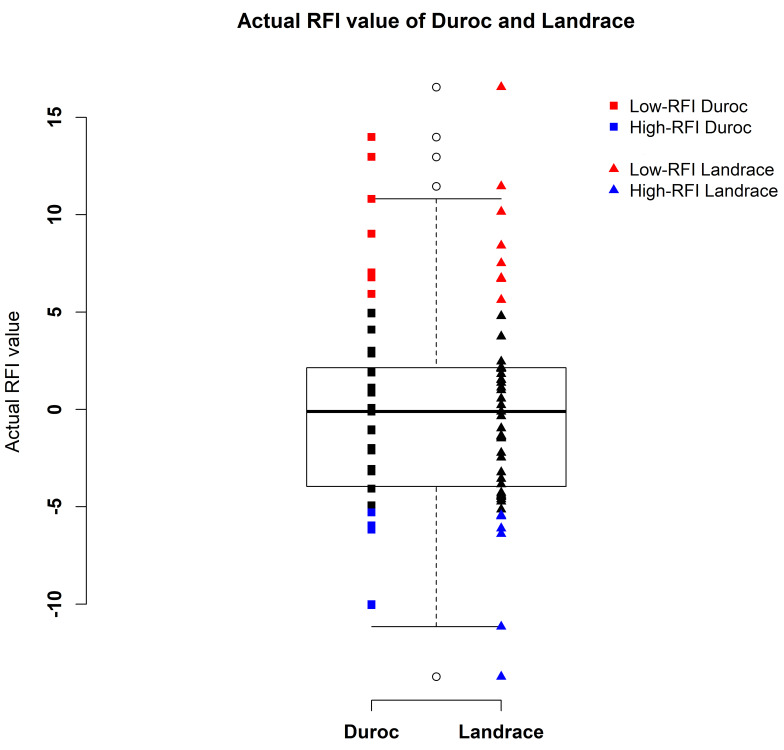
Distribution of actual RFI values of Duroc (n = 59) and Landrace (n = 49) pigs.

**Figure 6:**
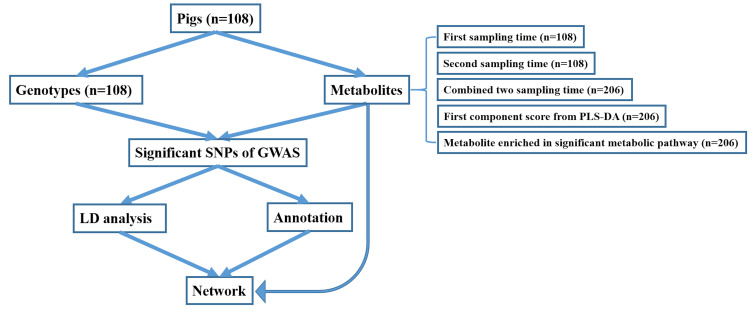
Overall analysis workflow.

As in Carmelo et al. (2020) [6], all the pigs were purebred uncastrated males derived from sixteen-sire families in four generations and fed on the same diets. They had RFI values calculated for each pig as the difference between the observed daily feed intake (DFI) and the predicted daily feed intake (pDFI) [6] following the method of Nguyen et al. (2001) [43]. Nguyen et al. (2001) [43] firstly corrected the DFI for batch and sex and their interaction effects (i.e., fixed effects) and then estimated the pDFI from different regression models including growth rate and back fat after adjustments for above fixed effects; hence, Carmelo et al. (2020) [6] could compute RFIs in the same way by correcting fixed effects in their study. Finally, our study directly used RFIs together with other phenotypes by accessing the public dataset with a link: https://www.ebi.ac.uk/metabolights/MTBLS1384. The range of actual RFI values of Duroc were from −10 to 14, while Landrace’s RFI value range was from −14 to 17 (Figure 5). The previous study conducted the metabolite–trait association analysis for RFI, so it was suggested that fatness or other factors should be adjusted in the calculation of RFI to achieve more accurate association results in their study [6]. As similar means of RFI for Duroc and Landrace pigs were observed in Figure 5 of our study, we assumed that fatness was adjusted in the calculation of RFI, but we cannot determine it. In this study, we selectively chose the extreme left and extreme right tails of distribution of feed efficiency (i.e., actual RFI values) distribution of all the pigs (n = 108) with one standard deviation (SD) from the mean [11,44] of actual RFI values. Then, they were defined as high RFI pigs (RFI ≤ −5.23, n = 11) and low RFI pigs (RFI ≥ 5.23, n = 16), respectively (Figure 5). The overview of the analysis workflow is shown in Figure 6 and included five scenarios of phenotypes in the GWAS analysis based on different transformations of metabolites. The five types of phenotypes were (1) the metabolites from first sampling time, (2) the metabolites from second sampling time, (3) the metabolites from combined two sampling times (i.e., metabolite values from first and second sampling times were combined as an integrated dataset, where each pig had two metabolic values for one metabolite, but genotypes were same for the metabolite values between first and the second sampling times from the same pig), (4) the first component score (component 1) from partial least squares-discriminant analysis (PLS-DA), and (5) the metabolites enriched in the significant metabolic pathways (Figure 6).

**Figure 7:**
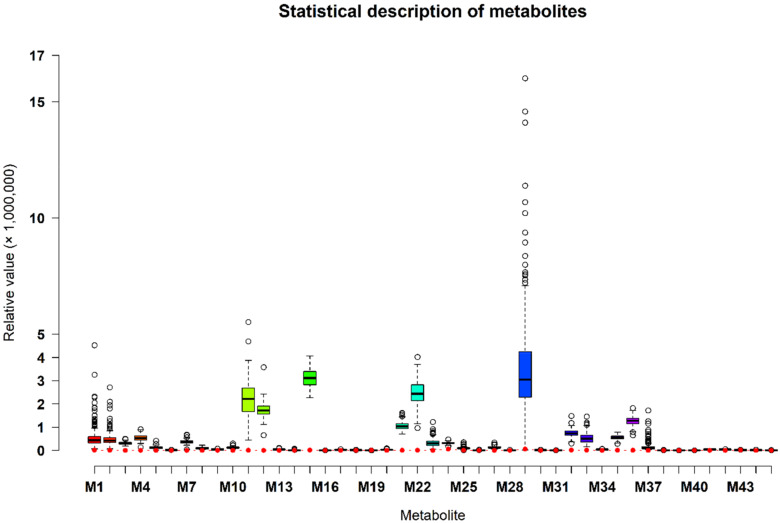
Statistical description of 45 metabolites from combined two sampling times. Note: The red solid circle indicates the limit of detection (LOD) relative value of each metabolite. LOD refers to the lowest value of a metabolite that the LC-MS method can detect. M1 to M45 indicate the metabolites of 1-hexadecyl-sn-glycero-3-phosphocholine, 1-myristoyl-sn-glycero-3-phosphocholine, (3-Carboxypropyl)trimethylammonium, 4-aminobenzoic acid, 5-methyl-5,6-dihydrouracils, acetaminophen, acetylcarnitine, alanine, arginine, aspartic acid, benzoic acid, carnitine, citrulline, cotinine, creatinine, cytidine, disaccharide, glutamic acid, guanine, guanosine, hypoxanthine, indoleacrylic acid, inosine, isoleucine, isoleucyl proline, isovalerylcarnitine, lactic acid, leucyl methionine, lysoPC(16:0), manNAc, methionine, monosaccharide, nicotine amide, ornithine, phenylalanine, proline, propionylcarnitine, pyruvic acid, riboflavine, sorbitol, thiamine, threonine, thymidine, uridine, and xanthine, respectively.

Metabolite data was downloaded by accessing MetaboLights accession ID MTBLS1384 with a link, https://www.ebi.ac.uk/metabolights/MTBLS1384, and were collected in two sampling times (i.e., the first sampling time was at the age when pig weighted approximately 28 kg, and the second sampling time was 45 days after the first sampling time) for each pig by the previous study [6]. Finally, 45 metabolites were used in this study (Figure 7) including 16 annotated metabolites (i.e., 1-hexadecyl-sn-glycero-3-phosphocholine, 1-myristoyl-sn-glycero-3-phosphocholine, (3-Carboxypropyl)trimethylammonium, 5-methyl-5,6-dihydrouracils, acetaminophen, acetylcarnitine, benzoic acid, cotinine, creatinine, indoleacrylic acid, isoleucyl proline, isovalerylcarnitine, leucyl methionine, lysoPC(16:0), manNAc, and propionylcarnitine) and 29 identified metabolites (i.e., 4-aminobenzoic acid, alanine, arginine, aspartic acid, carnitine, citrulline, cytidine, disaccharide, glutamic acid, guanine, guanosine, hypoxanthine, inosine, isoleucine, lactic acid, methionine, monosaccharide, nicotine amide, ornithine, phenylalanine, proline, pyruvic acid, riboflavine, sorbitol, thiamine, threonine, thymidine, uridine, and xanthine).

The genotype data was downloaded from NCBI GEO database with accession number: GSE144064 with a link, https://www.ncbi.nlm.nih.gov/geo/query/acc.cgi?acc=GSE144064, that was issued by the previous study [11]. After removing the markers with duplicated SNP positions (i.e., coordinates) (n = 274), unannotated SNP positions (n = 2618), and no genotypes (n = 3903) from GeneSeek-Neogen PorcineSNP80 BeadChip (68,516 SNP markers here), 61,721 SNP markers remained. Afterwards, we performed the imputation for missing markers using pedigree (i.e., all the pigs were derived from sixteen-sire families in four generations) by FImpute software (version 3) [45], as the closer relatives usually share longer haplotypes; therefore, pedigree information could contribute towards the FImpute software, achieving more accurate imputation [45,46]. Quality control (QC) for the imputed genotypes was conducted based on the criteria of Hardy–Weinberg equilibrium (HWE > 10^−7^) and minor allele frequency (MAF ≥ 0.001) by PLINK software (version 1.9) [47].

In this study, we also combined the metabolite values from the first sampling time and the second sampling time as an integrated dataset, so each pig had two values in one metabolite. However, the genotypes for two metabolite values were the same if one metabolite value was from the first sampling time of one pig while the other metabolite value was from the second sampling time of the same pig. In other words, each pig had two different metabolite values but the same genotypes; thus, QC results of the integrated dataset (n = 206) were different from the unintegrated dataset (n = 108), especially for HWE but not for MAF. Finally, the genotypes for the first sampling time and the second sampling time retained 47,109 SNP markers after removing unqualified 9337 (HWE ≤ 10^−7^) and 5275 (MAF < 0.001) markers, while the genotypes for the combined two sampling times retained 42,393 SNP markers after removing unqualified 14,053 (HWE ≤ 10^−7^) and 5275 (MAF < 0.001) markers.

### 4.2. Partial Least Squares-Discriminant Analysis and Metabolic Pathway Enrichment for 45 Metabolites

The partial least squares-discriminant analysis (PLS-DA) and metabolic pathway analysis for the 45 metabolites were performed by *MetaboAnalyst* software (version 4.0) [48] using *Homo sapiens* as the library. Fishers’ exact test and relative betweenness centrality were used for the overrepresented analysis and the pathway impact value calculation (i.e., sum of importance measures of the matched metabolites divided by the sum of the importance measures of all the metabolites), respectively [49]. The first component scores (component 1) and metabolites enriched in the significant metabolic pathways after false discovery rate (FDR) correction of multiple hypothesis testing (FDR < 0.1) were selected as phenotypes of the transformed metabolites for GWAS. The mean calculated for the metabolites enriched in each significant metabolic pathway was considered as transformed metabolite values; thus, each pathway had one transformed metabolite value (i.e., the mean).

### 4.3. Mixed Linear Model Based Association Analysis

In this study, we considered other environmental factors (e.g., age) the same among all the pigs, so we only used breed and RFI as the fixed effects to directly link metabolites with genotypes. GWAS for 45 single metabolites and transformed metabolites (i.e., component 1 and enriched metabolites) was conducted by mixed linear model based association analysis in GCTA software (version 1.93.0) [50]. The mixed linear model is as follows:

(1) y =Xb+g+e,

where y is the vector of phenotypes (i.e., metabolites from the first, second, and combined two sampling times and the transformed metabolites); b is the vector of fixed effects including intercept, breed effects (i.e., Duroc and Landrace pigs), RFI effects (i.e., actual RFI values included as covariates), and SNP effects (i.e., candidate SNPs included as covariates) to be tested; X is the design matrix for fixed effects that includes SNP genotype indicators (i.e., 0, 1, or 2); g is the vector of polygenic effects as random effects that are the accumulated effects of all SNPs; and e is the vector of residual effects. The polygenic and residual variances are $Var\left[g\right]=G\sigma_{g}^{2}$ and $Var\left[e\right]=I\sigma_{e}^{2}$, where G and I are the genetic relationship matrix (GRM) calculated using all SNPs and identity matrix, respectively.

### 4.4. Significant SNPs and Their Annotated Genes

The significant SNPs for GWAS were defined when the *p*-values were less than the threshold after Bonferroni correction for multiple hypothesis testing on genome level. The threshold for metabolites from the first and second sampling times was 1.06 × 10^−6^ (i.e., 0.05/47109), while the threshold for combined two sampling times was 1.18 × 10^−6^ (i.e., 0.05/42393). Then, the significant SNPs were annotated to the genes and gene components (i.e., promoters, exons, and introns) of porcine RefSeq database (Sscrofa10.2/susScr3) downloaded from University of California Santa Cruz (UCSC) genome browser (https://genome.ucsc.edu/cgi-bin/hgTables), where a window of 500 kb was used for the annotation of intergenic regions to neighboring genes. In addition, we used the reference SNP (rsfSNP) ID (i.e., specific rs number) of significant SNPs to annotate them to the genes and gene components of latest porcine RefSeq database (Sscrofa11.1/susScr11).

Linkage disequilibrium (LD) analysis to display the potential haplotypes for the significant SNPs was performed using Haploview software (version 4.2) [51]. SNPs with a distance larger than 500 kb were ignored in the pairwise comparisons for LD analysis.

### 4.5. Gene-Based Pathway Enrichment Analysis and Gene–Metabolite Interaction Network

We used R package *KEGG.db* (version 3.2.3) of *Sus scrofa* species to annotate SNP-related genes in the gene-based pathway enrichments. Based on the adjusted *p*-values (p.adjust) < 0.2 under FDR control, the gene-based pathways were finally realized by R package *clusterprofiler* (version 3.12.0) [52]. The gene–gene interaction networks were created by *GeneMANIA* server [53,54] with default settings using *Homo sapiens* as the library. Then, the gene–metabolite networks for interactions between SNP-related genes and phenotype-related metabolites were created by *MetaboAnalyst* tool [55] with default settings using the same library of *Homo sapiens*. Significant SNP-associated metabolites based on the low-high RFI pigs were hierarchically clustered by Ward’s method in Euclidean distance [56]. Then, a heat map for averaged metabolite clustering was visualized by *MetaboAnalyst* tool [48].

## 5. Conclusions

We utilized metabolic and genomic datasets from a total of 108 pigs that were made available for this study from our own previously published studies [6,11] in publicly available data repositories. These studies involved 59 Duroc and 49 Landrace pigs and consisted of data on feed efficiency (RFI), genotype (PorcineSNP80 BeadChip) data, and metabolomic data (45 final metabolite datasets derived from LC-MS system). Utilizing these datasets, our main aim was to identify genetic variants (SNPs) that affect 45 different metabolite concentrations in plasma collected at the start and end of the performance testing of pigs categorized as high or low in their feed efficiency, as measured by RFI values. Genome-wide significant genetic variants could be then used as potential genetic or biomarkers in breeding programs for feed efficiency. In order to achieve this main objective, we performed GWAS in the mixed linear model-based association analysis and found 152 genome-wide significant snps (*p*-value < 1.06 × 10^−6^) in association with 17 metabolites that included 90 significant SNPs annotated to 52 genes. On chromosome one alone, we found SNPs in strong LD that could be annotated to *FBXL4* and *CCNC*; it consisted of two haplotype blocks, where three SNPs (ALGA0004000, ALGA0004041, and ALGA0004042) were in the intron regions of *FBXL4* and *CCNC*. The interaction network analyses revealed that *CCNC* and *FBXL4* were linked to each other by *N6AMT1* gene and were associated with compounds isovalerylcarnitine and propionylcarnitine. The identified genetic variants and genes affecting important metabolites in high versus low feed efficient pigs could be considered as potential genetic or biomarkers, but we recommend that these results are validated in much higher sample size.

## Figures and Tables

**Table 1:** Significant metabolic pathways (False Discovery Rate (FDR) < 0.1) using *Homo sapiens* as the library.

Metabolic Pathway	Match Status	Involved Metabolites	*p*-Value	−Log (*p*-Value)	False Discovery Rate (FDR)	Pathway Impact Value
Aminoacyl-tRNA biosynthesis (ssc00970)	9/48	Alanine, Arginine, Aspartic acid, Glutamic acid, Isoleucine, Methionine, Phenylalanine, Proline, Threonine	3.55 × 10^−7^	14.85	2.98 × 10^−5^	0
Arginine biosynthesis (ssc00220)	5/14	Arginine, Aspartic acid, Citrulline, Glutamic acid, Ornithine	6.53 × 10^−6^	11.94	2.74 × 10^−4^	0.48
Arginine and proline metabolism (ssc00330)	5/38	Arginine, Glutamic acid, Ornithine, Proline, Pyruvic acid	1.12 × 10^−3^	6.79	0.031	0.33
Alanine, aspartate and glutamate metabolism (ssc00250)	4/28	Alanine, Aspartic acid, Glutamic acid, Pyruvic acid	2.72 × 10^−3^	5.91	0.057	0.42

**Table 2:** Common significant single-nucleotide polymorphisms (SNPs) of genome-wide association for more than two different metabolites from first, second, and combined two sampling times.

Significant SNP Name	Associated Metabolite Number	Metabolite from First Sampling Time	Metabolite from Second Sampling Time	Metabolite from Combined Two Sampling Times
ALGA0003891, ALGA0003900, ALGA0003935, ALGA0003952, ALGA0003953, ALGA0003995, ALGA0004000, ALGA0004002, ALGA0004005, ALGA0004006, ALGA0004024, ALGA0004041, ALGA0004042, ALGA0004046, ALGA0004048, ALGA0004073, ALGA0004090, ALGA0004093, ALGA0004143, ALGA0004148, ALGA0004169, ALGA0004173, ALGA0004177, ASGA0003182, ASGA0003194, ASGA0003235, ASGA0003288, ASGA0003312, ASGA0003314, ASGA0003315, ASGA0003317, ASGA0003333, ASGA0003335, ASGA0057312, ASGA0083304, DRGA0000994, DRGA0001072, DRGA0001073, H3GA0001865, H3GA0001937, H3GA0001949, H3GA0001956, H3GA0001966, H3GA0046845, INRA0002726, INRA0002819, INRA0002820, INRA0002823, MARC0021047, MARC0027518, MARC0034307, MARC0050325, MARC0059407, MARC0063106, MARC0068954, MARC0075306, SIRI0000655	2	NA	Isovalerylcarnitine, Propionylcarnitine	NA
MARC0080116	2	Pyruvic acid	NA	Citrulline
ALGA0038416, ALGA0081238, DRGA0014486, WU_10.2_14_132246191	3	Isovalerylcarnitine, Propionylcarnitine	NA	Propionylcarnitine
ASGA0093565, H3GA0053559, WU_10.2_6_136216429, WU_10.2_6_136863547, WU_10.2_6_136876717, WU_10.2_6_136972846	3	1-hexadecyl-sn-glycero-3-phosphocholine, 1-myristoyl-sn-glycero-3-phosphocholine, LysoPC(16:0)	NA	NA
M1GA0016778, WU_10.2_X_114649203	3	Pyruvic acid	NA	Citrulline, Pyruvic acid
ALGA0099866, WU_10.2_X_105559450	4	1-hexadecyl-sn-glycero-3-phosphocholine, 1-myristoyl-sn-glycero-3-phosphocholine, LysoPC(16:0), Pyruvic acid	NA	NA
ASGA0018324	4	Citrulline, Pyruvic acid	NA	Citrulline, Pyruvic acid
ASGA0081223, INRA0003881, MARC0046138, WU_10.2_X_103597980, WU_10.2_X_103653646, WU_10.2_X_104796075, WU_10.2_X_104910069, WU_10.2_X_104956283, WU_10.2_X_104980830, WU_10.2_X_105583738	4	1-hexadecyl-sn-glycero-3-phosphocholine, 1-myristoyl-sn-glycero-3-phosphocholine, LysoPC(16:0)	NA	1-hexadecyl-sn-glycero-3-phosphocholine

Note: NA indicates not applicable.

**Table 3:** Gene component annotation for genome-wide significant SNPs.

Chromosome	Position	Significant SNP Name	Gene Component	Gene	Gene Description	Metabolite from First Sampling Time (*p*-Value)	Metabolite from Second Sampling Time (*p*-Value)	Metabolite from Combined Two Sampling Times (*p*-Value)
1	74467285	ALGA0004000	6th intron	*FBXL4* (NM_001171752)	F-box and leucine rich repeat protein 4	NA	Isovalerylcarnitine (2.79 × 10^−8^), Propionylcarnitine (8.32 × 10^−10^)	NA
1	75151870	ALGA0004041	1st intron	*CCNC* (NM_001190160)	Cyclin C	NA	Isovalerylcarnitine (2.79 × 10^−8^), Propionylcarnitine (8.32 × 10^−10^)	NA
1	75167426	ALGA0004042	9th intron/8th intron ^#^	*CCNC* (NM_001190160)	Cyclin C	NA	Isovalerylcarnitine (2.79 × 10^−8^), Propionylcarnitine (8.32 × 10^−10^)	NA
2	83663964	MARC0110390	7th intron	*SFXN1* (NM_001098602)	Sideroflexin 1	Pyruvic acid (1.25 × 10^−7^)	NA	NA
6	135424176	ASGA0093565	8th intron	*DNAJC6* (NM_001145378)	DnaJ heat shock protein family (Hsp40) member C6	1-hexadecyl-sn-glycero-3-phosphocholine (2.78 × 10^−9^), 1-myristoyl-sn-glycero-3-phosphocholine (1.35 × 10^−8^), LysoPC (16:0) (1.22 × 10^−7^)	NA	NA
11	26591544	ALGA0061605	5th intron/9th intron ^#^	*MTRF1* (NM_001243580)	Mitochondrial translation release factor 1	NA	Aspartic acid (2.29 × 10^−7^)	NA

Note: NA indicates not applicable. ^#^ The annotation of the latest porcine RefSeq database (Sscrofa11.1/susScr11).

## Data Availability

All datasets used in this paper are from public repositories. Metabolite data were accessed using MetaboLights accession ID MTBLS1384 with a link: https://www.ebi.ac.uk/metabolights/MTBLS1384. Genotype data were accessed from NCBI GEO accession number GSE144064 with the following link: https://www.ncbi.nlm.nih.gov/geo/query/acc.cgi?acc=GSE144064). The details of these datasets can be found in Carmelo et al. (2020) [6] and Banerjee et al. (2020) [11].

## Supplementary Materials

The following are available online at https://www.mdpi.com/2218-1989/10/5/201/s1, Supplementary Table S1. All the significant SNPs of genome-wide association with chromosome, position, and p-value information for metabolites from first, second, and combined two sampling times. Supplementary Table S2. All the metabolites in association with significant SNPs from first, second, and combined two sampling times. Supplementary Table S3. Top ten SNPs associated with residual feed intake (RFI). Supplementary Figure S1. Manhattan plots of genome-wide association for 43 metabolites. Supplementary Figure S2. Linkage disequilibrium (LD) pattern for all significant SNPs.

## Abbreviations

Component 1	First component score
DFI	Daily feed intake
FC	Fold change
FDR	False discovery rate
LC-MS	Liquid chromatography-mass spectrometry
GRM	Genetic relationship matrix
GWAS	Genome-wide association study
HWE	Hardy–Weinberg equilibrium
LD	Linkage disequilibrium
LOD	Limit of detection
LLOR	Logarithm of likelihood odd ratio
MAF	Minor allele frequency
mGWAS	Metabolite GWAS
NCBI	National Center for Biotechnology Information
pDFI	Predicted daily feed intake
PLS-DA	Partial least squares-discriminant analysis
QC	Quality control
QTL	Quantitative trait locus
RFI	Residual feed intake
SNP	Single nucleotide polymorphism
UCSC	University of California Santa Cruz
