# Supplementary material for: Metabolite Genome-Wide Association Study (mGWAS) and Gene-Metabolite Interaction Network Analysis Reveal Potential Biomarkers for Feed Efficiency in Pigs
Source: Metabolites. 2020 May 15;10(5):201. doi: 10.3390/metabo10050201 (PMC7281523; doi:10.3390/metabo10050201)
Supplement: Supplementary file 1 [file metabolites-10-00201-s001.pdf]

Supplementary Table S1. All the significant SNPs of genome-wide association with chromosome, position, and *p*-value information for metabolites from first, second, and combined two sampling times.

Significant SNP Name	Refsnp (Rs) Number	Chromosome	Annotation By Porcine Refseq Database (Sscrofa10.2/Susscr3)			Annotation By Porcine Refseq Database (Sscrofa11.1/Susscr11)			Associated Metabolite Number	Metabolite From First Sampling Time (*p*-Value)	Metabolite From Second Sampling Time (*p*-Value)	Metabolite From Combined Two Sampling Times (*p*-Value)
			Position	Nearest gene	Distance	Position #	Nearest gene #	Distance #				
18_2566820	NA	18	2566820	*SHH* (NM_001244513)	97973	NA	NA	NA	1	NA	Aspartic acid (2.29×10^{-7})	NA
2_29041727	NA	2	29041727	*CAT* (NM_214301)	140893	NA	NA	NA	1	NA	Nicotine amide (4.71×10^{-7})	NA
3_118102178	NA	3	118102178	*RBKS* (NM_001315776)	172300	NA	NA	NA	1	NA	Aspartic acid (2.29×10^{-7})	NA
ALGA0000375	rs80848743	1	5395131	NA	NA	4048051	NA	NA	1	NA	Isoleucyl proline (3.57×10^{-7})	NA
ALGA0014130	rs81360281	2	86940572	*F2R* (NM_001244372)	339212	85318127	*F2R* (NM_001244372)	324666	1	Pyruvic acid (1.25×10^{-7})	NA	NA
ALGA0043837	rs80954663	7	100079759	*SYNJ2BP* (NM_001244991)	149115	93767736	*SYNJ2BP* (NM_001244991)	141467	1	NA	Isoleucyl proline (3.57×10^{-7})	NA
ALGA0046501	rs81402045	8	12844147	*LCORL* (NM_001195345)	78106	13047394	*LCORL* (NM_001195345)	78259	1	NA	Isoleucyl proline (3.57×10^{-7})	NA
ALGA0046512	rs81402401	8	13111033	*LCORL* (NM_001195345)	344992	13253758	*LCORL* (NM_001195345)	284623	1	NA	Isoleucyl proline (3.57×10^{-7})	NA
ALGA0049375	rs81403821	8	127824811	*NFKB1* (NM_001048232)	160810	118606542	*NFKB1* (NM_001048232)	127615	1	Pyruvic acid (7.57×10^{-7})	NA	NA
ALGA0049385	rs81403842	8	127976364	*NFKB1* (NM_001048232)	312363	118693613	*NFKB1* (NM_001048232)	214686	1	Pyruvic acid (7.57×10^{-7})	NA	NA
ALGA0061605	rs80965301	11	26591544	*MTRF1* (NM_001243580)	5th intron	25626653	*MTRF1* (NM_001243580)	9th intron	1	NA	Aspartic acid (2.29×10^{-7})	NA

ALGA0072779	rs81449300	13	177981732	*EPHA3* (NM_001195335)	171617	167114056	*EPHA3* (NM_001195335)	171417	1	Cotinine (4.18×10^{-9})	NA	NA
ALGA0083708	rs80789894	15	2069154	NA	NA	2636979	NA	NA	1	NA	Guanine (1.63×10^{-7})	NA
ALGA0104701	rs81479307	17	8537481	NA	NA	7658710	NA	NA	1	NA	Guanine (8.32×10^{-8})	NA
ASGA0040302	rs81405358	8	143127778	*CDS1* (NM_001044534)	312027	133648103	*CDS1* (NM_001044534)	313671	1	1-Hexadecyl-sn-glycero-3-phosphocholine (7.02×10^{-7})	NA	NA
ASGA0042433	rs81408742	9	34828091	NA	NA	30764062	NA	NA	1	Pyruvic acid (6.93×10^{-7})	NA	NA
ASGA0042436	rs81408748	9	34883824	NA	NA	30819738	NA	NA	1	Pyruvic acid (6.93×10^{-7})	NA	NA
ASGA0054868	rs81435997	12	49919795	*MIR212* (NR_128427)	96355	48066819	*MIR132* (NR_128416)	95805	1	NA	Guanine (8.32×10^{-8})	NA
ASGA0078611	rs81468455	18	2676317	*SHH* (NM_001244513)	2512	2557956	*SHH* (NM_001244513)	2472	1	NA	Propionylcarnitine (2.98×10^{-7})	NA
ASGA0083287	rs81336677	13	3868357	*DPH3* (NM_001243616)	282233	3681442	*DPH3* (NM_001243616)	285586	1	Creatinine (4.79×10^{-7})	NA	NA
ASGA0094610	rs81313834	17	48307754	NA	NA	43011191	NA	NA	1	LysoPC(16:0) (5.29×10^{-7})	NA	NA
ASGA0095299	rs81314653	5	83455803	*TXNRD1* (NM_214154)	202024	80009588	*TXNRD1* (NM_214154)	160745	1	NA	NA	Monosaccharide (9.09×10^{-7})
DRGA0004012	NA	3	71708951	*DOK1* (NM_001143705)	39335	NA	NA	NA	1	NA	Propionylcarnitine (7.66×10^{-8})	NA
DRGA0008955	NA	8	143055109	*CDS1* (NM_001044534)	384696	NA	NA	NA	1	1-Hexadecyl-sn-glycero-3-phosphocholine (7.02×10^{-7})	NA	NA
H3GA0007606	rs81364346	2	132433332			127080032			1	Isovalerylcarnitine (7.84×10^{-7})	NA	NA
H3GA0015652	rs80945649	5	10709671	*MIR9834* (NR_128521)	331400	NA	NA	NA	1	NA	1-Myristoyl-sn-glycero-3-phosphocholine (5.74×10^{-9})	NA
H3GA0026947	rs81408744	9	34841808	NA	NA	30777755	NA	NA	1	Pyruvic acid (6.93×10^{-7})	NA	NA

H3GA0028115	rs81416160	9	128659759	NA	NA	116973667	NA	NA	1	NA	Isoleucyl proline (3.57×10^{-7})	NA
H3GA0035928	rs81443958	13	27911595	*CTNNB1* (NM_214367)	244338	25427151	*CTNNB1* (NM_214367)	218840	1	NA	Propionylcarnitine (5.78×10^{-7})	NA
H3GA0055935	rs81477237	9	2349391	NA	NA	1973555	NA	NA	1	NA	Aspartic acid (2.29×10^{-7})	NA
INRA0031485	rs345734573	9	34920723	NA	NA	30856635	NA	NA	1	Pyruvic acid (6.93×10^{-7})	NA	NA
M1GA0003090	rs81364347	2	132449420	NA	NA	127096117	NA	NA	1	Isovalerylcarnitine (7.84×10^{-7})	NA	NA
M1GA0015335	rs80873877	11	80613144	NA	NA	73179330	NA	NA	1	Cotinine (4.18×10^{-9})	NA	NA
MARC0001720	rs81287225	9	0	NA	NA	NA	NA	NA	1	Pyruvic acid (6.93×10^{-7})	NA	NA
MARC0010841	rs80986455	5	10759678	*MIR9834* (NR_128521)	381407	NA	NA	NA	1	NA	1-Myristoyl-sn-glycero-3-phosphocholine (5.74×10^{-9})	NA
MARC0023109	rs80900706	14	149350120	NA	NA	137446913	NA	NA	1	NA	Pyruvic acid (1.07×10^{-7})	NA
MARC0027232	rs80875525	17	48876667	*ZHX3* (NM_001258405)	371390	43476692	*ZHX3* (NM_001258405)	377480	1	LysoPC(16:0) (5.29×10^{-7})	NA	NA
MARC0040505	rs81233629	9	50293805	*FXYD2* (NM_001014427)	44876	45214687	*FXYD2* (NM_001014427)	44975	1	NA	NA	Citrulline (4.75×10^{-7})
MARC0060063	rs81248931	8	142707308	NA	NA	133228051	NA	NA	1	1-Hexadecyl-sn-glycero-3-phosphocholine (7.02×10^{-7})	NA	NA
MARC0083382	rs81266104	15	140091862	NA	NA	126592447	NA	NA	1		Carnitine (2.40×10^{-7})	NA
MARC0092124	rs80950563	9	34900287	NA	NA	30836201	NA	NA	1	Pyruvic acid (6.93×10^{-7})	NA	NA
MARC0110390	rs81238710	2	83663964	*SFXN1* (NM_001098602)	7th intron	82042106	*SFXN1* (NM_001098602)	7th intron	1	Pyruvic acid (1.25×10^{-7})	NA	NA
WU_10.2_10_71866847	rs324154547	10	71866847	*AKR1C2* (NM_001044570)	16292	65613895	*AKR1C2* (NM_001044570)	16294	1	NA	Propionylcarnitine (1.28×10^{-7})	NA

WU_10.2_12_49599992	rs340150298	12	49599992	*INPP5K* (NM_001190292)	3831	47715327	*INPP5K* (NM_001190292)	3829	1	NA	Guanine (8.32 × 10^{-8})	NA
WU_10.2_12_49979240	rs81436012	12	49979240	*MIR212* (NR_128427)	36910	48126255	*MIR132* (NR_128416)	36369	1	NA	Guanine (8.32 × 10^{-8})	NA
WU_10.2_13_204594763	rs319481912	13	204594763	*CLDN8* (NM_001161646)	428133	194009692	*CLDN8* (NM_001161646)	445602	1	NA	Isoleucyl proline (4.20 × 10^{-7})	NA
WU_10.2_13_204608919	rs339019578	13	204608919	*CLDN8* (NM_001161646)	442289	194023848	*CLDN8* (NM_001161646)	459758	1	NA	Isoleucyl proline (4.20 × 10^{-7})	NA
WU_10.2_13_204631833	rs334759865	13	204631833	*CLDN8* (NM_001161646)	465203	194046757	*CLDN8* (NM_001161646)	482667	1	NA	Isoleucyl proline (4.20 × 10^{-7})	NA
WU_10.2_17_48295280	rs336874688	17	48295280	NA	NA	42998719	NA	NA	1	LysoPC(16:0) (5.29 × 10^{-7})	NA	NA
WU_10.2_2_132463311	rs332147841	2	132463311	NA	NA	127110011	NA	NA	1	Isovalerylcarnitine (7.84 × 10^{-7})	NA	NA
WU_10.2_2_132503341	rs324002124	2	132503341	NA	NA	127150044	NA	NA	1	Isovalerylcarnitine (7.84 × 10^{-7})	NA	NA
WU_10.2_2_132570224	rs341398921	2	132570224	NA	NA	127217328	NA	NA	1	Isovalerylcarnitine (7.84 × 10^{-7})	NA	NA
WU_10.2_6_135312468	rs337696797	6	135312468	*LEPROT* (NM_001145388)	67384	146907252	*LEPR* (NM_001024587)	40467	1	1-Hexadecyl-sn-glycero-3-phosphocholine (1.05 × 10^{-6})	NA	NA
WU_10.2_7_100175430	rs323081013	7	100175430	*SYNJ2BP* (NM_001244991)	53444	93863399	*SYNJ2BP* (NM_001244991)	45804	1	NA	Isoleucyl proline (3.57 × 10^{-7})	NA
WU_10.2_8_12998407	rs318854532	8	12998407	*LOCRL* (NM_001195345)	232366	13177356	*LOCRL* (NM_001195345)	208221	1	NA	Isoleucyl proline (3.57 × 10^{-7})	NA
WU_10.2_8_142992366	rs340936427	8	142992366	*CDS1* (NM_001044534)	447439	133513075	*CDS1* (NM_001044534)	448699	1	1-Hexadecyl-sn-glycero-3-phosphocholine (7.02 × 10^{-7})	NA	NA
WU_10.2_9_128572226	rs346382890	9	128572226	NA	NA	116885399	NA	NA	1	NA	Isoleucyl proline (3.57 × 10^{-7})	NA

WU_10.2_X_104031384	rs323531807	X	104031384	NA	NA	90474090	*MIR652* (NR_049178)	307483	1	NA	Lactic acid (3.14×10^{-7})	NA
WU_10.2_X_37114281	rs335077227	X	37114281	*GP91-PHOX* (NM_214043)	253546	33462220	*GP91-PHOX* (NM_214043)	192435	1	NA	1-Hexadecyl-sn-glycero-3-phosphocholine (4.85×10^{-9})	NA
ALGA0003891	rs80933223	1	69946212	*LOC780435* (NM_001078684)	226447	61964395	NA	NA	2	NA	Isovalerylcarnitine (2.79×10^{-8}), Propionylcarnitine (8.32×10^{-10})	NA
ALGA0003900	rs80977812	1	70712755	NA	NA	62583607	NA	NA	2	NA	Isovalerylcarnitine (2.79×10^{-8}), Propionylcarnitine (8.32×10^{-10})	NA
ALGA0003935	rs80997522	1	72365650	*FHL5* (NM_001243314)	244726	63996109	*FHL5* (NM_001243314)	243959	2	NA	Isovalerylcarnitine (2.79×10^{-8}), Propionylcarnitine (8.32×10^{-10})	NA
ALGA0003952	rs80995170	1	72982828	NA	NA	64614854	NA	NA	2	NA	Isovalerylcarnitine (2.79×10^{-8}), Propionylcarnitine (8.32×10^{-10})	NA
ALGA0003953	rs80996334	1	73100657	NA	NA	64674794	NA	NA	2	NA	Isovalerylcarnitine (2.79×10^{-8}), Propionylcarnitine (8.32×10^{-10})	NA
ALGA0003995	rs80857181	1	74410201	*FBXL4* (NM_001171752)	12232	65859940	*FBXL4* (NM_001171752)	12232	2	NA	Isovalerylcarnitine (2.79×10^{-8}), Propionylcarnitine (8.32×10^{-10})	NA

ALGA0004000	rs80892026	1	74467285	*FBXL4* (NM_001171752)	6th intron	65918133	*FBXL4* (NM_001171752)	6th intron	2	NA	Isovalerylcarnitine (2.79 × 10^{-8}), Propionylcarnitine (8.32 × 10^{-10})	NA
ALGA0004002	rs80915364	1	74502751	*FBXL4* (NM_001171752)	32524	65953598	*FBXL4* (NM_001171752)	32523	2	NA	Isovalerylcarnitine (2.79 × 10^{-8}), Propionylcarnitine (8.32 × 10^{-10})	NA
ALGA0004005	rs80882527	1	74583833	*FBXL4* (NM_001171752)	113606	66034686	*FBXL4* (NM_001171752)	113611	2	NA	Isovalerylcarnitine (2.79 × 10^{-8}), Propionylcarnitine (8.32 × 10^{-10})	NA
ALGA0004006	rs80821723	1	74597318	*FBXL4* (NM_001171752)	127091	66047984	*FBXL4* (NM_001171752)	126909	2	NA	Isovalerylcarnitine (2.79 × 10^{-8}), Propionylcarnitine (8.32 × 10^{-10})	NA
ALGA0004024	rs80932534	1	74916127	*PNISR* (NM_001113439)	79963	66209303	*PNISR* (NM_001113439)	141665	2	NA	Isovalerylcarnitine (2.79 × 10^{-8}), Propionylcarnitine (8.32 × 10^{-10})	NA
ALGA0004041	rs80971041	1	75151870	*CCNC* (NM_001190160)	1st intron	66475147	*CCNC* (NM_001190160)	1st intron	2	NA	Isovalerylcarnitine (2.79 × 10^{-8}), Propionylcarnitine (8.32 × 10^{-10})	NA
ALGA0004042	rs80827883	1	75167426	*CCNC* (NM_001190160)	9th intron	66491115	*CCNC* (NM_001190160)	8th intron	2	NA	Isovalerylcarnitine (2.79 × 10^{-8}), Propionylcarnitine (8.32 × 10^{-10})	NA

ALGA0004046	rs81354598	1	75219602	*CCNC* (NM_001190160)	44247	66543287	*CCNC* (NM_001190160)	44243	2	NA	Isovalerylcarnitine (2.79 × 10^{-8}), Propionylcarnitine (8.32 × 10^{-10})	NA
ALGA0004048	rs80821579	1	75398012	*MCHR2* (NM_001044609)	165694	66590915	*CCNC* (NM_001190160)	91871	2	NA	Isovalerylcarnitine (2.79 × 10^{-8}), Propionylcarnitine (8.32 × 10^{-10})	NA
ALGA0004073	rs81354625	1	75749595	*MCHR2* (NM_001044609)	167075	66949768	*MCHR2* (NM_001044609)	92821	2	NA	Isovalerylcarnitine (2.79 × 10^{-8}), Propionylcarnitine (8.32 × 10^{-10})	NA
ALGA0004090	rs80898039	1	76136641	*SIM1* (NM_001172585)	65411	67360888	*SIM1* (NM_001172585)	67752	2	NA	Isovalerylcarnitine (2.79 × 10^{-8}), Propionylcarnitine (8.32 × 10^{-10})	NA
ALGA0004093	rs81354649	1	76340224	*SIM1* (NM_001172585)	268994	67641195	*SIM1* (NM_001172585)	348059	2	NA	Isovalerylcarnitine (2.79 × 10^{-8}), Propionylcarnitine (8.32 × 10^{-10})	NA
ALGA0004143	rs80897893	1	77325351	NA	NA	68447515	NA	NA	2	NA	Isovalerylcarnitine (2.79 × 10^{-8}), Propionylcarnitine (8.32 × 10^{-10})	NA
ALGA0004148	rs81354693	1	77518365	NA	NA	68642779	NA	NA	2	NA	Isovalerylcarnitine (2.79 × 10^{-8}), Propionylcarnitine (8.32 × 10^{-10})	NA

ALGA0004169	rs80831522	1	78021030	NA	NA	69035750	NA	NA	2	NA	Isovalerylcarnitine (2.79 × 10^{-8}), Propionylcarnitine (8.32 × 10^{-10})	NA
ALGA0004173	rs81354711	1	78233457	NA	NA	69196503	NA	NA	2	NA	Isovalerylcarnitine (2.79 × 10^{-8}), Propionylcarnitine (8.32 × 10^{-10})	NA
ALGA0004177	rs81354721	1	78389441	NA	NA	69419420	NA	NA	2	NA	Isovalerylcarnitine (2.79 × 10^{-8}), Propionylcarnitine (8.32 × 10^{-10})	NA
ALGA0028664	rs80782595	4	129539293	*RTCA* (NM_001243470)	35897	117912209	*RTCA* (NM_001243470)	36536	2	NA	Glutamic acid (3.57 × 10^{-10})	Glutamic acid (4.80 × 10^{-8})
ALGA0103368	rs81330345	15	150174862	NA	NA	135738984	NA	NA	2	Isovalerylcarnitine (9.57 × 10^{-8})	NA	Isovalerylcarnitine (4.19 × 10^{-7})
ASGA0003182	rs81354471	1	69781723	*LOC780435* (NM_001078684)	61958	61885245	NA	NA	2	NA	Isovalerylcarnitine (2.79 × 10^{-8}), Propionylcarnitine (8.32 × 10^{-10})	NA
ASGA0003194	rs80868692	1	70950692	NA	NA	62811729	NA	NA	2	NA	Isovalerylcarnitine (2.79 × 10^{-8}), Propionylcarnitine (8.32 × 10^{-10})	NA
ASGA0003235	rs80788846	1	73520354	NA	NA	64978117	NA	NA	2	NA	Isovalerylcarnitine (2.79 × 10^{-8}), Propionylcarnitine (8.32 × 10^{-10})	NA

ASGA0003288	rs81354599	1	75602201	*MCHR2* (NM_001044609)	19681	66876761	*MCHR2* (NM_001044609)	19814	2	NA	Isovalerylcarnitine (2.79 × 10^{-8}), Propionylcarnitine (8.32 × 10^{-10})	NA
ASGA0003312	rs80793011	1	76189831	*SIM1* (NM_001172585)	118601	67429429	*SIM1* (NM_001172585)	136293	2	NA	Isovalerylcarnitine (2.79 × 10^{-8}), Propionylcarnitine (8.32 × 10^{-10})	NA
ASGA0003314	rs80829508	1	76215472	*SIM1* (NM_001172585)	144242	67455613	*SIM1* (NM_001172585)	162477	2	NA	Isovalerylcarnitine (2.79 × 10^{-8}), Propionylcarnitine (8.32 × 10^{-10})	NA
ASGA0003315	rs81354645	1	76438140	*SIM1* (NM_001172585)	366910	67523140	*SIM1* (NM_001172585)	230004	2	NA	Isovalerylcarnitine (2.79 × 10^{-8}), Propionylcarnitine (8.32 × 10^{-10})	NA
ASGA0003317	rs81354653	1	76416357	*SIM1* (NM_001172585)	345127	67606390	*SIM1* (NM_001172585)	313254	2	NA	Isovalerylcarnitine (2.79 × 10^{-8}), Propionylcarnitine (8.32 × 10^{-10})	NA
ASGA0003333	rs81354719	1	78347698	NA	NA	69377677	NA	NA	2	NA	Isovalerylcarnitine (2.79 × 10^{-8}), Propionylcarnitine (8.32 × 10^{-10})	NA
ASGA0003335	rs80965801	1	78587646	NA	NA	69582940	NA	NA	2	NA	Isovalerylcarnitine (2.79 × 10^{-8}), Propionylcarnitine (8.32 × 10^{-10})	NA

ASGA0023013	rs80914682	4	135677951	*TMED5* (NM_001243695)	96353	123966445	*TMED5* (NM_001243695)	43379	2	NA	Glutamic acid (3.57 × 10^{-10})	Glutamic acid (4.80 × 10^{-8})
ASGA0025965	rs80915233	5	68149464	*C5H12orf4* (NM_001243412)	4228	65943570	*C5H12orf4* (NM_001243412)	4230	2	NA	Glutamic acid (3.57 × 10^{-10})	Glutamic acid (4.80 × 10^{-8})
ASGA0044546	rs81416666	9	133959774	*TOR1AIP2* (NM_001243389)	487430	122221003	*STX6* (NM_001243720)	312735	2	NA	Carnitine (1.24 × 10^{-8})	Carnitine (5.24 × 10^{-8})
ASGA0057312	rs81445015	13	40238900	NA	NA	36873361	NA	NA	2	NA	Isovalerylcarnitine (2.79 × 10^{-8}), Propionylcarnitine (8.32 × 10^{-10})	NA
ASGA0083304	rs81336779	1	77011485	NA	NA	68186587	NA	NA	2	NA	Isovalerylcarnitine (2.79 × 10^{-8}), Propionylcarnitine (8.32 × 10^{-10})	NA
DRGA0000994	NA	1	66455394	NA	NA	NA	NA	NA	2	NA	Isovalerylcarnitine (2.79 × 10^{-8}), Propionylcarnitine (8.32 × 10^{-10})	NA
DRGA0001072	NA	1	70784564	NA	NA	NA	NA	NA	2	NA	Isovalerylcarnitine (2.79 × 10^{-8}), Propionylcarnitine (8.32 × 10^{-10})	NA
DRGA0001073	NA	1	70898747	NA	NA	NA	NA	NA	2	NA	Isovalerylcarnitine (2.79 × 10^{-8}), Propionylcarnitine (8.32 × 10^{-10})	NA
H3GA0001865	rs80798325	1	67809463	NA	NA	60549619	NA	NA	2	NA	Isovalerylcarnitine (2.79 × 10^{-8}), Propionylcarni	NA

											tine (8.32 × 10^{-10})	
H3GA0001937	rs81354639	1	75626511	*MCHR2* (NM_001044609)	43991	67072857	*SIM1* (NM_001172585)	146052	2	NA	Isovalerylcarnitine (2.79 × 10^{-8}), Propionylcarnitine (8.32 × 10^{-10})	NA
H3GA0001949	rs81354656	1	76297353	*SIM1* (NM_001172585)	226123	67718944	*SIM1* (NM_001172585)	425808	2	NA	Isovalerylcarnitine (2.79 × 10^{-8}), Propionylcarnitine (8.32 × 10^{-10})	NA
H3GA0001956	rs80918218	1	77402476	NA	NA	68525440	NA	NA	2	NA	Isovalerylcarnitine (2.79 × 10^{-8}), Propionylcarnitine (8.32 × 10^{-10})	NA
H3GA0001966	rs81354723	1	78423848	NA	NA	69453835	NA	NA	2	NA	Isovalerylcarnitine (2.79 × 10^{-8}), Propionylcarnitine (8.32 × 10^{-10})	NA
H3GA0014734	rs80852914	4	135234038	*DNTTIP2* (NM_001243674)	180160	123595435	*DNTTIP2* (NM_001243674)	186975	2	NA	Glutamic acid (3.57 × 10^{-10})	Glutamic acid (4.80 × 10^{-8})
H3GA0046845	rs81460781	16	65033887	*MAT2B* (NM_001142832)	62817	60196022	*MAT2B* (NM_001142832)	59524	2	NA	Isovalerylcarnitine (2.79 × 10^{-8}), Propionylcarnitine (8.32 × 10^{-10})	NA
INRA0002726	rs341934411	1	74096746	*FBXL4* (NM_001171752)	325687	65537992	*FBXL4* (NM_001171752)	334180	2	NA	Isovalerylcarnitine (2.79 × 10^{-8}), Propionylcarnitine (8.32 × 10^{-10})	NA
INRA0002819	rs338023709	1	77424826	NA	NA	68549249	NA	NA	2	NA	Isovalerylcarnitine (2.79 ×	NA

											10^{-8}), Propionylcarnitine (8.32 × 10^{-10})	
INRA0002820	rs331278924	1	77457737	NA	NA	68581700	NA	NA	2	NA	Isovalerylcarnitine (2.79 × 10^{-8}), Propionylcarnitine (8.32 × 10^{-10})	NA
INRA0002823	rs319323532	1	77616272	NA	NA	68740759	NA	NA	2	NA	Isovalerylcarnitine (2.79 × 10^{-8}), Propionylcarnitine (8.32 × 10^{-10})	NA
MARC0021047	rs80872183	1	78061517	NA	NA	68995266	NA	NA	2	NA	Isovalerylcarnitine (2.79 × 10^{-8}), Propionylcarnitine (8.32 × 10^{-10})	NA
MARC0027518	rs81222968	1	64488649	*RRAGD* (NM_001243623)	289310	57586607	*RRAGD* (NM_001243623)	162549	2	NA	Isovalerylcarnitine (2.79 × 10^{-8}), Propionylcarnitine (8.32 × 10^{-10})	NA
MARC0034307	rs81228351	1	76929255	NA	NA	68103552	NA	NA	2	NA	Isovalerylcarnitine (2.79 × 10^{-8}), Propionylcarnitine (8.32 × 10^{-10})	NA
MARC0050325	rs80884859	1	76254021	*SIM1* (NM_001172585)	182791	67496805	*SIM1* (NM_001172585)	203669	2	NA	Isovalerylcarnitine (2.79 × 10^{-8}), Propionylcarnitine (8.32 × 10^{-10})	NA
MARC0059407	rs80976465	1	72300056	*FHL5* (NM_001243314)	179132	63930516	*FHL5* (NM_001243314)	178366	2	NA	Isovalerylcarnitine (2.79 × 10^{-8}),	NA

											Propionylcarnitine (8.32 × 10^{-10})	
MARC0063106	rs81251885	1	67392874	NA	NA	60190732	NA	NA	2	NA	Isovalerylcarnitine (2.79 × 10^{-8}), Propionylcarnitine (8.32 × 10^{-10})	NA
MARC0068954	rs80860397	1	69079272	NA	NA	61286411	NA	NA	2	NA	Isovalerylcarnitine (2.79 × 10^{-8}), Propionylcarnitine (8.32 × 10^{-10})	NA
MARC0075306	rs80838693	1	74264584	*FBXL4* (NM_001171752)	157849	65711728	*FBXL4* (NM_001171752)	160444	2	NA	Isovalerylcarnitine (2.79 × 10^{-8}), Propionylcarnitine (8.32 × 10^{-10})	NA
MARC0075913	rs81260849	10	6389965	NA	NA	4673147	NA	NA	2	NA	Carnitine (1.24 × 10^{-8})	Carnitine (5.24 × 10^{-8})
MARC0080116	rs80801724	4	3550229	NA	NA	3937200	NA	NA	2	Pyruvic acid (4.06 × 10^{-8})	NA	Citrulline (8.58 × 10^{-7})
MARC0085569	rs80948741	5	68417760	*CCND2* (NM_214088)	85816	66145175	*CCND2* (NM_214088)	30600	2	NA	Glutamic acid (3.57 × 10^{-10})	Glutamic acid (4.80 × 10^{-8})
SIRI0000655	rs318393313	1	78666093	NA	NA	69661389	NA	NA	2	NA	Isovalerylcarnitine (2.79 × 10^{-8}), Propionylcarnitine (8.32 × 10^{-10})	NA
WU_10.2_18_15516325	rs339631616	18	15516325	*AKR1B1* (NM_001001539)	80480	14653756	*AKR1B1* (NM_001001539)	80335	2	NA	Carnitine (1.24 × 10^{-8})	Carnitine (5.24 × 10^{-8})
WU_10.2_9_136780050	rs322630479	9	136780050	*LAMC1* (NM_001271715)	111754	124468420	*LAMC1* (NM_001271715)	62453	2	NA	Carnitine (1.24 × 10^{-8})	Carnitine (5.24 × 10^{-8})
ALGA0038416	rs80964126	7	8540896	NA	NA	8068810	*MIR7136* (NR_128452)	66398	3	Isovalerylcarnitine (3.47 × 10^{-8}), Propionylcarni	NA	Propionylcarnitine (4.66 × 10^{-7})

										tine (1.17 × 10^{-9})		
ALGA0081238	rs80954044	14	124416261	*STN1* (NM_001243685)	310170	114534775	*MIR1307* (NR_035394)	209072	3	Isovalerylcarnitine (1.27 × 10^{-8}), Propionylcarnitine (3.01 × 10^{-10})	NA	Propionylcarnitine (3.27 × 10^{-8})
ASGA0093565	rs81312507	6	135424176	*DNAJC6* (NM_001145378)	8th intron	147018965	*DNAJC6* (NM_001145378)	8th intron	3	1-hexadecyl-sn-glycero-3-phosphocholine (2.78 × 10^{-9}), 1-myristoyl-sn-glycero-3-phosphocholine (1.35 × 10^{-8}), LysoPC(16:0) (1.22 × 10^{-7})	NA	NA
DRGA0014486	NA	14	121391605	*KAZALD1* (NM_001244551)	312903	NA	NA	NA	3	Isovalerylcarnitine (1.27 × 10^{-8}), Propionylcarnitine (3.01 × 10^{-10})	NA	Propionylcarnitine (3.27 × 10^{-8})
H3GA0053559	rs81344179	17	46932020	*LBP* (NM_001128435)	63443	41492905	*LBP* (NM_001128435)	63438	3	1-hexadecyl-sn-glycero-3-phosphocholine (3.05 × 10^{-10}), 1-myristoyl-sn-glycero-3-phosphocholine (2.76 × 10^{-8}), LysoPC(16:0) (4.69 × 10^{-9})	NA	NA
M1GA0016778	rs81435383	12	44192225	*CDK5R1* (NM_001101816)	60431	42432989	*CDK5R1* (NM_001101816)	60402	3	Pyruvic acid (1.30 × 10^{-9})	NA	Citrulline (3.94 × 10^{-7}), Pyruvic acid (1.57 × 10^{-8})
WU_10.2_14_132246191	rs339220618	14	132246191	*ADRA2A* (NM_214400)	157702	121319314	*ADRA2A* (NM_214400)	150257	3	Isovalerylcarnitine (1.27 × 10^{-8}), Propionylcarni	NA	Propionylcarnitine (3.27 × 10^{-8})

										tine (3.01 × 10^{-10})		
WU_10.2_6_136216429	rs343240155	6	136216429	*JAK1* (NM_214114)	298537	147890682	*JAK1* (NM_214114)	323495	3	1-hexadecyl-sn-glycero-3-phosphocholine (2.78 × 10^{-9}), 1-myristoyl-sn-glycero-3-phosphocholine (1.35 × 10^{-8}), LysoPC(16:0) (1.22 × 10^{-7})	NA	NA
WU_10.2_6_136863547	rs338771482	6	136863547	*PGM1* (NM_001246318)	307725	148410937	*PGM1* (NM_001246318)	440248	3	1-hexadecyl-sn-glycero-3-phosphocholine (2.78 × 10^{-9}), 1-myristoyl-sn-glycero-3-phosphocholine (1.35 × 10^{-8}), LysoPC(16:0) (1.22 × 10^{-7})	NA	NA
WU_10.2_6_136876717	rs323124581	6	136876717	*PGM1* (NM_001246318)	294555	148424103	*PGM1* (NM_001246318)	294555	3	1-hexadecyl-sn-glycero-3-phosphocholine (2.78 × 10^{-9}), 1-myristoyl-sn-glycero-3-phosphocholine (1.35 × 10^{-8}), LysoPC(16:0) (1.22 × 10^{-7})	NA	NA
WU_10.2_6_136972846	rs322169260	6	136972846	*PGM1* (NM_001246318)	198426	148652784	*PGM1* (NM_001246318)	198401	3	1-hexadecyl-sn-glycero-3-phosphocholine (2.78 × 10^{-9}), 1-myristoyl-sn-glycero-3-phosphocholine (1.35 × 10^{-8}), LysoPC(16:0) (1.22 × 10^{-7})	NA	NA

ALGA0099866	rs80900934	X	105147853	*ACSL4* (NM_001038694)	228041	89533931	*ACSL4* (NM_001038694)	226931	4	1-hexadecyl-sn-glycero-3-phosphocholine (3.35×10^{-10}), 1-myristoyl-sn-glycero-3-phosphocholine (2.77×10^{-9}), LysoPC(16:0) (1.16×10^{-7}), Pyruvic acid (8.03×10^{-9})	NA	NA
ASGA0018324	rs80893920	4	12174119	NA	NA	11908832	NA	NA	4	Citrulline (2.75×10^{-9}), Pyruvic acid (1.24×10^{-16})	NA	Citrulline (4.22×10^{-11}), Pyruvic acid (1.64×10^{-13})
ASGA0081223	rs81473421	X	103657659	NA	NA	89131099	NA	NA	4	1-hexadecyl-sn-glycero-3-phosphocholine (2.63×10^{-15}), 1-myristoyl-sn-glycero-3-phosphocholine (1.49×10^{-13}), LysoPC(16:0) (7.03×10^{-11})	NA	1-hexadecyl-sn-glycero-3-phosphocholine (4.05×10^{-9})
INRA0003881	rs335367606	1	126310419	*AQP9* (NM_001112684)	357302	114183384	*AQP9* (NM_001112684)	334932	4	1-hexadecyl-sn-glycero-3-phosphocholine (2.63×10^{-15}), 1-myristoyl-sn-glycero-3-phosphocholine (1.49×10^{-13}), LysoPC(16:0) (7.03×10^{-11})	NA	1-hexadecyl-sn-glycero-3-phosphocholine (4.05×10^{-9})
MARC0046138	rs81238314	1	228287595	NA	NA	204652721	NA	NA	4	1-hexadecyl-sn-glycero-3-phosphocholine (2.63×10^{-15}), 1-myristoyl-sn-glycero-3-phosphocholin	NA	1-hexadecyl-sn-glycero-3-phosphocholine (4.05×10^{-9})

										e (1.49×10^{-13}), LysoPC(16:0) (7.03×10^{-11})		
WU_10.2_X_103597980	rs337506275	X	103597980	NA	NA	89142300	NA	NA	4	1-hexadecyl-sn-glycero-3-phosphocholine (2.63×10^{-15}), 1-myristoyl-sn-glycero-3-phosphocholine (1.49×10^{-13}), LysoPC(16:0) (7.03×10^{-11})	NA	1-hexadecyl-sn-glycero-3-phosphocholine (4.05×10^{-9})
WU_10.2_X_103653646	rs331101550	X	103653646	NA	NA	89135113	NA	NA	4	1-hexadecyl-sn-glycero-3-phosphocholine (2.63×10^{-15}), 1-myristoyl-sn-glycero-3-phosphocholine (1.49×10^{-13}), LysoPC(16:0) (7.03×10^{-11})	NA	1-hexadecyl-sn-glycero-3-phosphocholine (4.05×10^{-9})
WU_10.2_X_104796075	rs336044303	X	104796075	NA	NA	89243691	NA	NA	4	1-hexadecyl-sn-glycero-3-phosphocholine (2.63×10^{-15}), 1-myristoyl-sn-glycero-3-phosphocholine (1.49×10^{-13}), LysoPC(16:0) (7.03×10^{-11})	NA	1-hexadecyl-sn-glycero-3-phosphocholine (4.05×10^{-9})
WU_10.2_X_104910069	rs324354886	X	104910069	*ACSL4* (NM_001038694)	465825	89357685	*ACSL4* (NM_001038694)	403177	4	1-hexadecyl-sn-glycero-3-phosphocholine (2.63×10^{-15}), 1-myristoyl-sn-glycero-3-phosphocholine (1.49×10^{-13}), LysoPC(16:0) (7.03×10^{-11})	NA	1-hexadecyl-sn-glycero-3-phosphocholine (4.05×10^{-9})

WU_10.2_X_104956283	rs321020009	X	104956283	*ACSL4* (NM_001038694)	419611	89403886	*ACSL4* (NM_001038694)	356976	4	1-hexadecyl-sn-glycero-3-phosphocholine (2.63×10^{-15}), 1-myristoyl-sn-glycero-3-phosphocholine (1.49×10^{-13}), LysoPC(16:0) (7.03×10^{-11})	NA	1-hexadecyl-sn-glycero-3-phosphocholine (4.05×10^{-9})
WU_10.2_X_104980830	rs343822051	X	104980830	*ACSL4* (NM_001038694)	395064	89428431	*ACSL4* (NM_001038694)	332431	4	1-hexadecyl-sn-glycero-3-phosphocholine (2.63×10^{-15}), 1-myristoyl-sn-glycero-3-phosphocholine (1.49×10^{-13}), LysoPC(16:0) (7.03×10^{-11})	NA	1-hexadecyl-sn-glycero-3-phosphocholine (4.05×10^{-9})
WU_10.2_X_105559450	rs339642275	X	105559450	*ACSL4* (NM_001038694)	106806	89892408	*ACSL4* (NM_001038694)	54800	4	1-hexadecyl-sn-glycero-3-phosphocholine (3.35×10^{-10}), 1-myristoyl-sn-glycero-3-phosphocholine (2.77×10^{-9}), LysoPC(16:0) (1.16×10^{-7}), Pyruvic acid (8.03×10^{-9})	NA	NA
WU_10.2_X_105583738	rs326175201	X	105583738	*ACSL4* (NM_001038694)	131094	89916695	*ACSL4* (NM_001038694)	79087	4	1-hexadecyl-sn-glycero-3-phosphocholine (2.63×10^{-15}), 1-myristoyl-sn-glycero-3-phosphocholine (1.49×10^{-13}), LysoPC(16:0) (7.03×10^{-11})	NA	1-hexadecyl-sn-glycero-3-phosphocholine (4.05×10^{-9})

WU_10.2_X_114649203	rs331077355	X	114649203	*IL13RA1* (NM_214341)	262774	96863521	*IL13RA1* (NM_214341)	437438	4	Citrulline (2.75 × 10^{-9}), Pyruvic acid (1.24 × 10^{-16})	NA	Citrulline (4.22 × 10^{-11}), Pyruvic acid (1.64 × 10^{-13})

Note: NA indicates not applicable. [#]indicates the SNP position and annotation of latest porcine RefSeq database (Sscrofa11.1/susScr11).

Supplementary Table S2. All the metabolites in association with significant SNPs from first, second, and combined two sampling times.

Metabolite	First Sampling Time		Second Sampling Time		Combined Two Sampling Times		Common Significant SNP
	Significant SNP Number	Significant SNP Name	Significant SNP Number	Significant SNP Name	Significant SNP Number	Significant SNP Name	
1-hexadecyl-sn-glycero-3-phosphocholine	23	INRA0003881, MARC0046138, WU_10.2_6_135312468, ASGA0093565, WU_10.2_6_136216429, WU_10.2_6_136863547, WU_10.2_6_136876717, WU_10.2_6_136972846, MARC0060063, WU_10.2_8_142992366, DRGA0008955, ASGA0040302, H3GA0053559, WU_10.2_X_103597980, WU_10.2_X_103653646, ASGA0081223, WU_10.2_X_104796075, WU_10.2_X_104910069, WU_10.2_X_104956283, WU_10.2_X_104980830, ALGA0099866, WU_10.2_X_105559450, WU_10.2_X_105583738	1	WU_10.2_X_37114281	10	INRA0003881, MARC0046138, WU_10.2_X_103597980, WU_10.2_X_103653646, ASGA0081223, WU_10.2_X_104796075, WU_10.2_X_104910069, WU_10.2_X_104956283, WU_10.2_X_104980830, WU_10.2_X_105583738	0
1-myristoyl-sn-glycero-3-phosphocholine	18	INRA0003881, MARC0046138, ASGA0093565, WU_10.2_6_136216429, WU_10.2_6_136863547, WU_10.2_6_136876717, WU_10.2_6_136972846, H3GA0053559, WU_10.2_X_103597980, WU_10.2_X_103653646, ASGA0081223, WU_10.2_X_104796075, WU_10.2_X_104910069, WU_10.2_X_104956283, WU_10.2_X_104980830, ALGA0099866,	2	H3GA0015652, MARC0010841	0	0	0

		WU_10.2_X_105559450, WU_10.2_X_105583738					
Aspartic acid	0	0	4	3_118102178, H3GA0055935, ALGA0061605, 18_2566820	0	0	0
Carnitine	0	0	5	ASGA0044546, WU_10.2_9_136780050, MARC0075913, MARC0083382, WU_10.2_18_15516325	4	ASGA0044546, WU_10.2_9_136780050, MARC0075913, WU_10.2_18_15516325	0
Citrulline	2	ASGA0018324, WU_10.2_X_114649203	0	0	5	MARC0080116, ASGA0018324, MARC0040505, M1GA0016778, WU_10.2_X_114649203	0
Cotinine	2	M1GA0015335, ALGA0072779	0	0	0	0	0
Creatinine	1	ASGA0083287	0	0	0	0	0
Glutamic acid	0	0	5	ALGA0028664, H3GA0014734, ASGA0023013, ASGA0025965, MARC0085569	5	ALGA0028664, H3GA0014734, ASGA0023013, ASGA0025965, MARC0085569	0
Guanine	0	0	5	WU_10.2_12_49599992, ASGA0054868, WU_10.2_12_49979240, ALGA0083708, ALGA0104701	0	0	0
Isoleucyl proline	0	0	11	ALGA0000375, ALGA0043837, WU_10.2_7_100175430, ALGA0046501, WU_10.2_8_12998407, ALGA0046512, WU_10.2_9_128572226, H3GA0028115, WU_10.2_13_204594763, WU_10.2_13_204608919, WU_10.2_13_204631833	0	0	0
Isovalerylcarnitine	10	H3GA0007606, M1GA0003090, WU_10.2_2_132463311, WU_10.2_2_132503341, WU_10.2_2_132570224, ALGA0038416, DRGA0014486, ALGA0081238, WU_10.2_14_132246191, ALGA0103368	57	MARC0027518, DRGA0000994, MARC0063106, H3GA0001865, MARC0068954, ASGA0003182, ALGA0003891, ALGA0003900, DRGA0001072, DRGA0001073, ASGA0003194, MARC0059407, ALGA0003935, ALGA0003952, ALGA0003953, ASGA0003235, INRA0002726, MARC0075306, ALGA0003995, ALGA0004000,	1	ALGA0103368	0

				ALGA0004002, ALGA0004005, ALGA0004006, ALGA0004024, ALGA0004041, ALGA0004042, ALGA0004046, ALGA0004048, ASGA0003288, H3GA0001937, ALGA0004073, ALGA0004090, ASGA0003312, ASGA0003314, MARC0050325, H3GA0001949, ALGA0004093, ASGA0003317, ASGA0003315, MARC0034307, ASGA0083304, ALGA0004143, H3GA0001956, INRA0002819, INRA0002820, ALGA0004148, INRA0002823, ALGA0004169, MARC0021047, ALGA0004173, ASGA0003333, ALGA0004177, H3GA0001966, ASGA0003335, SIRI0000655, ASGA0057312, H3GA0046845			
Lactic acid	0	0	1	WU_10.2_X_104031384	0	0	0
LysoPC(16:0)	21	INRA0003881, MARC0046138, ASGA0093565, WU_10.2_6_136216429, WU_10.2_6_136863547, WU_10.2_6_136876717, WU_10.2_6_136972846, H3GA0053559, WU_10.2_17_48295280, ASGA0094610, MARC0027232, WU_10.2_X_103597980, WU_10.2_X_103653646, ASGA0081223, WU_10.2_X_104796075, WU_10.2_X_104910069, WU_10.2_X_104956283, WU_10.2_X_104980830, ALGA0099866, WU_10.2_X_105559450, WU_10.2_X_105583738	0	0	0	0	0
Monosaccharide	0	0	0	0	1	ASGA0095299	0
Nicotine amide	0	0	1	2_29041727	0	0	0

Propionylcarnitine	4	ALGA0038416, DRGA0014486, ALGA0081238, WU_10.2_14_132246191	61	MARC0027518, DRGA0000994, MARC0063106, H3GA0001865, MARC0068954, ASGA0003182, ALGA0003891, ALGA0003900, DRGA0001072, DRGA0001073, ASGA0003194, MARC0059407, ALGA0003935, ALGA0003952, ALGA0003953, ASGA0003235, INRA0002726, MARC0075306, ALGA0003995, ALGA0004000, ALGA0004002, ALGA0004005, ALGA0004006, ALGA0004024, ALGA0004041, ALGA0004042, ALGA0004046, ALGA0004048, ASGA0003288, H3GA0001937, ALGA0004073, ALGA0004090, ASGA0003312, ASGA0003314, MARC0050325, H3GA0001949, ALGA0004093, ASGA0003317, ASGA0003315, MARC0034307, ASGA0083304, ALGA0004143, H3GA0001956, INRA0002819, INRA0002820, ALGA0004148, INRA0002823, ALGA0004169, MARC0021047, ALGA0004173, ASGA0003333, ALGA0004177, H3GA0001966, ASGA0003335, SIRI0000655, DRGA0004012, WU_10.2_10_71866847, H3GA0035928, ASGA0057312, H3GA0046845, ASGA0078611	4	ALGA0038416, DRGA0014486, ALGA0081238, WU_10.2_14_132246191
Pyruvic acid	16	MARC0110390, ALGA0014130, MARC0080116, ASGA0018324, ALGA0049375, ALGA0049385, MARC0001720, ASGA0042433, H3GA0026947, ASGA0042436, MARC0092124, INRA0031485, M1GA0016778, ALGA0099866, WU_10.2_X_105559450, WU_10.2_X_114649203	1	MARC0023109	3	ASGA0018324, M1GA0016778, WU_10.2_X_114649203
Total	97		154		33	0

Supplementary Table S3. Top ten SNPs associated with residual feed intake (RFI).

SNP Name	Chromosome	Position	*p*-Value	Nearest Gene	Gene Description	Distance
DRGA0008061	7	110,711,289	2.09×10^{-4}	NA	NA	NA
WU_10.2_3_2710244	3	2,710,244	2.76×10^{-4}	*GRIFIN* (NM_001145219)	Galectin-related inter-fiber protein	458,576
ALGA0038510	7	9,882,571	2.83×10^{-4}	*SIRT5* (NM_001105308)	Sirtuin 5	445,152
WU_10.2_3_13191193	3	13,191,193	3.18×10^{-4}	*AUTS2* (NM_001246269)	Activator of transcription and developmental regulator AUTS2	417,999
WU_10.2_3_13390270	3	13,390,270	3.18×10^{-4}	*AUTS2* (NM_001246269)	Activator of transcription and developmental regulator AUTS2	218,922
H3GA0005011	1	301,750,612	3.31×10^{-4}	*ANGPTL2* (NM_001109946)	Angiopoietin like 2	175,995
M1GA0001651	1	301,911,374	3.31×10^{-4}	*PTRH1* (NM_001258431)	Peptidyl-tRNA hydrolase 1 homolog	194,106
WU_10.2_1_302080876	1	302,080,876	3.31×10^{-4}	*PTRH1* (NM_001258431)	Peptidyl-tRNA hydrolase 1 homolog	24,604
ALGA0010572	1	302,086,585	3.31×10^{-4}	*PTRH1* (NM_001258431)	Peptidyl-tRNA hydrolase 1 homolog	18,895
ASGA0035769	7	108,716,194	3.33×10^{-4}	NA	NA	NA

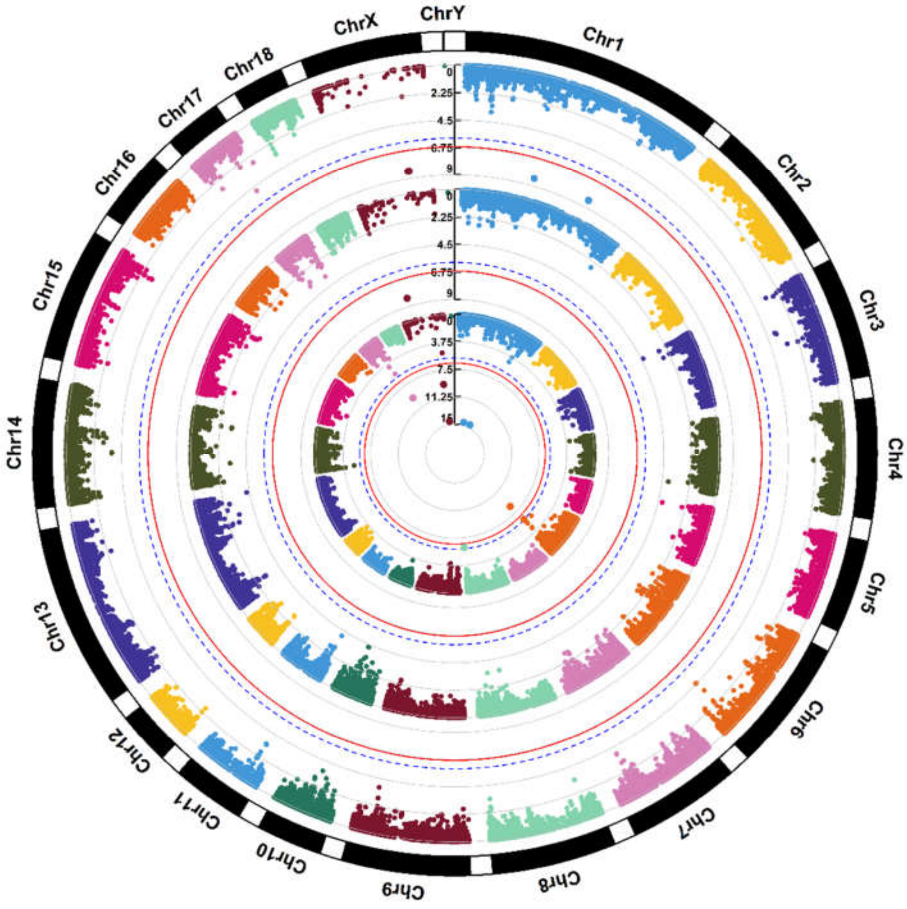

Supplementary Figure S1.1. Manhattan plots of genome-wide association for 1-hexadecyl-sn-glycero-3-phosphocholine (M1). Note: Y-axis indicates the $\log_{10}$(p-value). Blue dotted and red solid lines indicate the genome-wide threshold of 0.05 and 0.01 after Bonferroni multiple testing, respectively. The three tracks indicate the metabolites from first sampling time, second sampling time, and combined two sampling times from outside to inside.

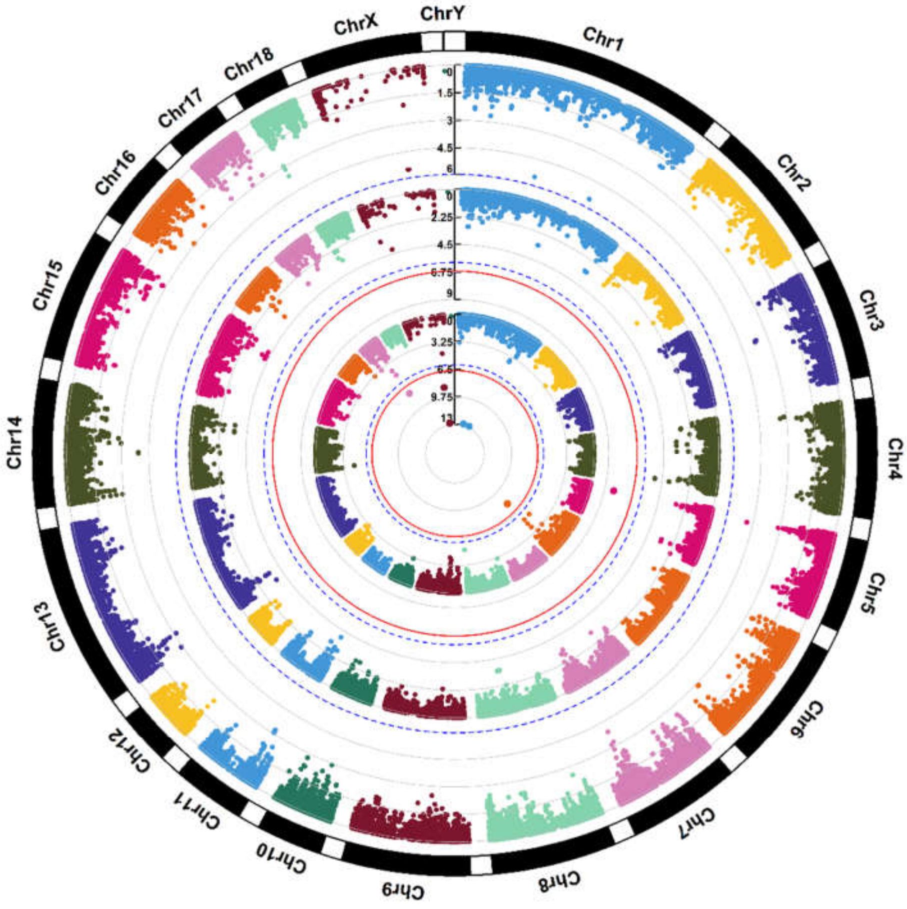

Supplementary Figure S1.2. Manhattan plots of genome-wide association for 1-myristoyl-sn-glycero-3-phosphocholine (M2). Note: Y-axis indicates the $\log_{10}(p\text{-value})$. Blue dotted and red solid lines indicate the genome-wide threshold of 0.05 and 0.01 after Bonferroni multiple testing, respectively. The three tracks indicate the metabolites from first sampling time, second sampling time, and combined two sampling times from outside to inside.

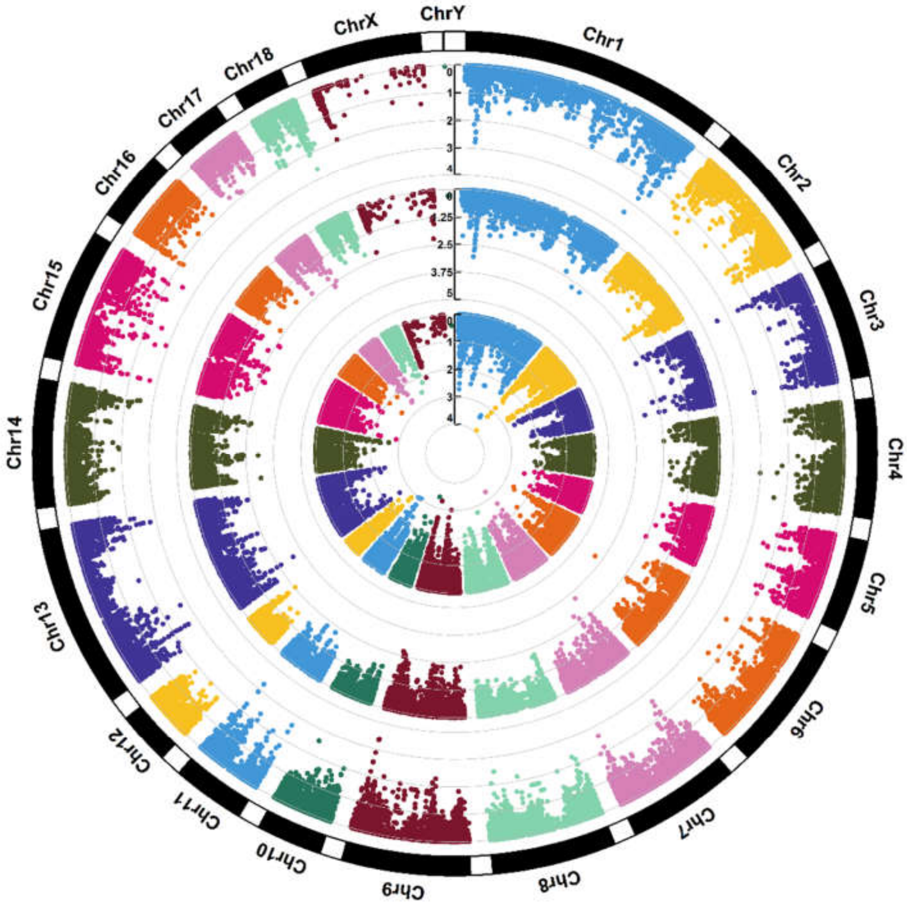

Supplementary Figure S1.3. Manhattan plots of genome-wide association for trimethylammonium (M3). Note: Y-axis indicates the $\log_{10}$(*p*-value). Blue dotted and red solid lines indicate the genome-wide threshold of 0.05 and 0.01 after Bonferroni multiple testing, respectively. The three tracks indicate the metabolites from first sampling time, second sampling time, and combined two sampling times from outside to inside.

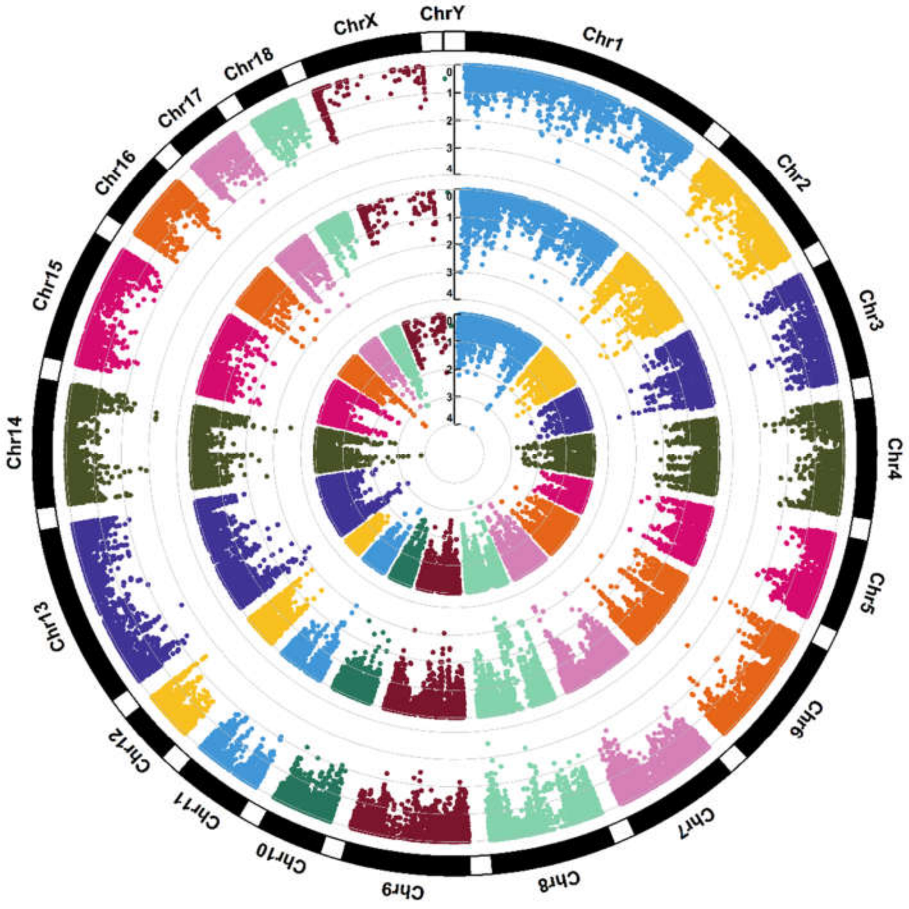

Supplementary Figure S1.4. Manhattan plots of genome-wide association for 4-aminobenzoic acid (M4). Note: Y-axis indicates the $\log_{10}$(*p*-value). Blue dotted and red solid lines indicate the genome-wide threshold of 0.05 and 0.01 after Bonferroni multiple testing, respectively. The three tracks indicate the metabolites from first sampling time, second sampling time, and combined two sampling times from outside to inside.

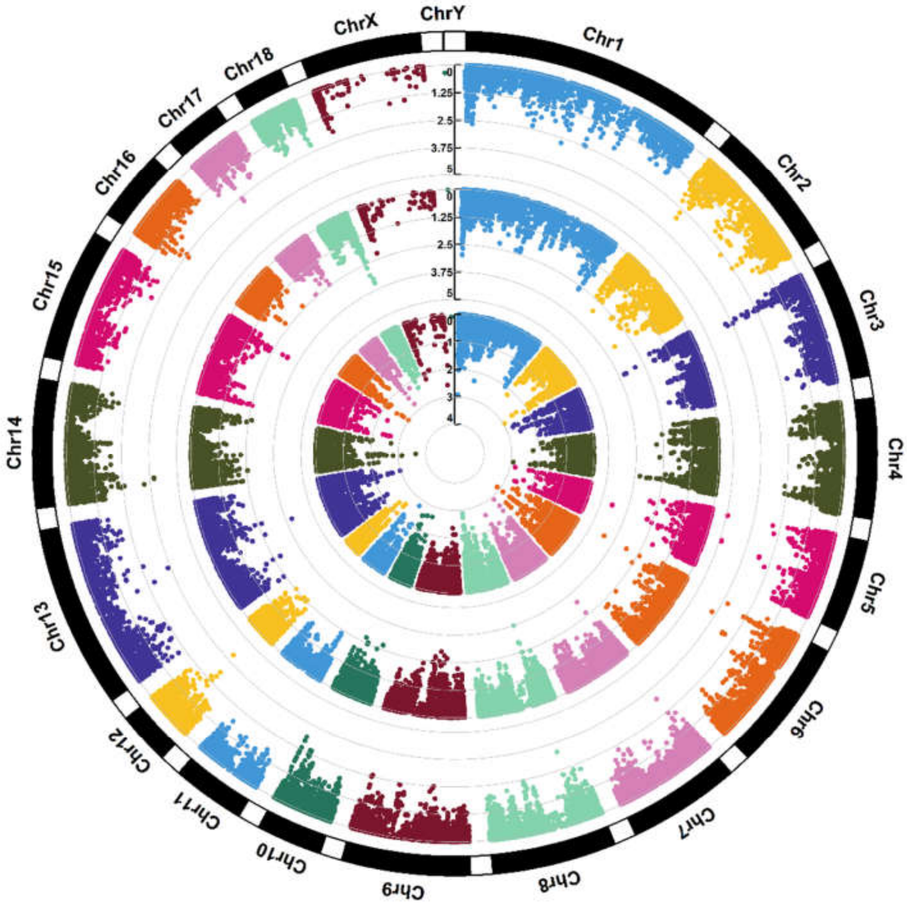

Supplementary Figure S1.5. Manhattan plots of genome-wide association for 5-methyl-5,6-dihydrouracils (M5). Note: Y-axis indicates the $\log_{10}$(p-value). Blue dotted and red solid lines indicate the genome-wide threshold of 0.05 and 0.01 after Bonferroni multiple testing, respectively. The three tracks indicate the metabolites from first sampling time, second sampling time, and combined two sampling times from outside to inside.

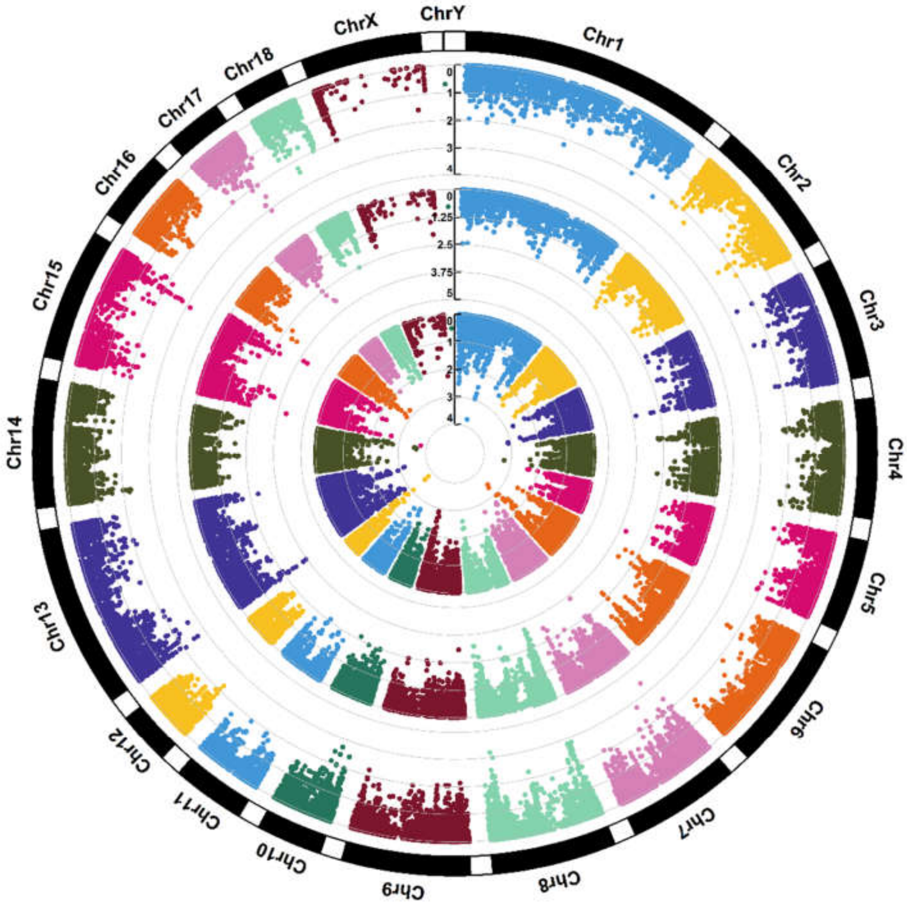

Supplementary Figure S1.6. Manhattan plots of genome-wide association for acetaminophen (M6). Note: Y-axis indicates the $\log_{10}$(*p*-value). Blue dotted and red solid lines indicate the genome-wide threshold of 0.05 and 0.01 after Bonferroni multiple testing, respectively. The three tracks indicate the metabolites from first sampling time, second sampling time, and combined two sampling times from outside to inside.

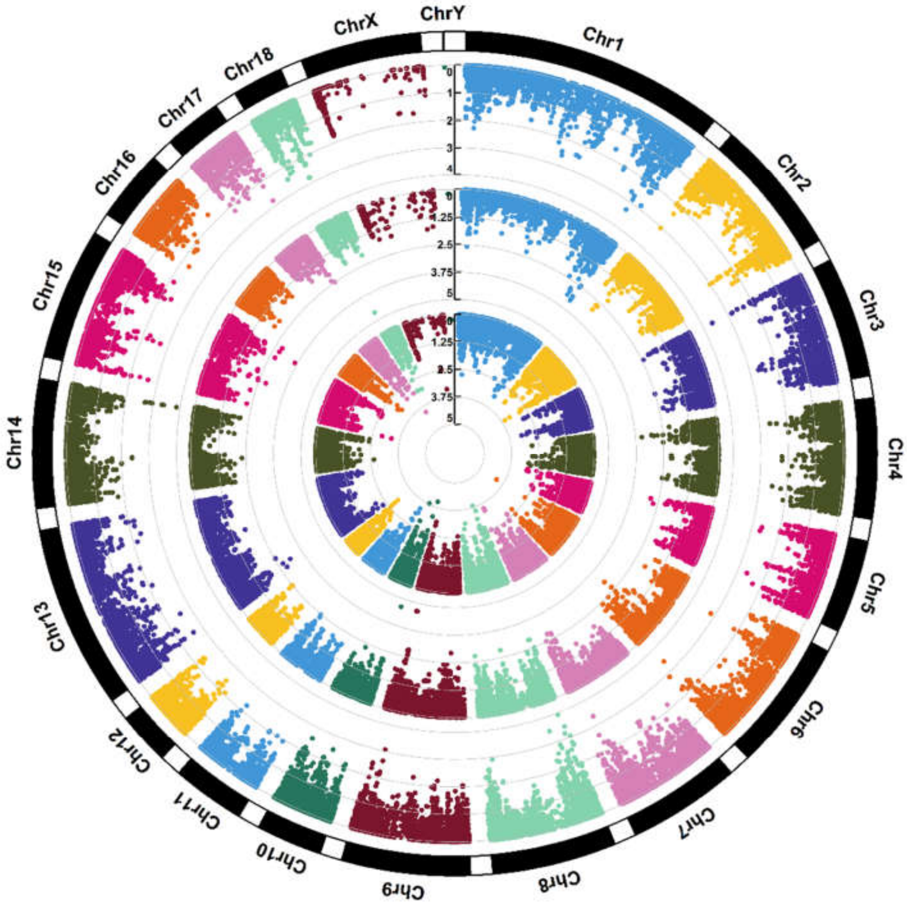

Supplementary Figure S1.7. Manhattan plots of genome-wide association for acetylcarnitine (M7). Note: Y-axis indicates the $\log_{10}$(p-value). Blue dotted and red solid lines indicate the genome-wide threshold of 0.05 and 0.01 after Bonferroni multiple testing, respectively. The three tracks indicate the metabolites from first sampling time, second sampling time, and combined two sampling times from outside to inside.

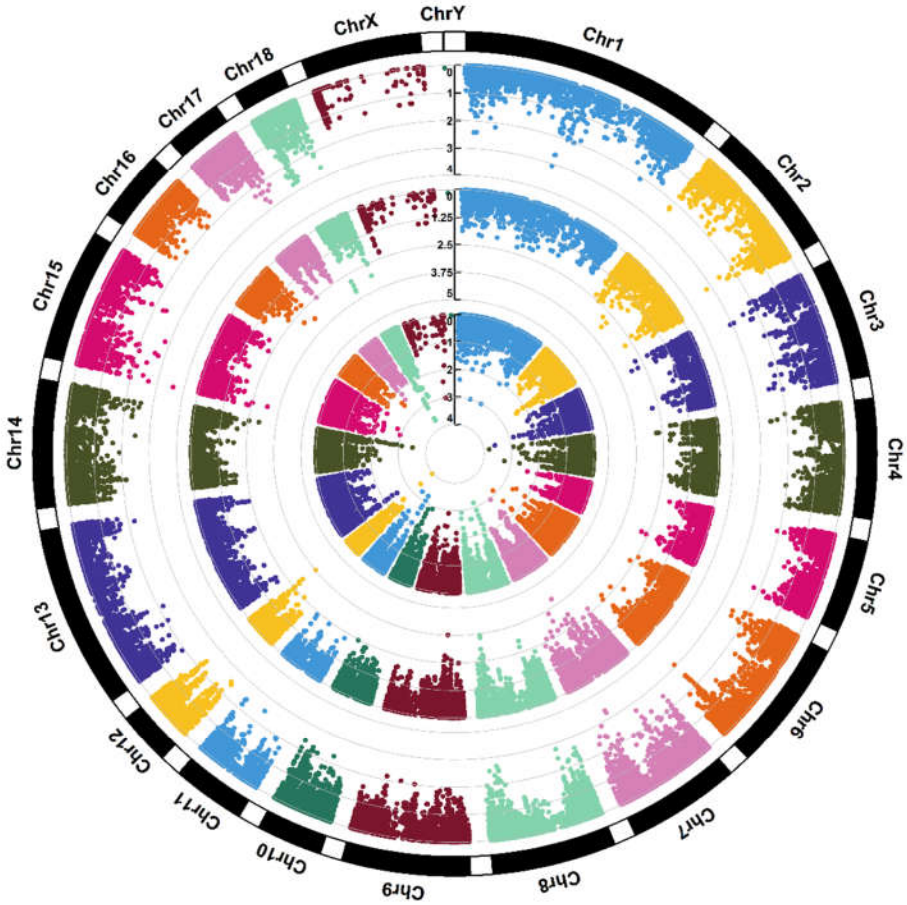

.

Supplementary Figure S1.8. Manhattan plots of genome-wide association for alanine (M8). Note: Y-axis indicates the $\log_{10}$(*p*-value). Blue dotted and red solid lines indicate the genome-wide threshold of 0.05 and 0.01 after Bonferroni multiple testing, respectively. The three tracks indicate the metabolites from first sampling time, second sampling time, and combined two sampling times from outside to inside.

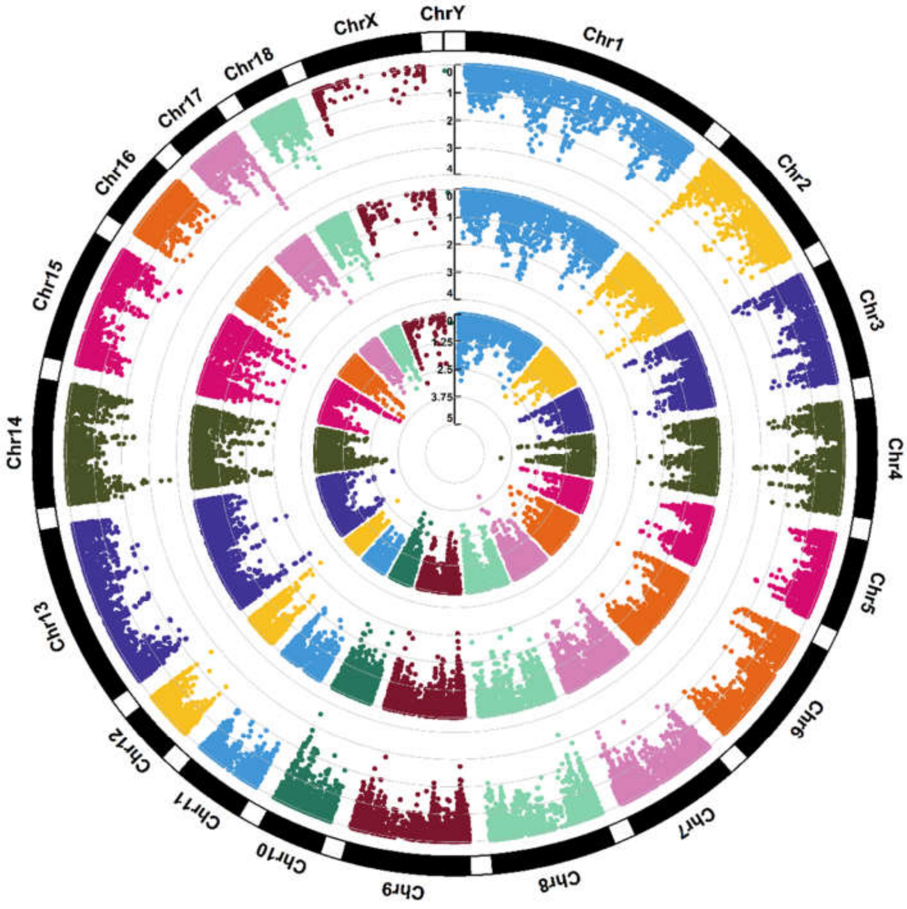

Supplementary Figure S1.9. Manhattan plots of genome-wide association for arginine (M9). Note: Y-axis indicates the $\log_{10}$(*p*-value). Blue dotted and red solid lines indicate the genome-wide threshold of 0.05 and 0.01 after Bonferroni multiple testing, respectively. The three tracks indicate the metabolites from first sampling time, second sampling time, and combined two sampling times from outside to inside.

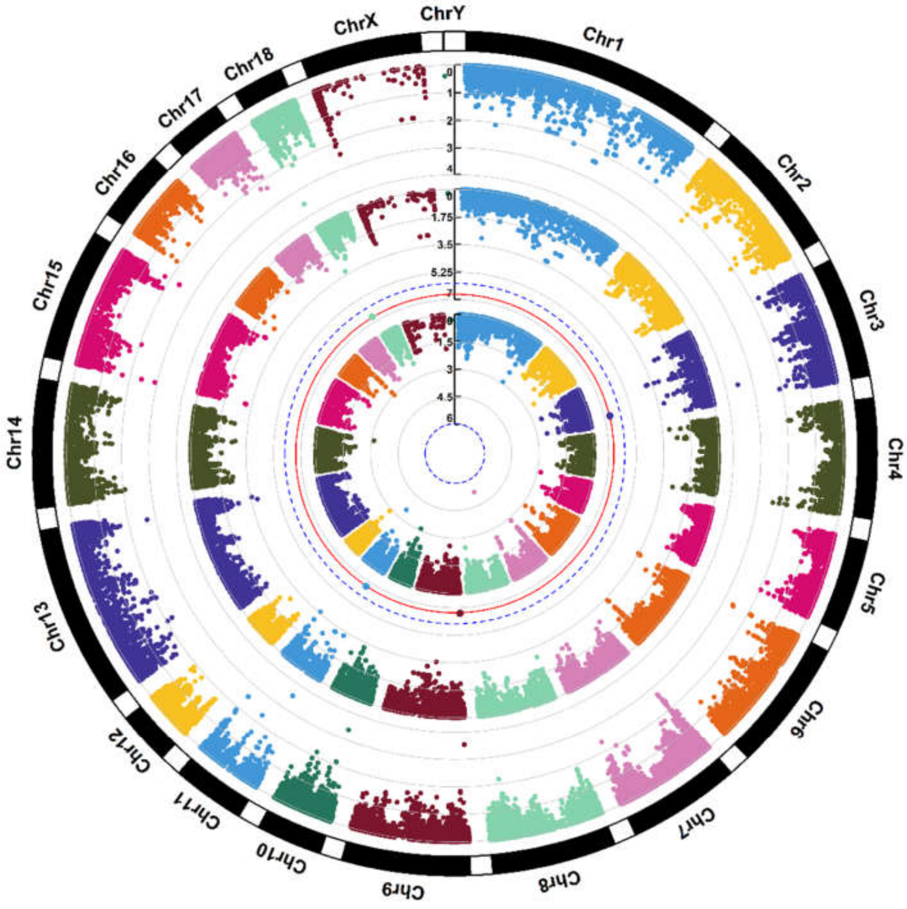

Supplementary Figure S1.10. Manhattan plots of genome-wide association for aspartic acid (M10). Note: Y-axis indicates the $\log_{10}$(*p*-value). Blue dotted and red solid lines indicate the genome-wide threshold of 0.05 and 0.01 after Bonferroni multiple testing, respectively. The three tracks indicate the metabolites from first sampling time, second sampling time, and combined two sampling times from outside to inside.

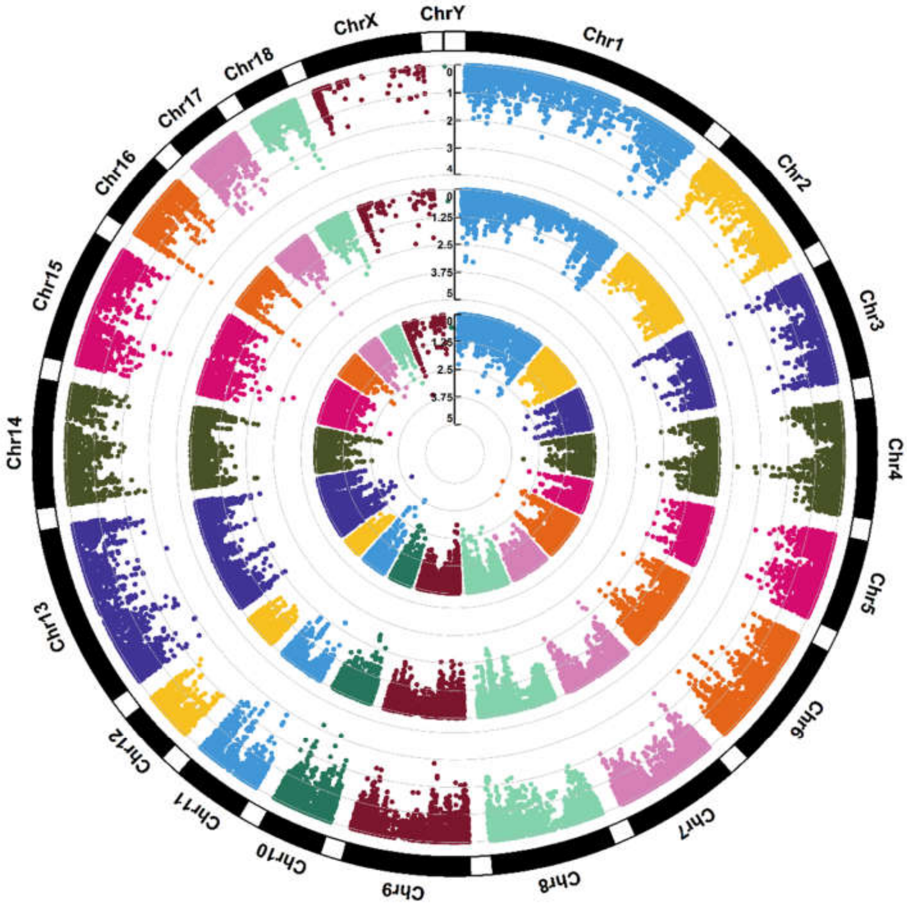

Supplementary Figure S1.11. Manhattan plots of genome-wide association for benzoic acid (M11). Note: Y-axis indicates the $\log_{10}$(p-value). Blue dotted and red solid lines indicate the genome-wide threshold of 0.05 and 0.01 after Bonferroni multiple testing, respectively. The three tracks indicate the metabolites from first sampling time, second sampling time, and combined two sampling times from outside to inside.

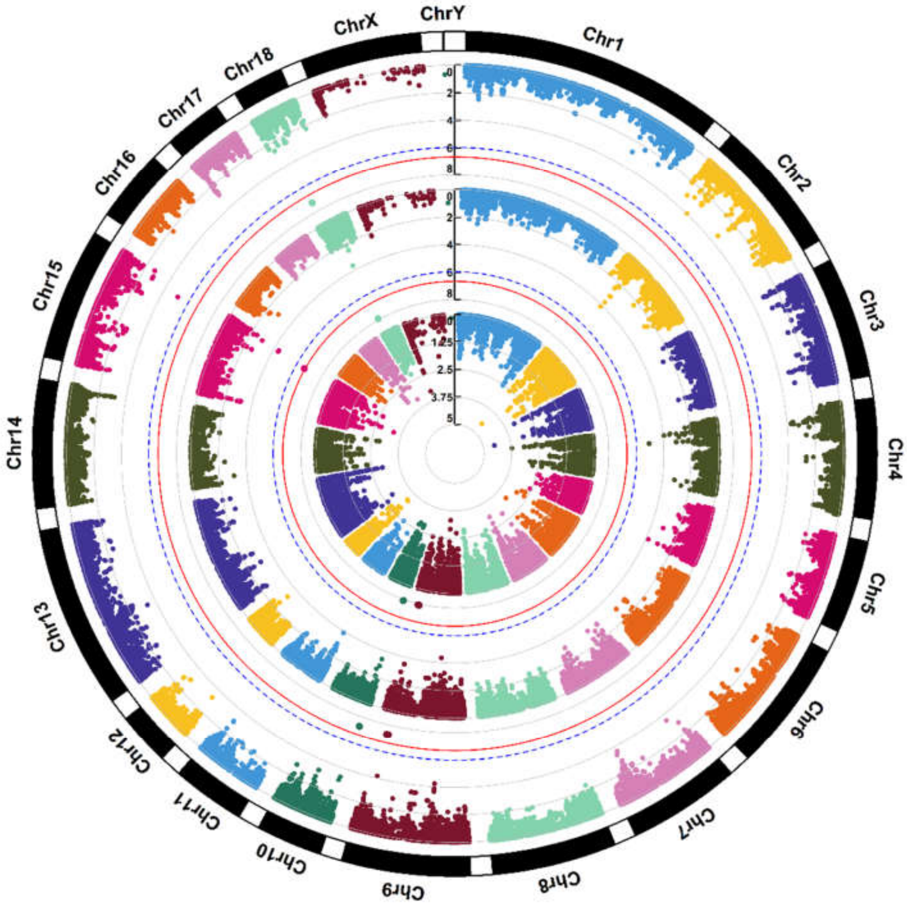

Supplementary Figure S1.12. Manhattan plots of genome-wide association for carnitine (M12). Note: Y-axis indicates the $\log_{10}(p\text{-value})$. Blue dotted and red solid lines indicate the genome-wide threshold of 0.05 and 0.01 after Bonferroni multiple testing, respectively. The three tracks indicate the metabolites from first sampling time, second sampling time, and combined two sampling times from outside to inside.

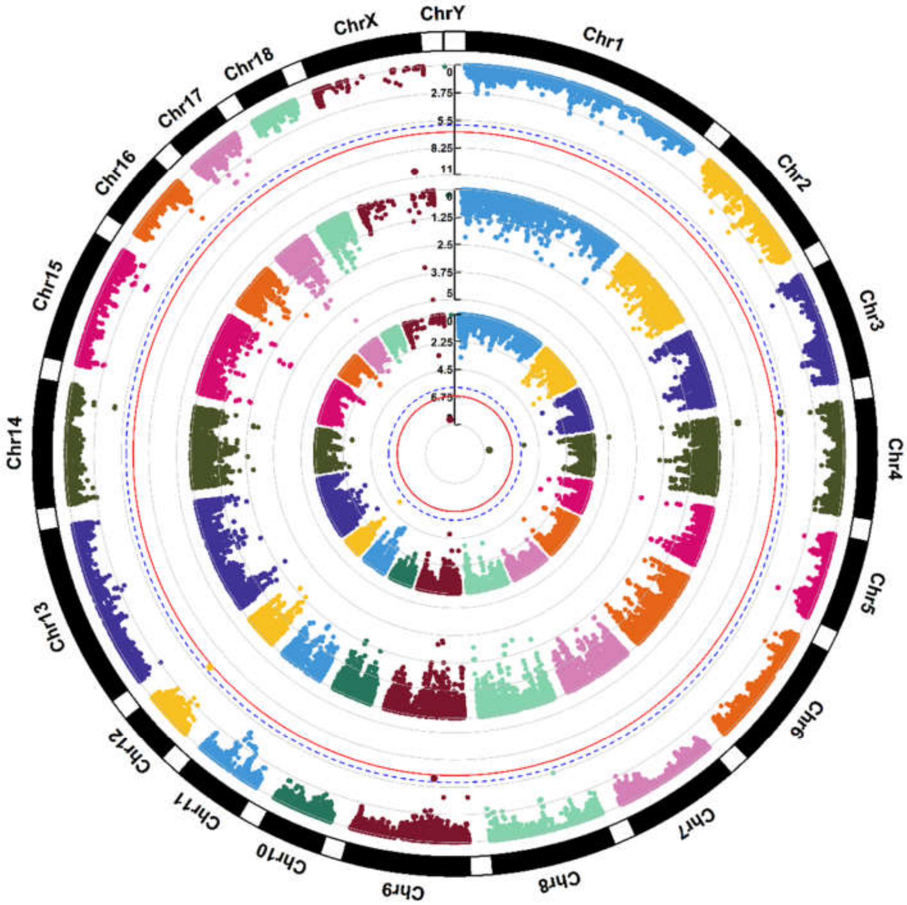

Supplementary Figure S1.13. Manhattan plots of genome-wide association for citrulline (M13). Note: Y-axis indicates the $\log_{10}$(*p*-value). Blue dotted and red solid lines indicate the genome-wide threshold of 0.05 and 0.01 after Bonferroni multiple testing, respectively. The three tracks indicate the metabolites from first sampling time, second sampling time, and combined two sampling times from outside to inside.

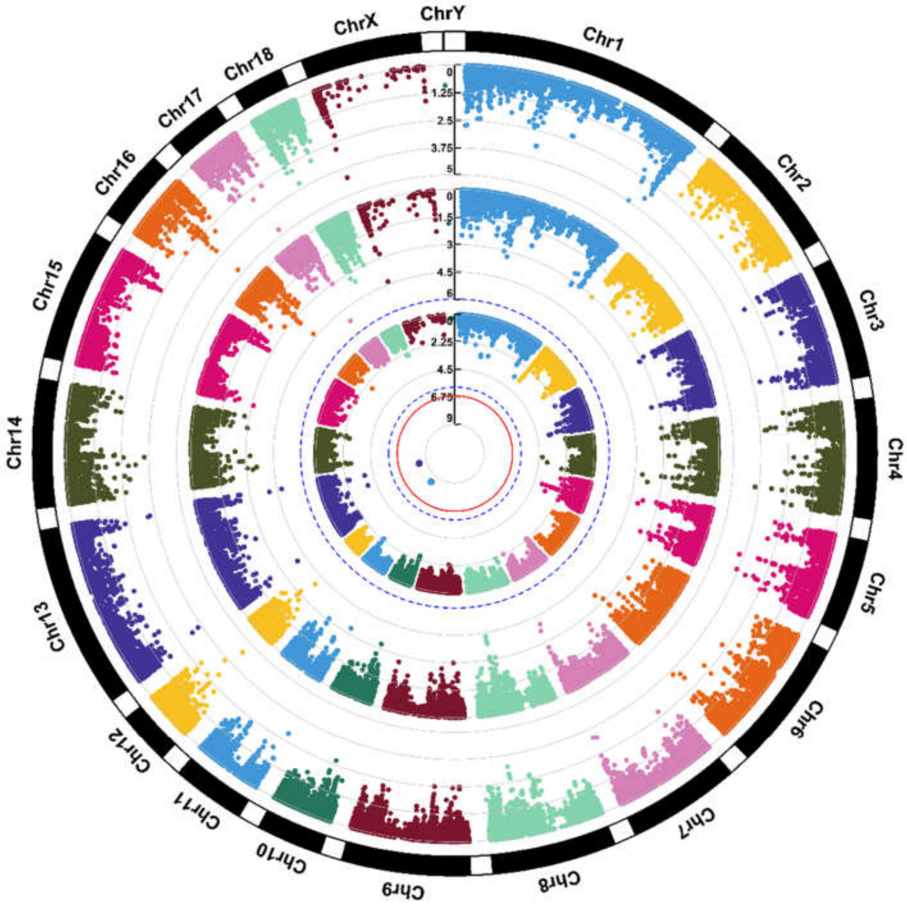

Supplementary Figure S1.14. Manhattan plots of genome-wide association for cotinine (M14). Note: Y-axis indicates the $\log_{10}$(p-value). Blue dotted and red solid lines indicate the genome-wide threshold of 0.05 and 0.01 after Bonferroni multiple testing, respectively. The three tracks indicate the metabolites from first sampling time, second sampling time, and combined two sampling times from outside to inside.

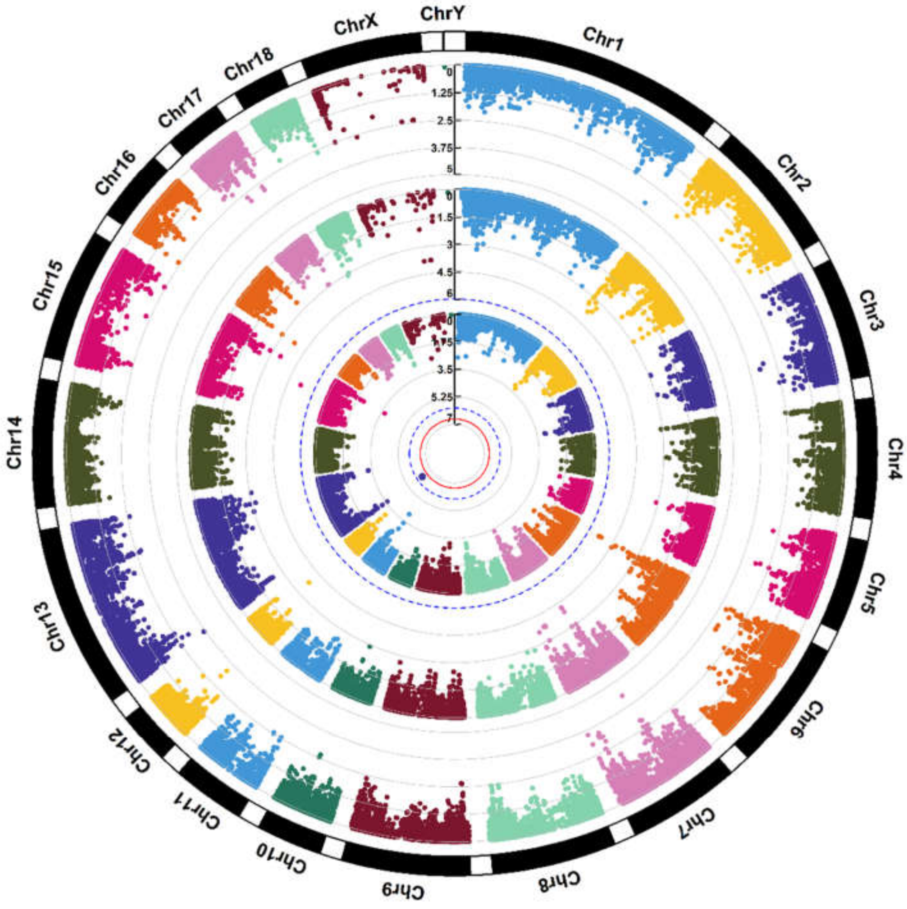

Supplementary Figure S1.15. Manhattan plots of genome-wide association for creatinine (M15). Note: Y-axis indicates the $\log_{10}$(*p*-value). Blue dotted and red solid lines indicate the genome-wide threshold of 0.05 and 0.01 after Bonferroni multiple testing, respectively. The three tracks indicate the metabolites from first sampling time, second sampling time, and combined two sampling times from outside to inside.

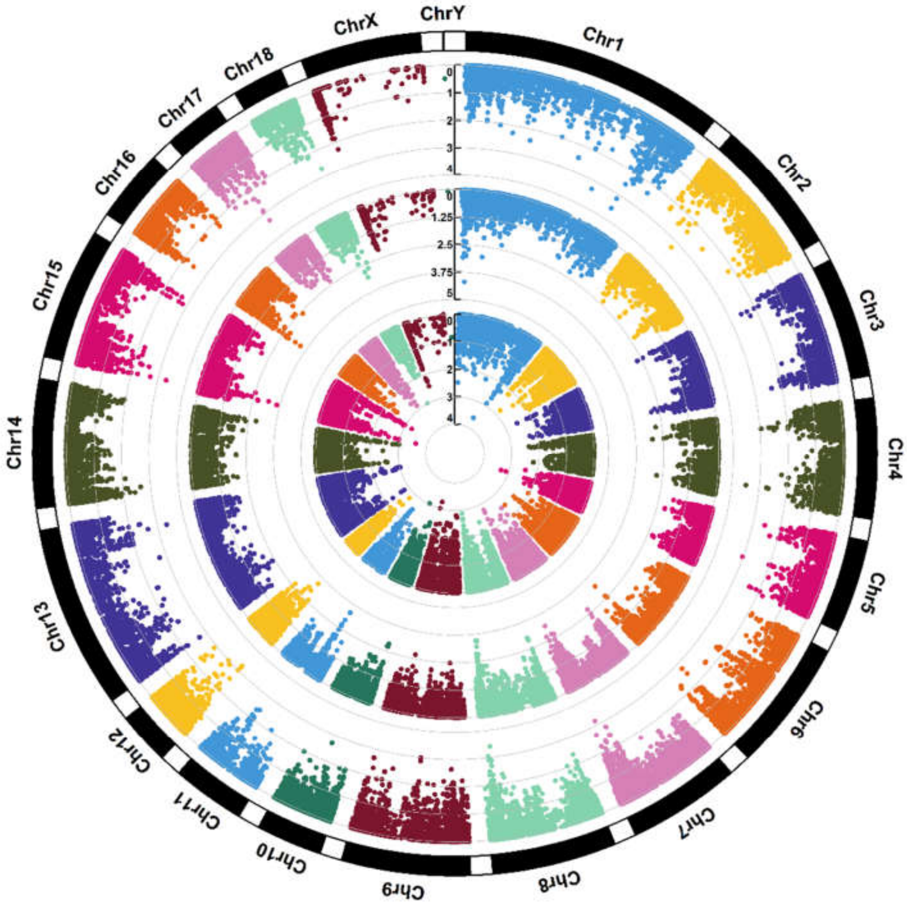

Supplementary Figure S1.16. Manhattan plots of genome-wide association for cytidine (M16). Note: Y-axis indicates the $\log_{10}$(*p*-value). Blue dotted and red solid lines indicate the genome-wide threshold of 0.05 and 0.01 after Bonferroni multiple testing, respectively. The three tracks indicate the metabolites from first sampling time, second sampling time, and combined two sampling times from outside to inside.

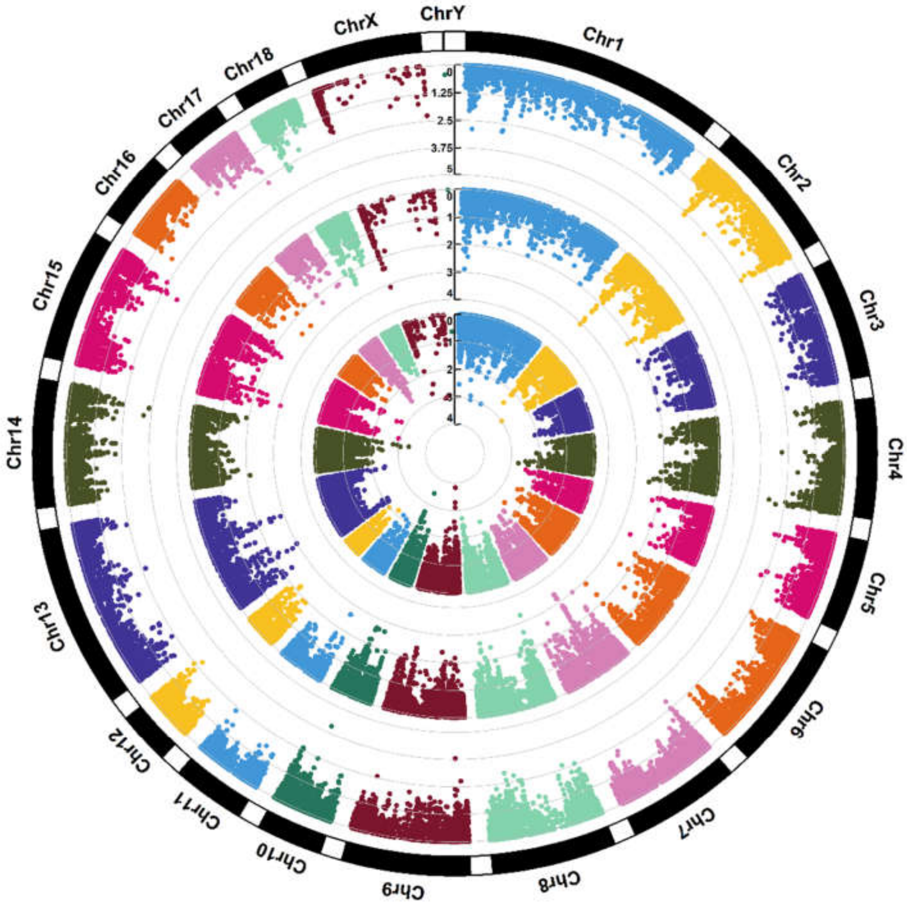

Supplementary Figure S1.17. Manhattan plots of genome-wide association for disaccharide (M17). Note: Y-axis indicates the $\log_{10}$(*p*-value). Blue dotted and red solid lines indicate the genome-wide threshold of 0.05 and 0.01 after Bonferroni multiple testing, respectively. The three tracks indicate the metabolites from first sampling time, second sampling time, and combined two sampling times from outside to inside.

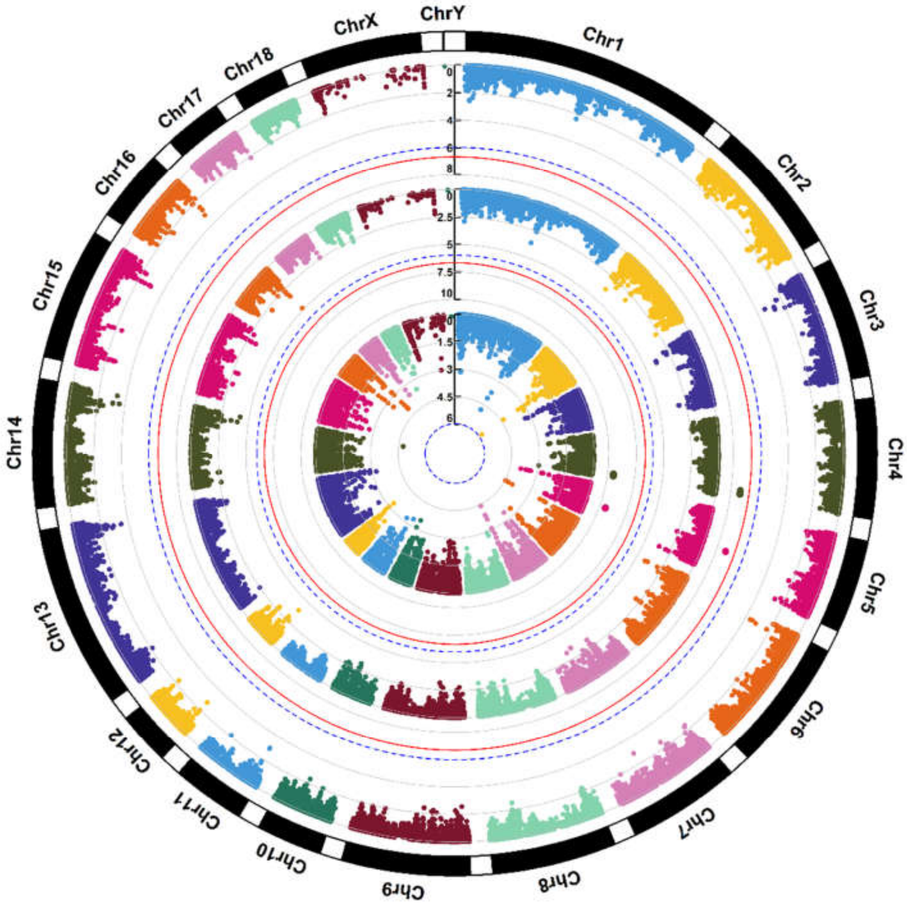

Supplementary Figure S1.18. Manhattan plots of genome-wide association for glutamic acid (M18). Note: Y-axis indicates the $\log_{10}$(p-value). Blue dotted and red solid lines indicate the genome-wide threshold of 0.05 and 0.01 after Bonferroni multiple testing, respectively. The three tracks indicate the metabolites from first sampling time, second sampling time, and combined two sampling times from outside to inside.

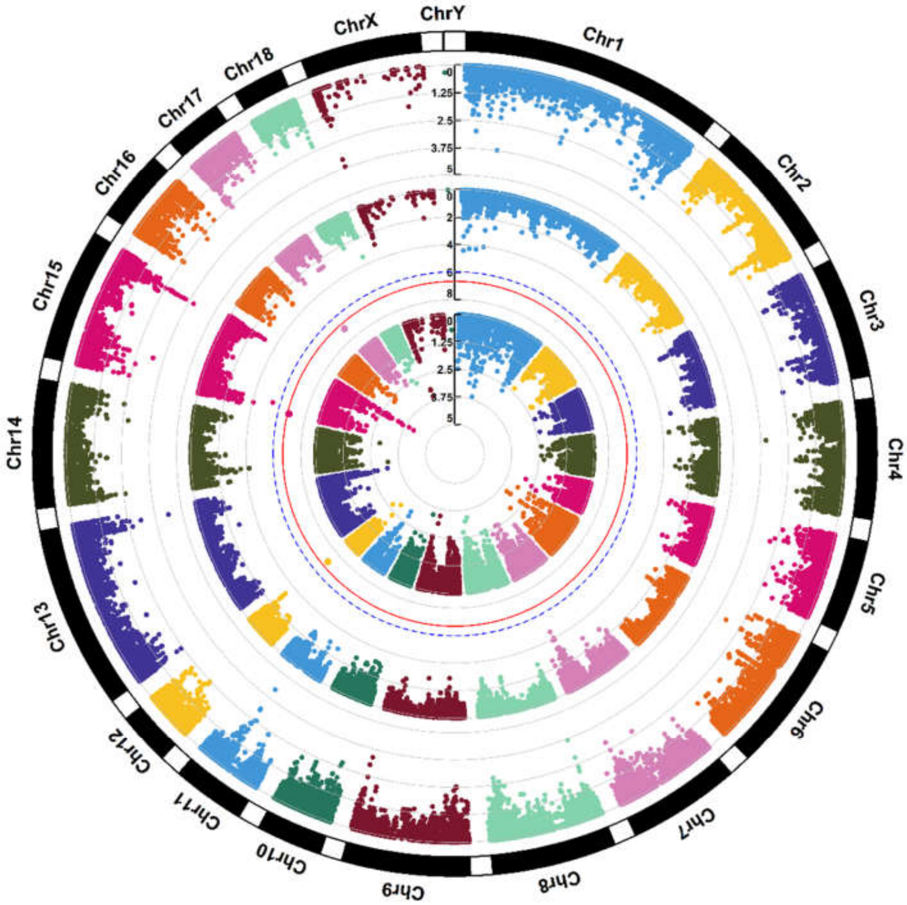

Supplementary Figure S1.19. Manhattan plots of genome-wide association for guanine (M19). Note: Y-axis indicates the $\log_{10}$(p-value). Blue dotted and red solid lines indicate the genome-wide threshold of 0.05 and 0.01 after Bonferroni multiple testing, respectively. The three tracks indicate the metabolites from first sampling time, second sampling time, and combined two sampling times from outside to inside.

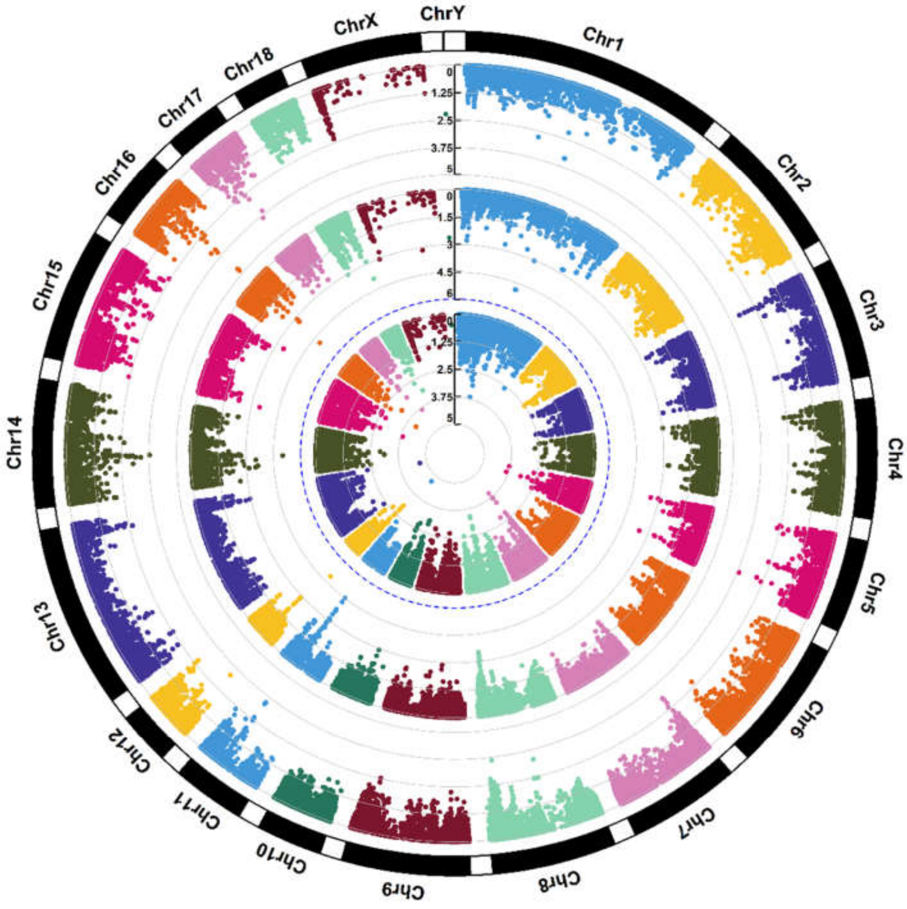

Supplementary Figure S1.20. Manhattan plots of genome-wide association for guanosine (M20). Note: Y-axis indicates the $\log_{10}$(p-value). Blue dotted and red solid lines indicate the genome-wide threshold of 0.05 and 0.01 after Bonferroni multiple testing, respectively. The three tracks indicate the metabolites from first sampling time, second sampling time, and combined two sampling times from outside to inside.

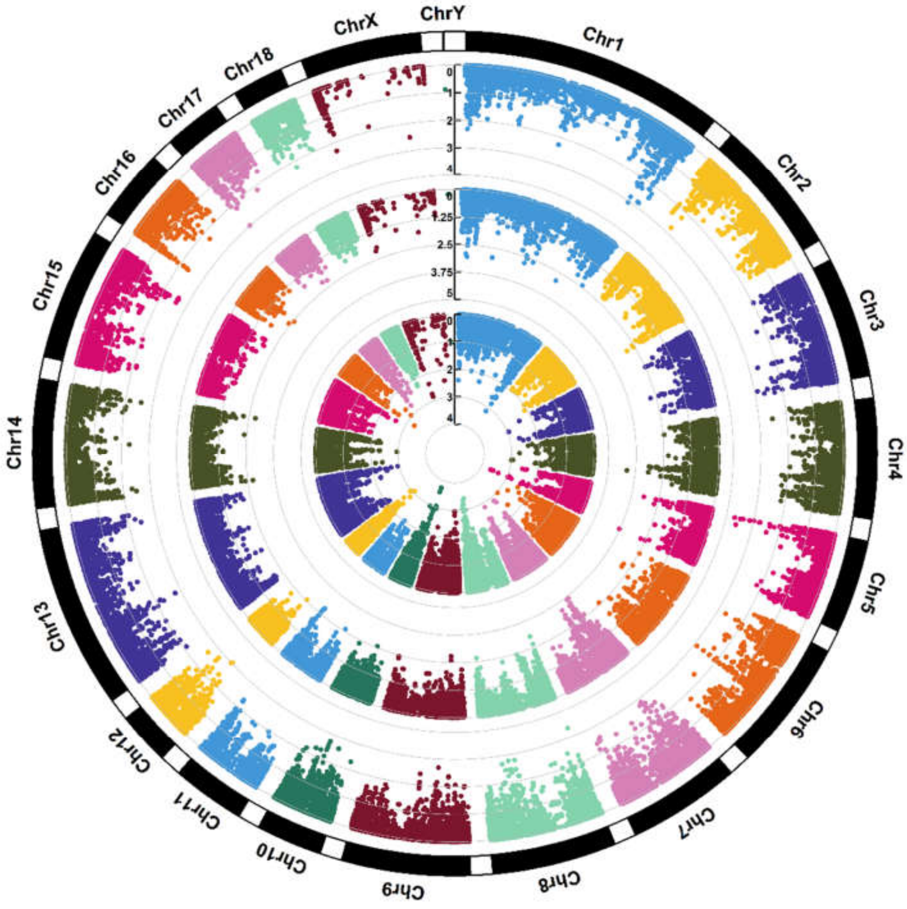

Supplementary Figure S1.21. Manhattan plots of genome-wide association for hypoxanthine (M21). Note: Y-axis indicates the $\log_{10}$(*p*-value). Blue dotted and red solid lines indicate the genome-wide threshold of 0.05 and 0.01 after Bonferroni multiple testing, respectively. The three tracks indicate the metabolites from first sampling time, second sampling time, and combined two sampling times from outside to inside.

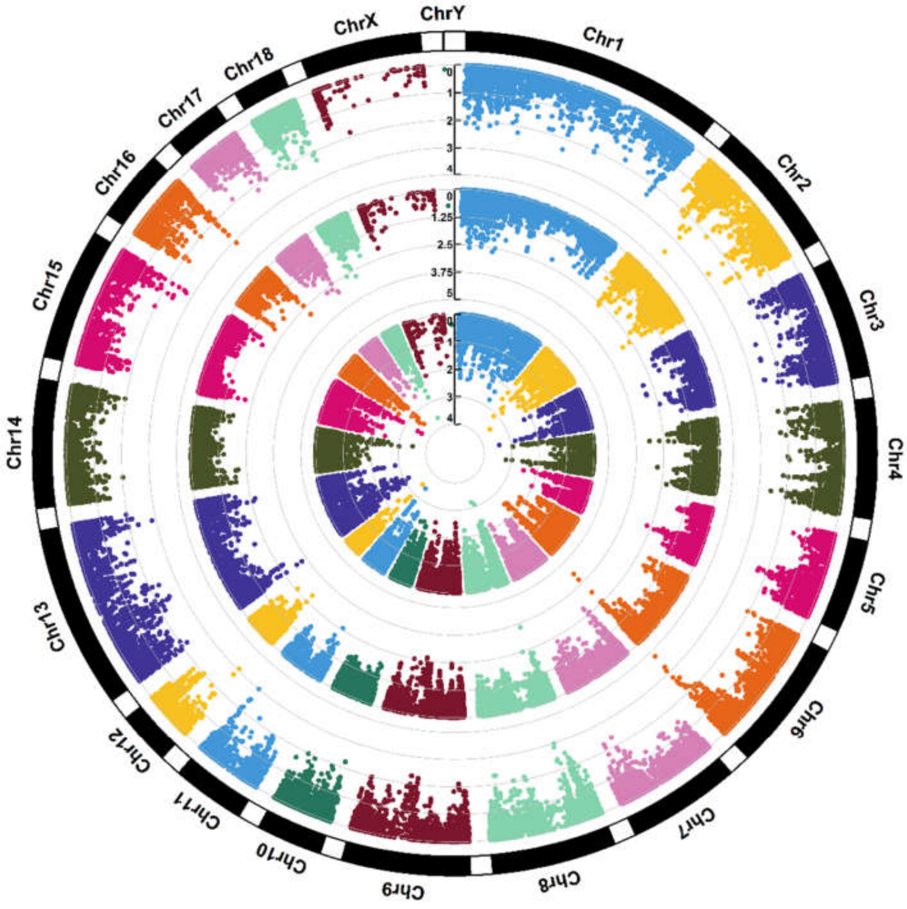

Supplementary Figure S1.22. Manhattan plots of genome-wide association for indoleacrylic acid (M22). Note: Y-axis indicates the $\log_{10}$(p-value). Blue dotted and red solid lines indicate the genome-wide threshold of 0.05 and 0.01 after Bonferroni multiple testing, respectively. The three tracks indicate the metabolites from first sampling time, second sampling time, and combined two sampling times from outside to inside.

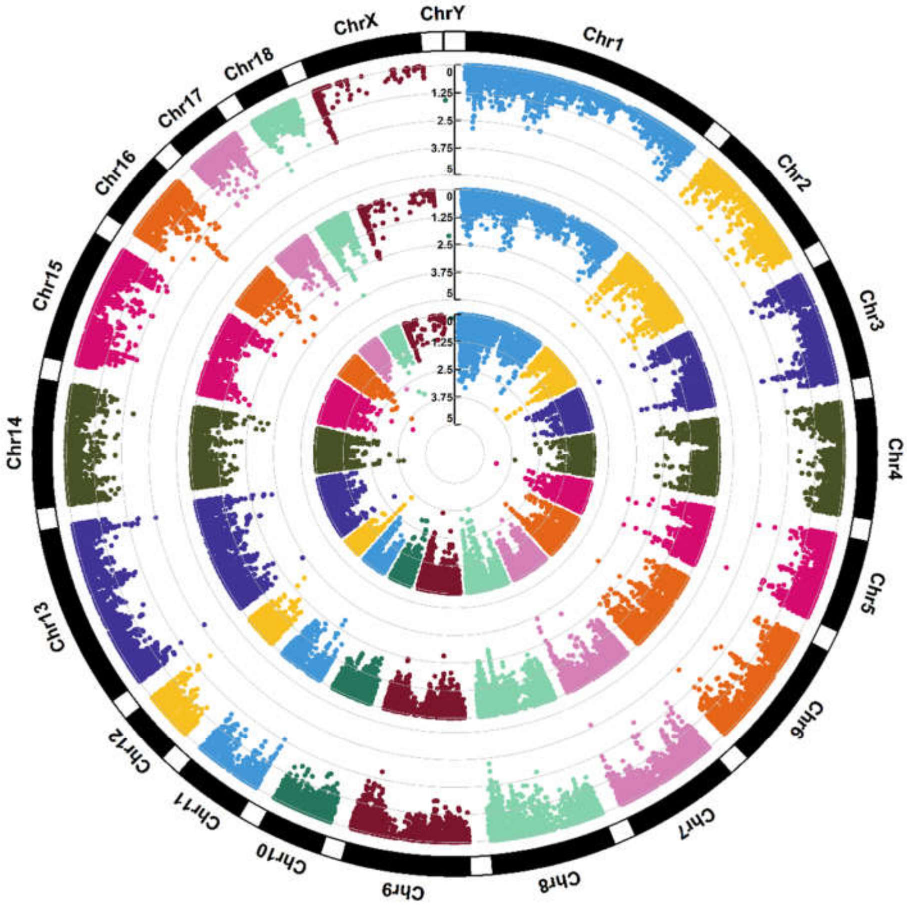

Supplementary Figure S1.23. Manhattan plots of genome-wide association for inosine (M23). Note: Y-axis indicates the $\log_{10}$(p-value). Blue dotted and red solid lines indicate the genome-wide threshold of 0.05 and 0.01 after Bonferroni multiple testing, respectively. The three tracks indicate the metabolites from first sampling time, second sampling time, and combined two sampling times from outside to inside.

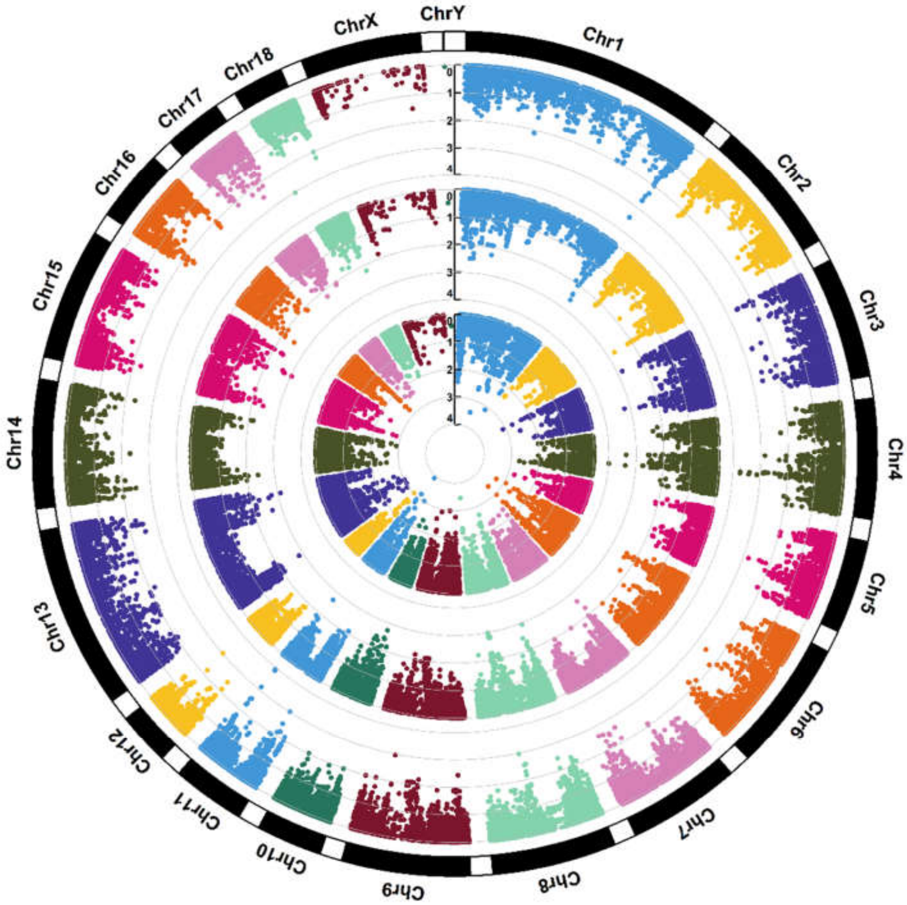

Supplementary Figure S1.24. Manhattan plots of genome-wide association for isoleucine (M24). Note: Y-axis indicates the $\log_{10}$(*p*-value). Blue dotted and red solid lines indicate the genome-wide threshold of 0.05 and 0.01 after Bonferroni multiple testing, respectively. The three tracks indicate the metabolites from first sampling time, second sampling time, and combined two sampling times from outside to inside.

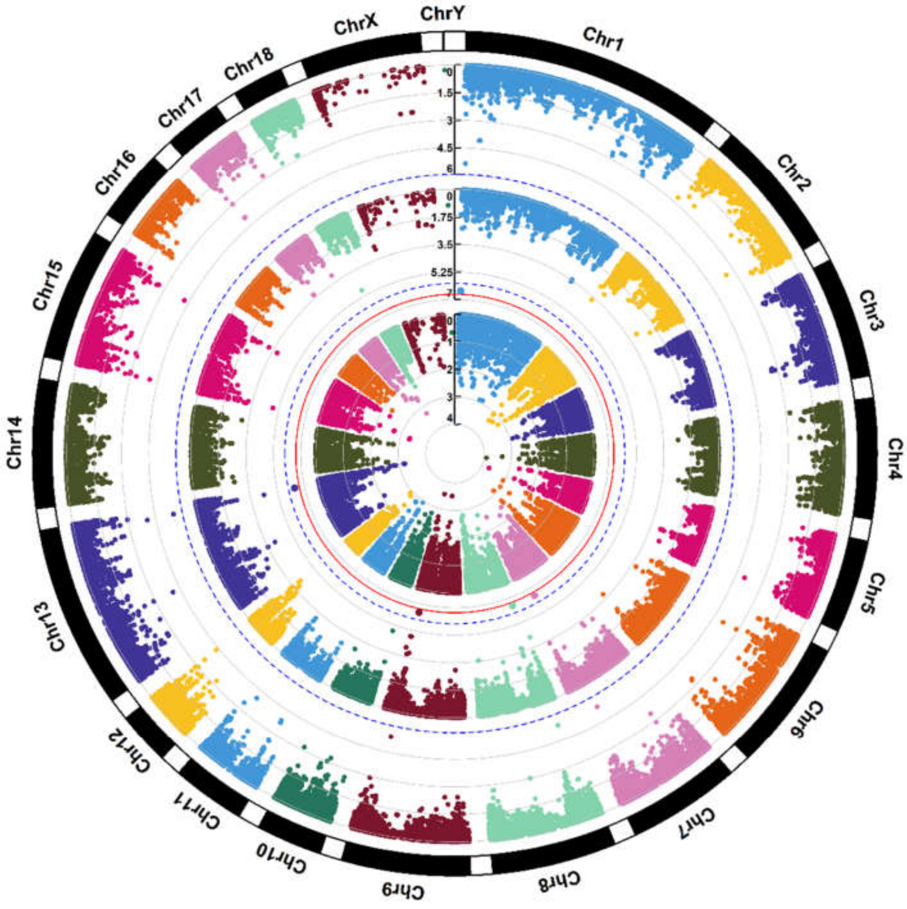

Supplementary Figure S1.25. Manhattan plots of genome-wide association for isoleucyl proline (M25). Note: Y-axis indicates the $\log_{10}$(p-value). Blue dotted and red solid lines indicate the genome-wide threshold of 0.05 and 0.01 after Bonferroni multiple testing, respectively. The three tracks indicate the metabolites from first sampling time, second sampling time, and combined two sampling times from outside to inside.

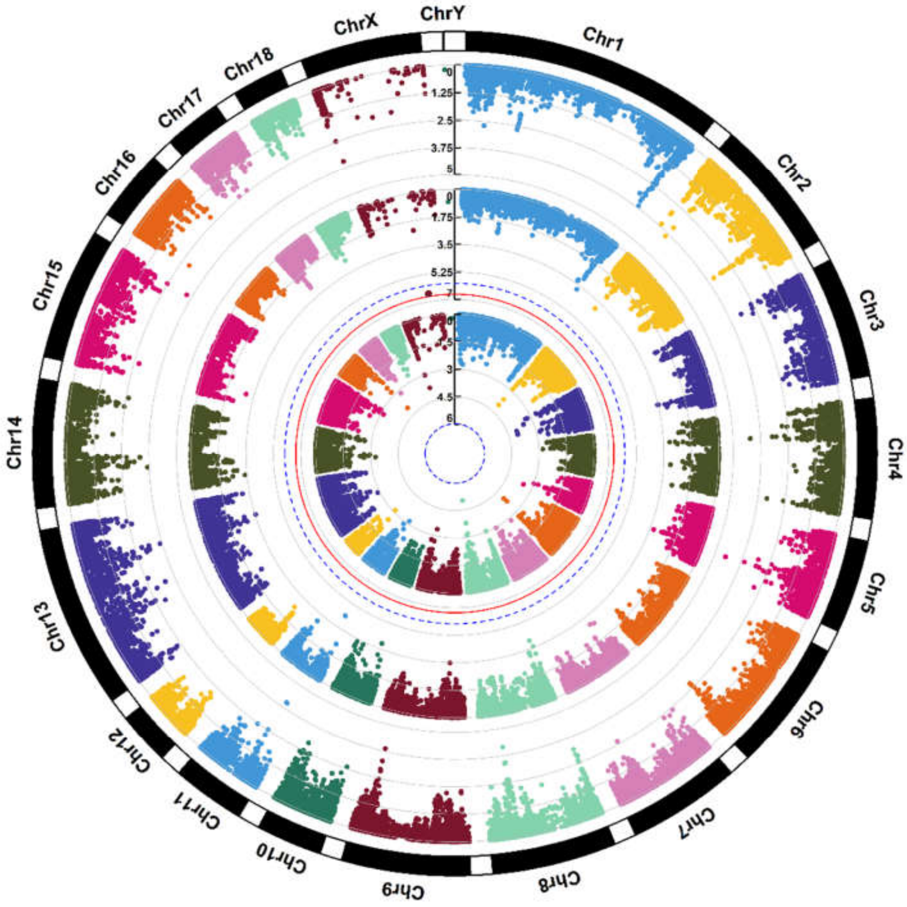

Supplementary Figure S1.26. Manhattan plots of genome-wide association for lactic acid (M27). Note: Y-axis indicates the $\log_{10}$(p-value). Blue dotted and red solid lines indicate the genome-wide threshold of 0.05 and 0.01 after Bonferroni multiple testing, respectively. The three tracks indicate the metabolites from first sampling time, second sampling time, and combined two sampling times from outside to inside.

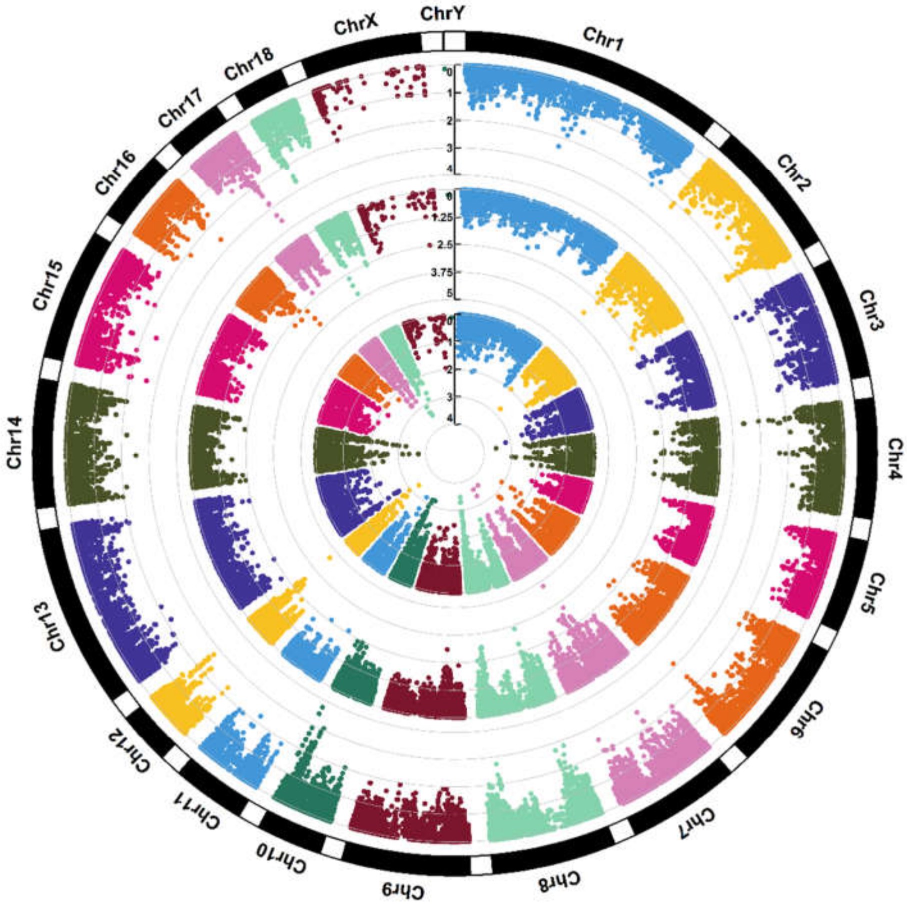

Supplementary Figure S1.27. Manhattan plots of genome-wide association for leucyl methionine (M28). Note: Y-axis indicates the log10(p-value). Blue dotted and red solid lines indicate the genome-wide threshold of 0.05 and 0.01 after Bonferroni multiple testing, respectively. The three tracks indicate the metabolites from first sampling time, second sampling time, and combined two sampling times from outside to inside.

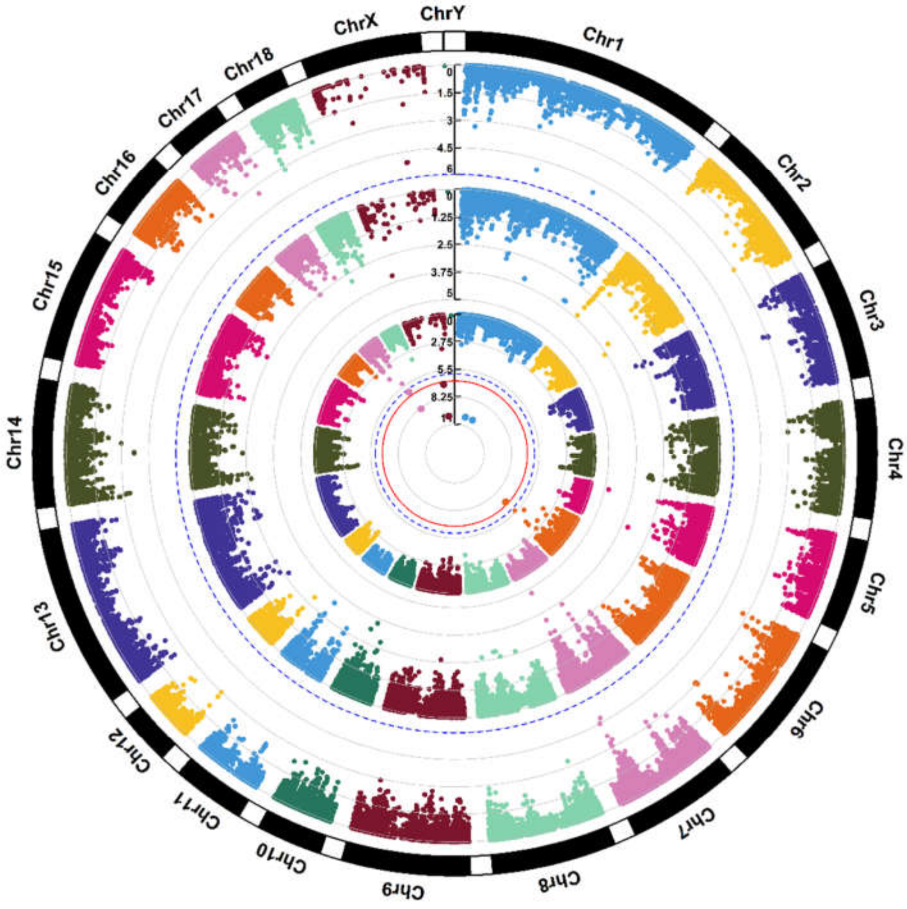

Supplementary Figure S1.28. Manhattan plots of genome-wide association for lysoPC(16:0) (M29). Note: Y-axis indicates the $\log_{10}$(*p*-value). Blue dotted and red solid lines indicate the genome-wide threshold of 0.05 and 0.01 after Bonferroni multiple testing, respectively. The three tracks indicate the metabolites from first sampling time, second sampling time, and combined two sampling times from outside to inside.

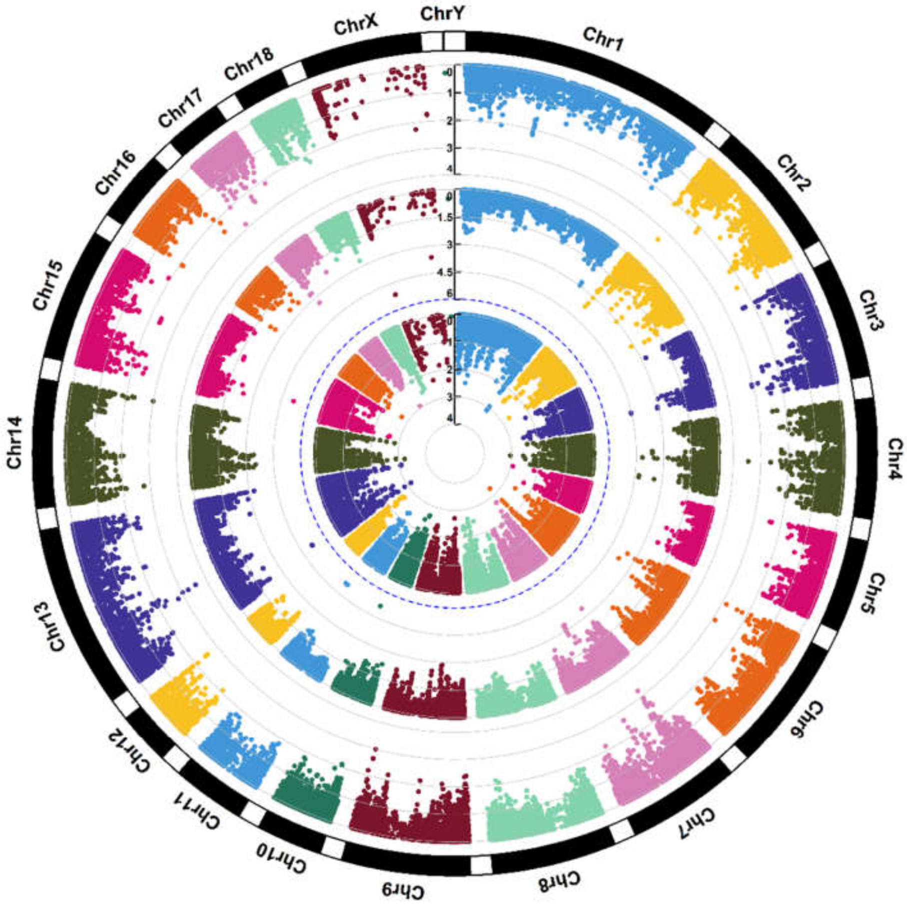

Supplementary Figure S1.29. Manhattan plots of genome-wide association for manNAc (M30). Note: Y-axis indicates the $\log_{10}$(p-value). Blue dotted and red solid lines indicate the genome-wide threshold of 0.05 and 0.01 after Bonferroni multiple testing, respectively. The three tracks indicate the metabolites from first sampling time, second sampling time, and combined two sampling times from outside to inside.

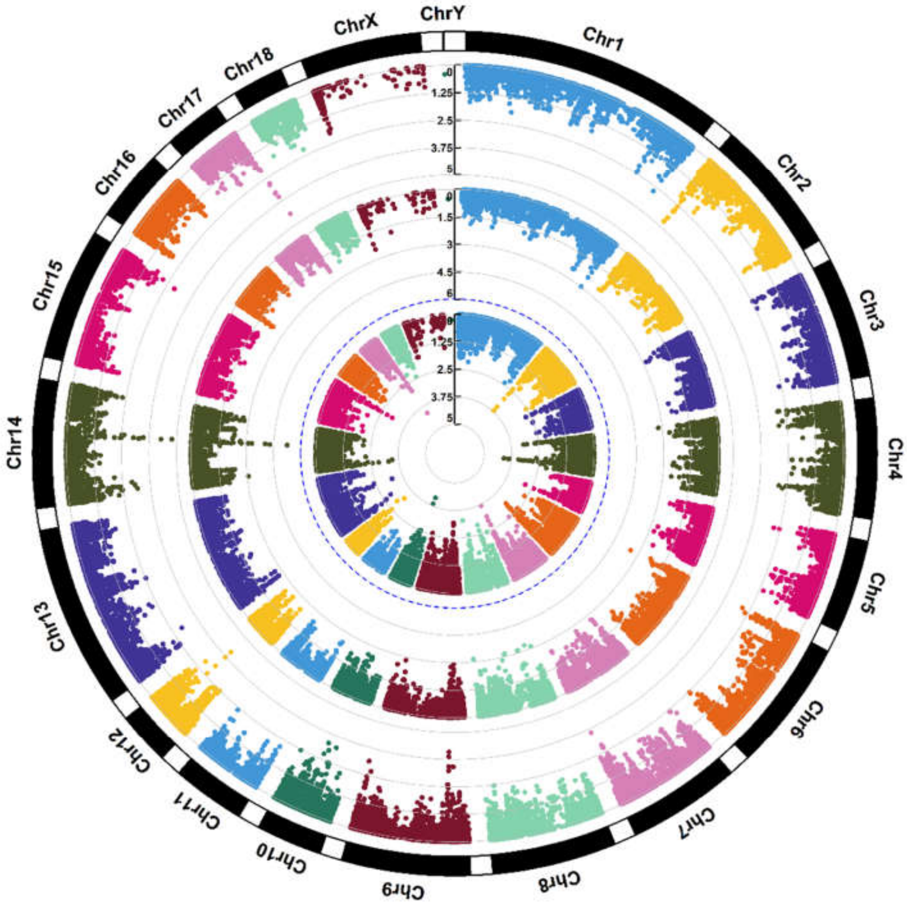

Supplementary Figure S1.30. Manhattan plots of genome-wide association for methionine (M31). Note: Y-axis indicates the $\log_{10}$(*p*-value). Blue dotted and red solid lines indicate the genome-wide threshold of 0.05 and 0.01 after Bonferroni multiple testing, respectively. The three tracks indicate the metabolites from first sampling time, second sampling time, and combined two sampling times from outside to inside.

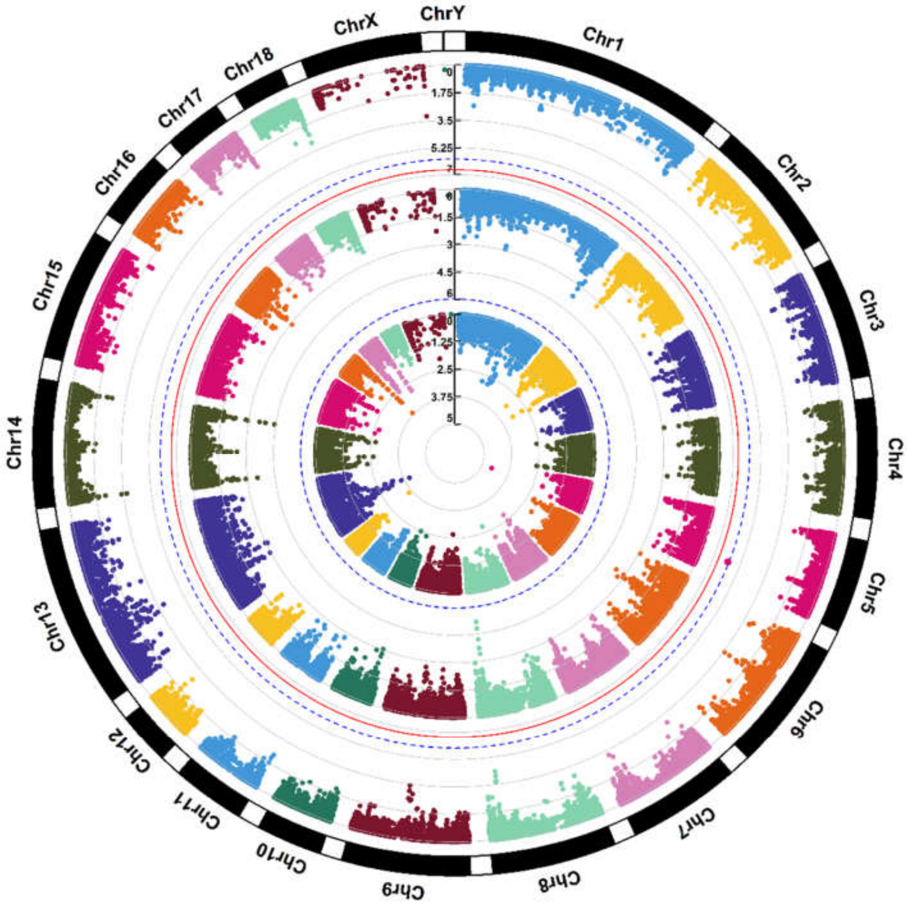

Supplementary Figure S1.31. Manhattan plots of genome-wide association for monosaccharide (M32). Note: Y-axis indicates the $\log_{10}(p\text{-value})$. Blue dotted and red solid lines indicate the genome-wide threshold of 0.05 and 0.01 after Bonferroni multiple testing, respectively. The three tracks indicate the metabolites from first sampling time, second sampling time, and combined two sampling times from outside to inside.

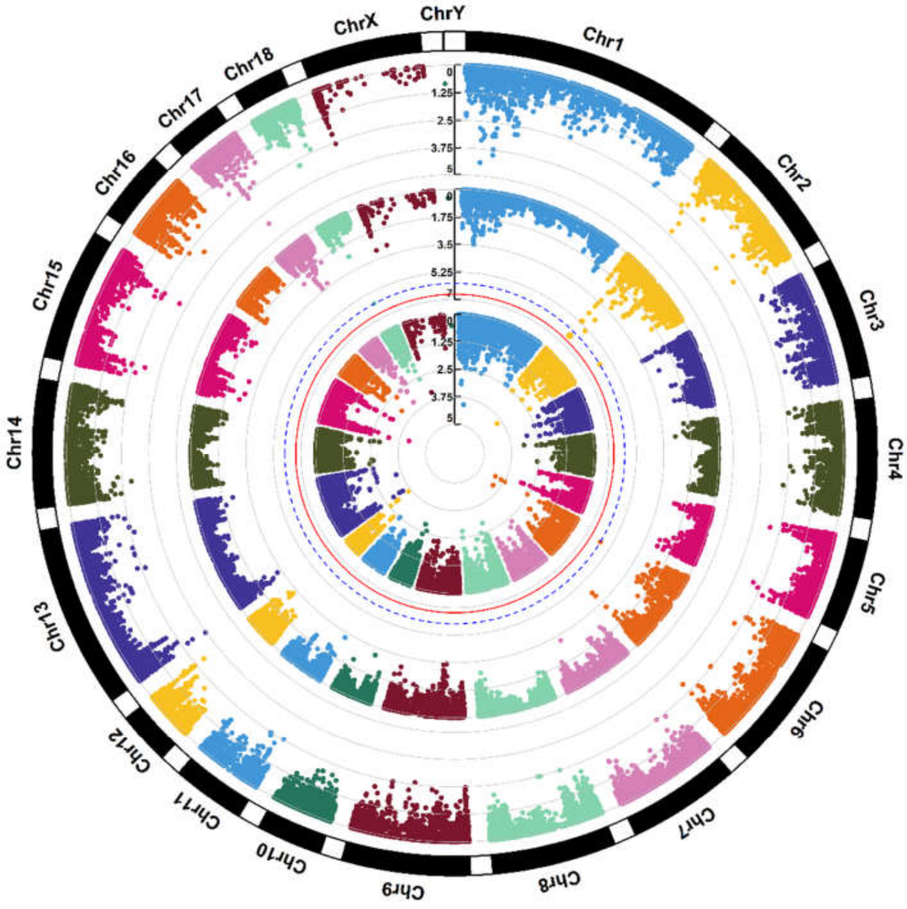

Supplementary Figure S1.32. Manhattan plots of genome-wide association for nicotine amide (M33). Note: Y-axis indicates the $\log_{10}$(p-value). Blue dotted and red solid lines indicate the genome-wide threshold of 0.05 and 0.01 after Bonferroni multiple testing, respectively. The three tracks indicate the metabolites from first sampling time, second sampling time, and combined two sampling times from outside to inside.

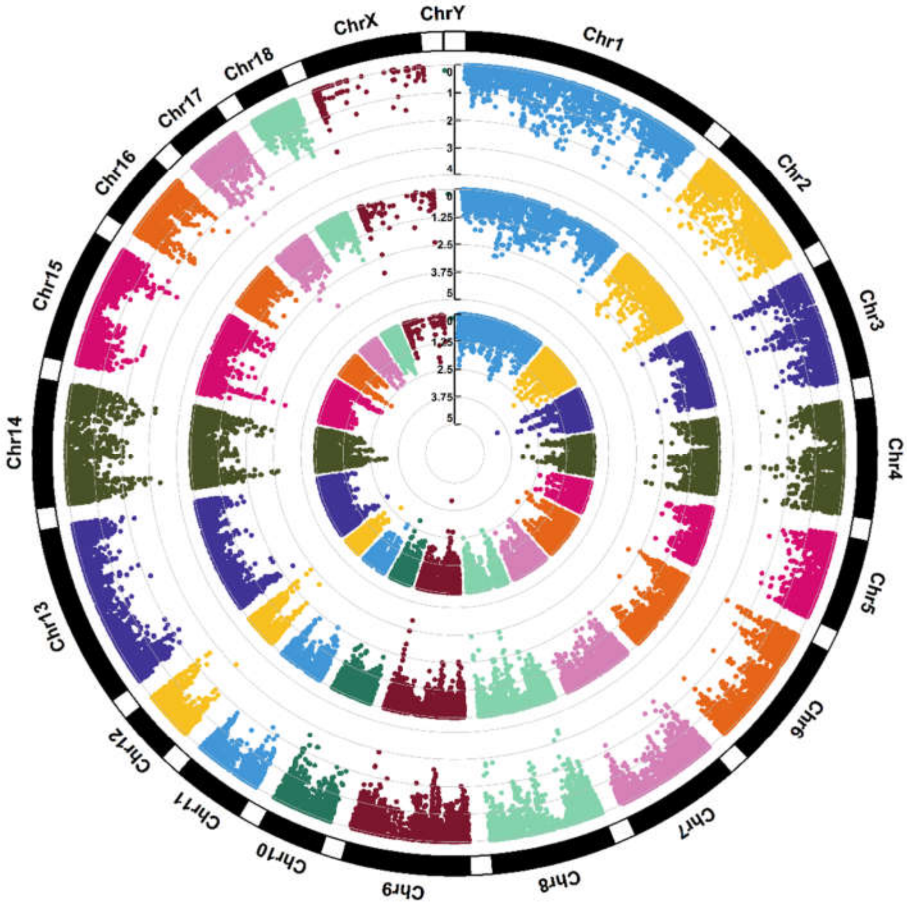

Supplementary Figure S1.33. Manhattan plots of genome-wide association for ornithine (M34). Note: Y-axis indicates the $\log_{10}$(p-value). Blue dotted and red solid lines indicate the genome-wide threshold of 0.05 and 0.01 after Bonferroni multiple testing, respectively. The three tracks indicate the metabolites from first sampling time, second sampling time, and combined two sampling times from outside to inside.

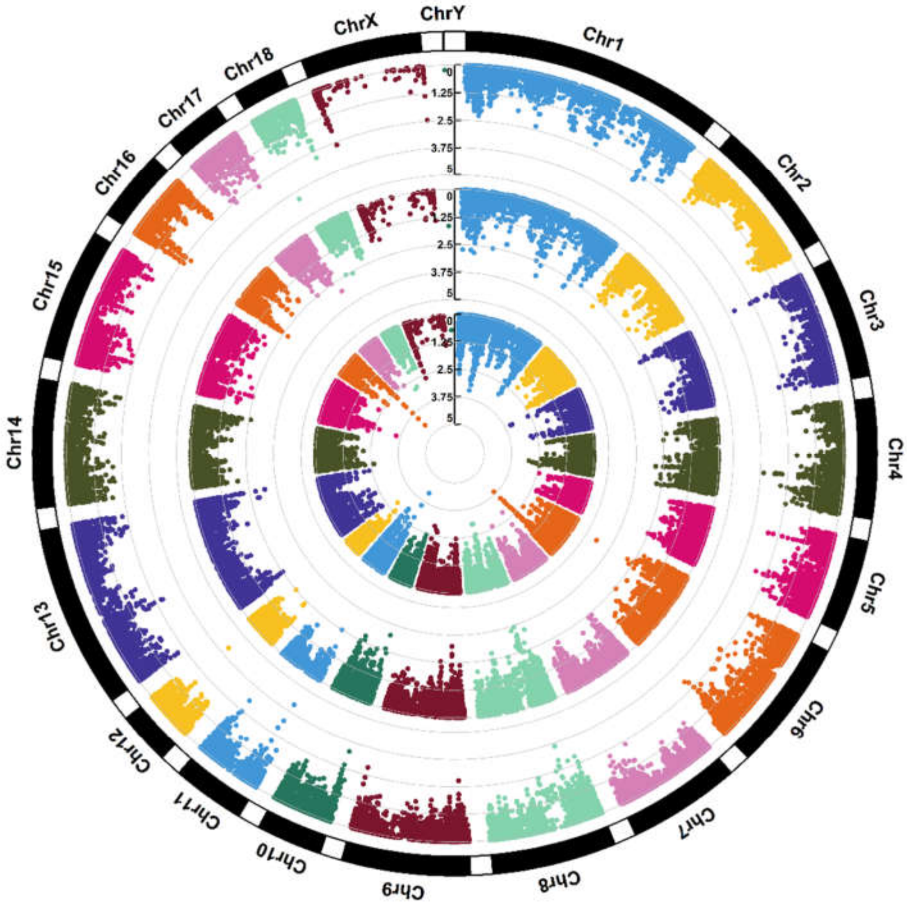

Supplementary Figure S1.34. Manhattan plots of genome-wide association for phenylalanine (M35). Note: Y-axis indicates the $\log_{10}$(p-value). Blue dotted and red solid lines indicate the genome-wide threshold of 0.05 and 0.01 after Bonferroni multiple testing, respectively. The three tracks indicate the metabolites from first sampling time, second sampling time, and combined two sampling times from outside to inside.

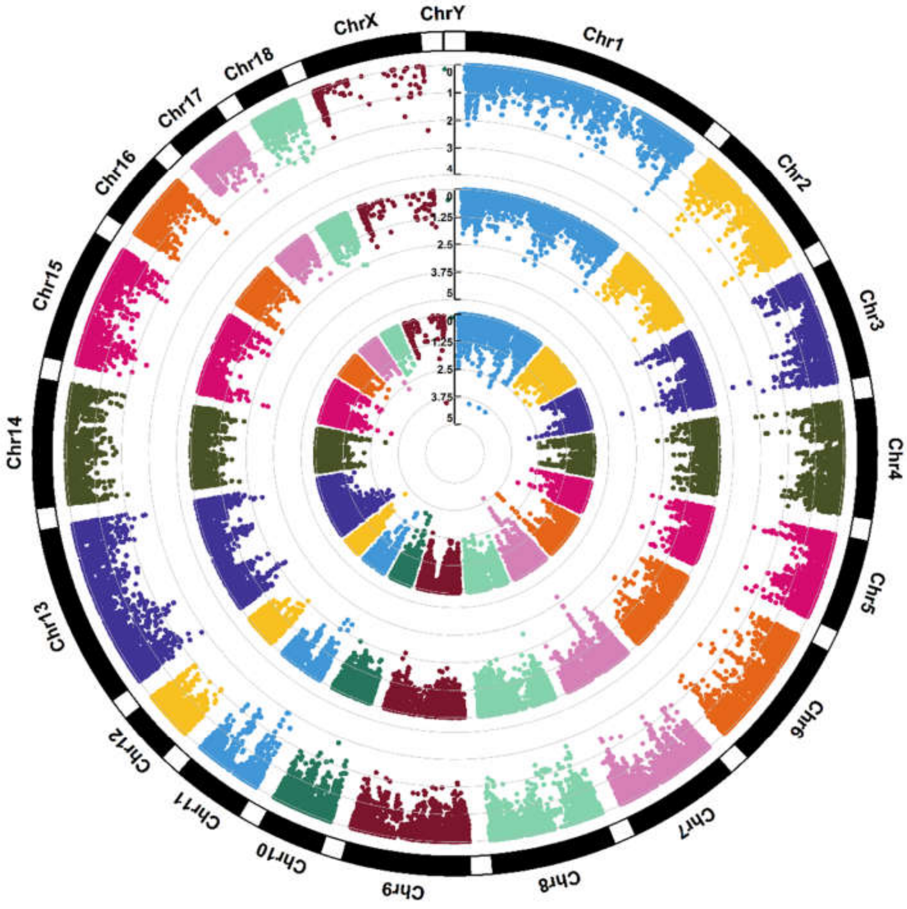

Supplementary Figure S1.35. Manhattan plots of genome-wide association for proline (M36). Note: Y-axis indicates the $\log_{10}$(*p*-value). Blue dotted and red solid lines indicate the genome-wide threshold of 0.05 and 0.01 after Bonferroni multiple testing, respectively. The three tracks indicate the metabolites from first sampling time, second sampling time, and combined two sampling times from outside to inside.

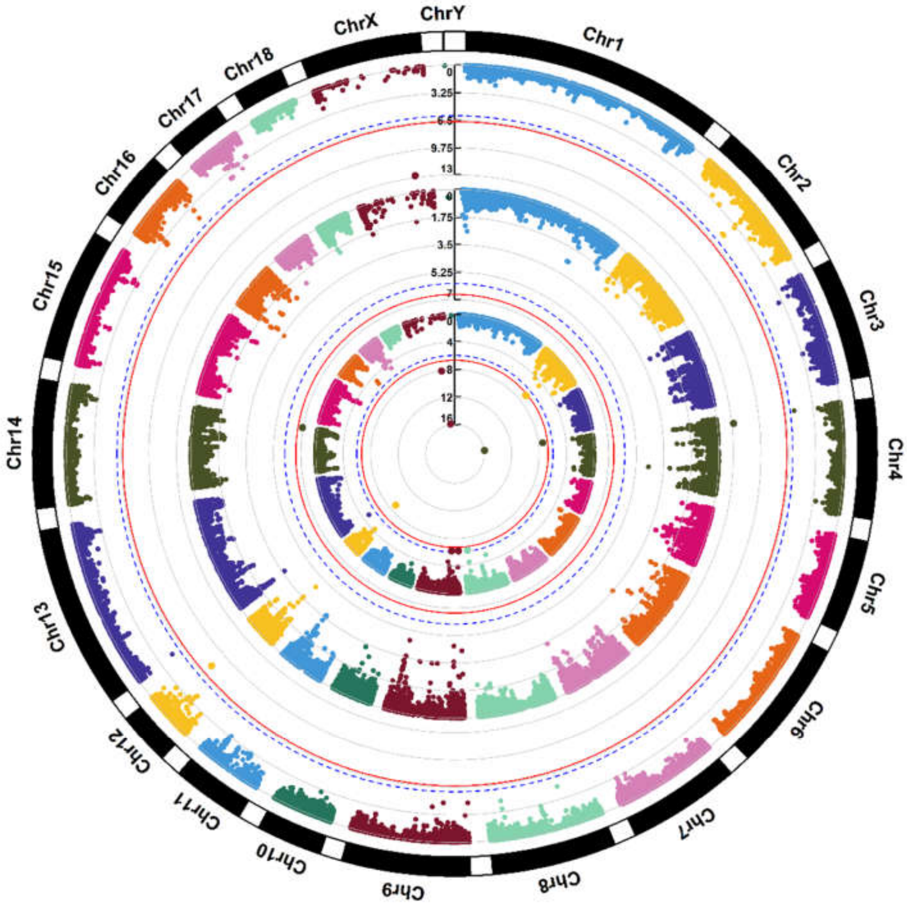

Supplementary Figure S1.36. Manhattan plots of genome-wide association for pyruvic acid (M38). Note: Y-axis indicates the log10(*p*-value). Blue dotted and red solid lines indicate the genome-wide threshold of 0.05 and 0.01 after Bonferroni multiple testing, respectively. The three tracks indicate the metabolites from first sampling time, second sampling time, and combined two sampling times from outside to inside.

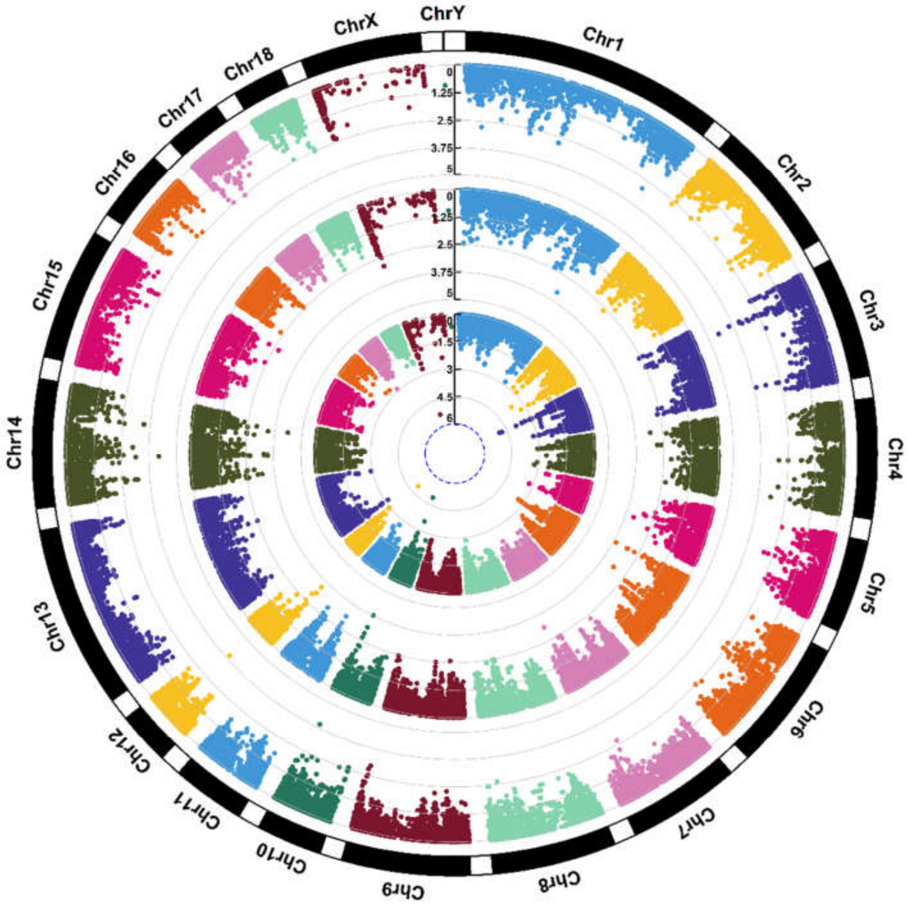

Supplementary Figure S1.37. Manhattan plots of genome-wide association for riboflavin (M39). Note: Y-axis indicates the $\log_{10}$(p-value). Blue dotted and red solid lines indicate the genome-wide threshold of 0.05 and 0.01 after Bonferroni multiple testing, respectively. The three tracks indicate the metabolites from first sampling time, second sampling time, and combined two sampling times from outside to inside.

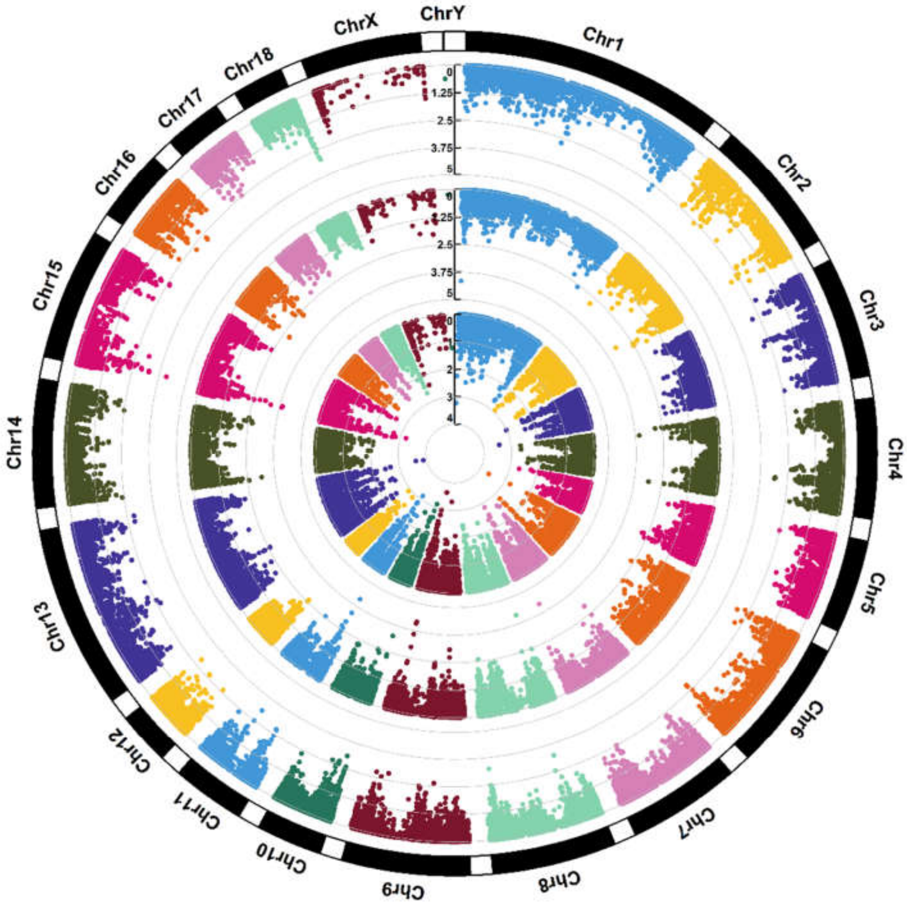

Supplementary Figure S1.38. Manhattan plots of genome-wide association for sorbitol (M40). Note: Y-axis indicates the $\log_{10}$(p-value). Blue dotted and red solid lines indicate the genome-wide threshold of 0.05 and 0.01 after Bonferroni multiple testing, respectively. The three tracks indicate the metabolites from first sampling time, second sampling time, and combined two sampling times from outside to inside.

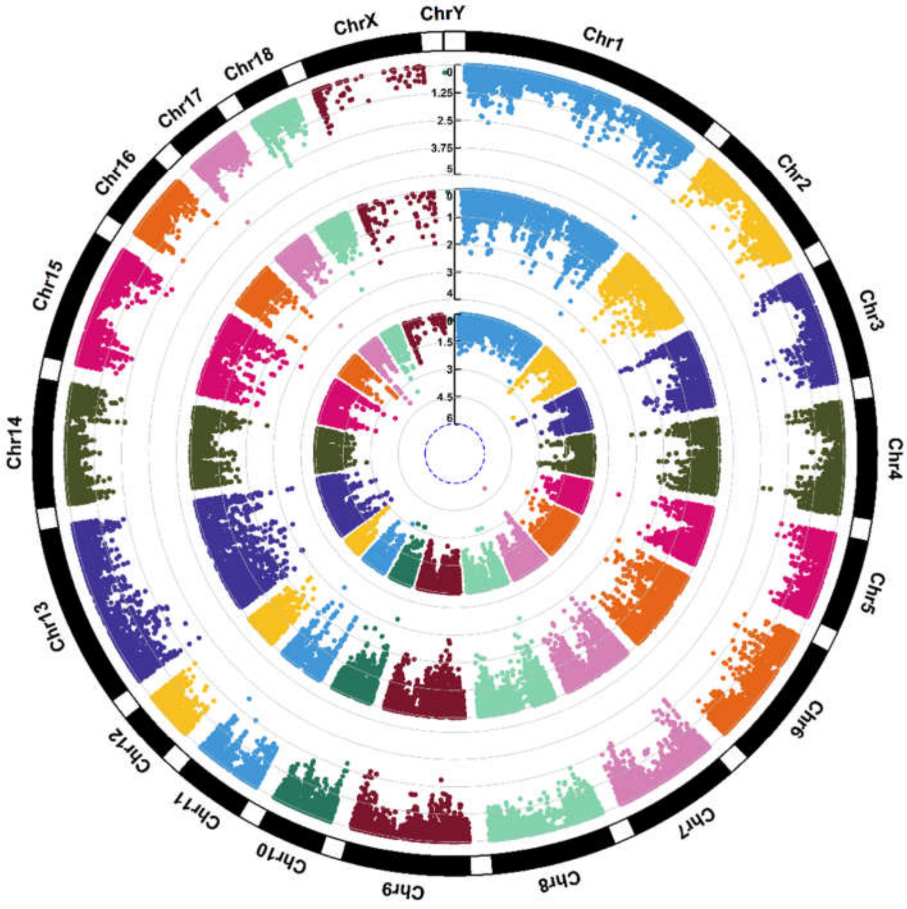

Supplementary Figure S1.39. Manhattan plots of genome-wide association for thiamine (M41). Note: Y-axis indicates the $\log_{10}$(p-value). Blue dotted and red solid lines indicate the genome-wide threshold of 0.05 and 0.01 after Bonferroni multiple testing, respectively. The three tracks indicate the metabolites from first sampling time, second sampling time, and combined two sampling times from outside to inside.

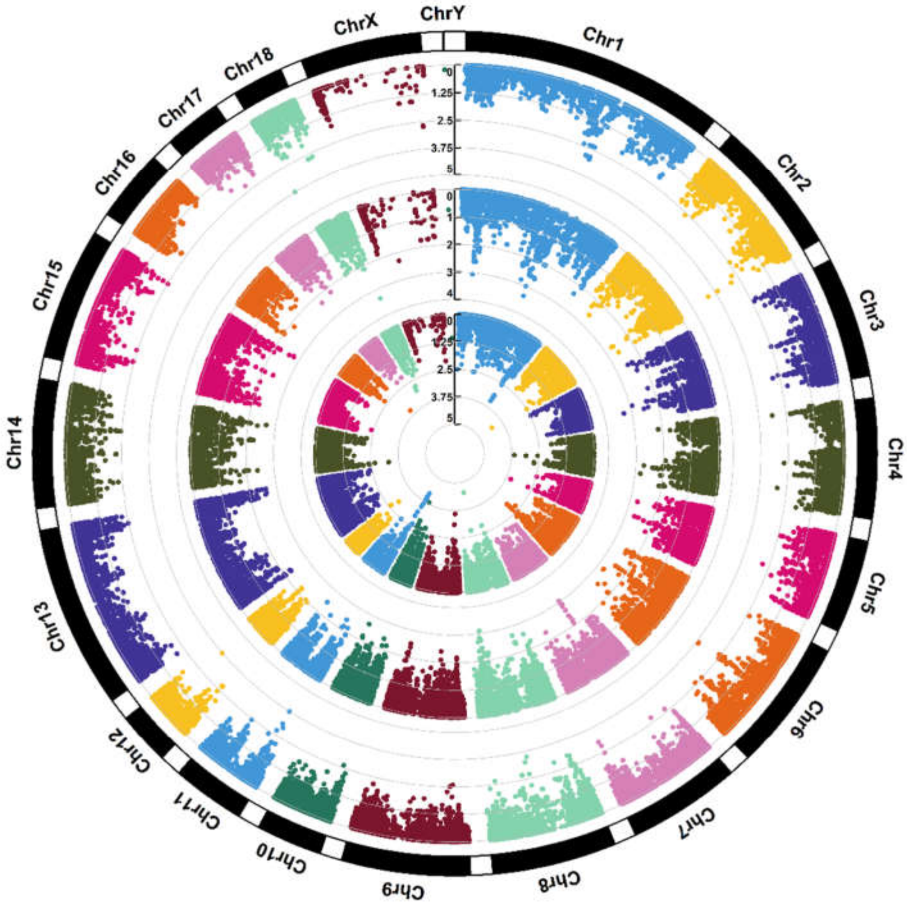

Supplementary Figure S1.40. Manhattan plots of genome-wide association for threonine (M42). Note: Y-axis indicates the $\log_{10}(p\text{-value})$. Blue dotted and red solid lines indicate the genome-wide threshold of 0.05 and 0.01 after Bonferroni multiple testing, respectively. The three tracks indicate the metabolites from first sampling time, second sampling time, and combined two sampling times from outside to inside.

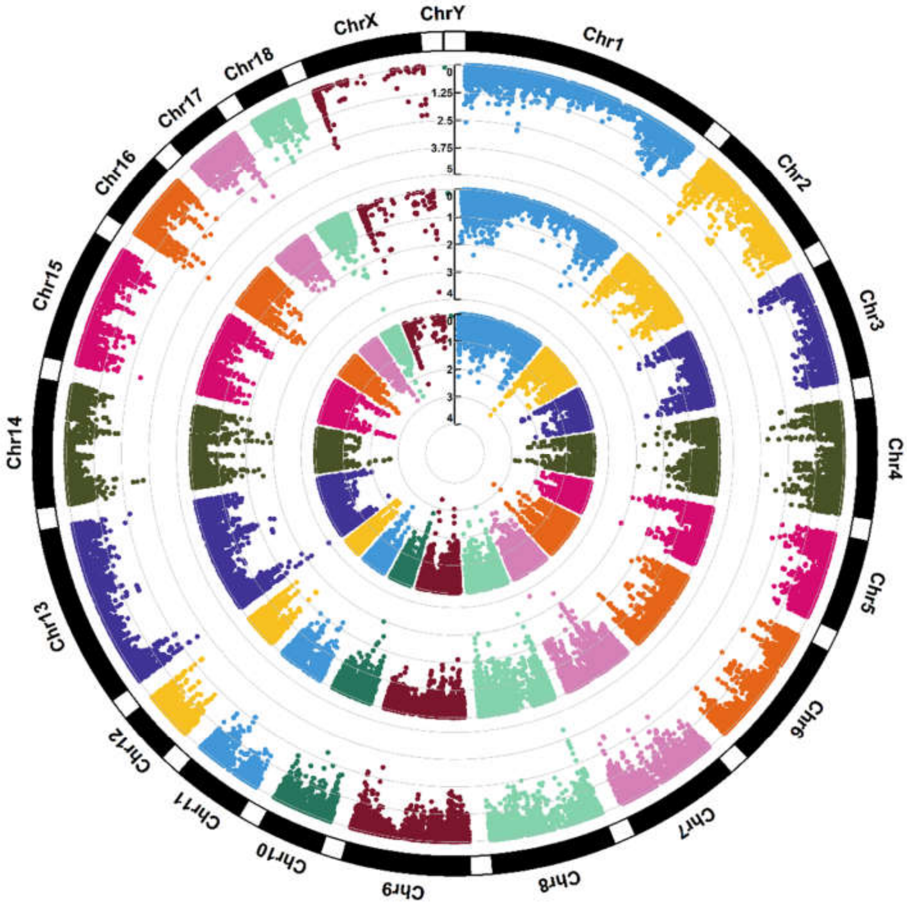

Supplementary Figure S1.41. Manhattan plots of genome-wide association for thymidine (M43). Note: Y-axis indicates the $\log_{10}$(p-value). Blue dotted and red solid lines indicate the genome-wide threshold of 0.05 and 0.01 after Bonferroni multiple testing, respectively. The three tracks indicate the metabolites from first sampling time, second sampling time, and combined two sampling times from outside to inside.

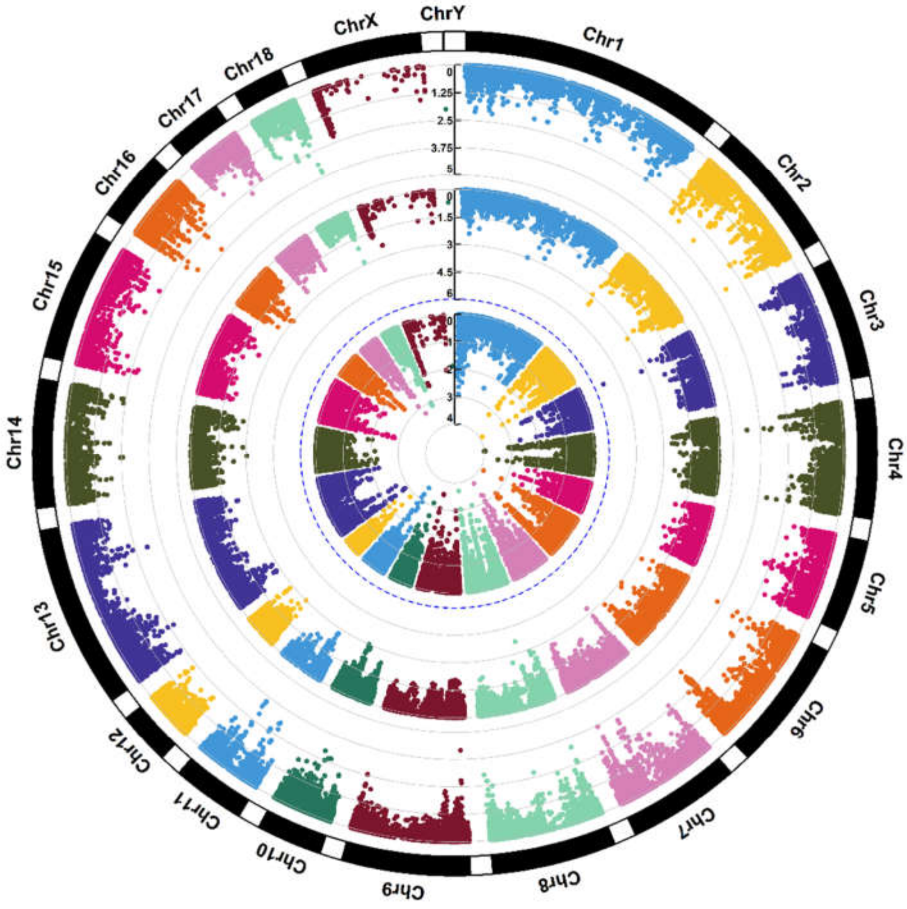

Supplementary Figure S1.42. Manhattan plots of genome-wide association for uridine (M44). Note: Y-axis indicates the $\log_{10}$(p-value). Blue dotted and red solid lines indicate the genome-wide threshold of 0.05 and 0.01 after Bonferroni multiple testing, respectively. The three tracks indicate the metabolites from first sampling time, second sampling time, and combined two sampling times from outside to inside.

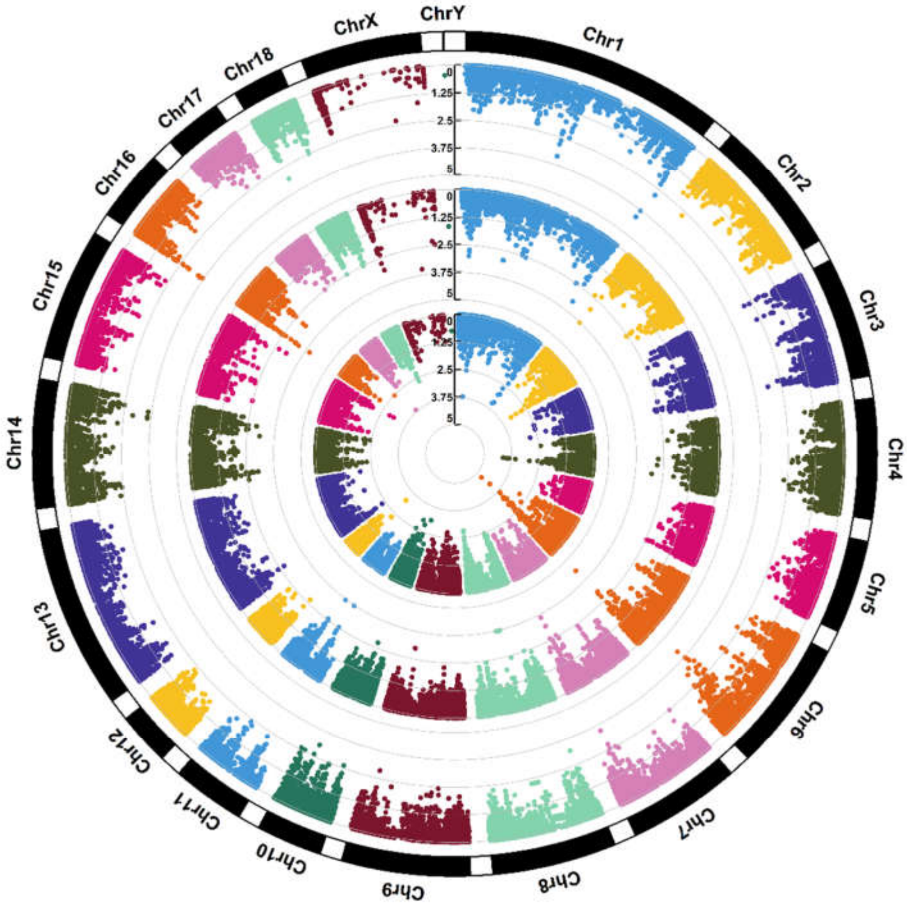

Supplementary Figure S1.43. Manhattan plots of genome-wide association for xanthine (M45). Note: Y-axis indicates the $\log_{10}$(*p*-value). Blue dotted and red solid lines indicate the genome-wide threshold of 0.05 and 0.01 after Bonferroni multiple testing, respectively. The three tracks indicate the metabolites from first sampling time, second sampling time, and combined two sampling times from outside to inside.

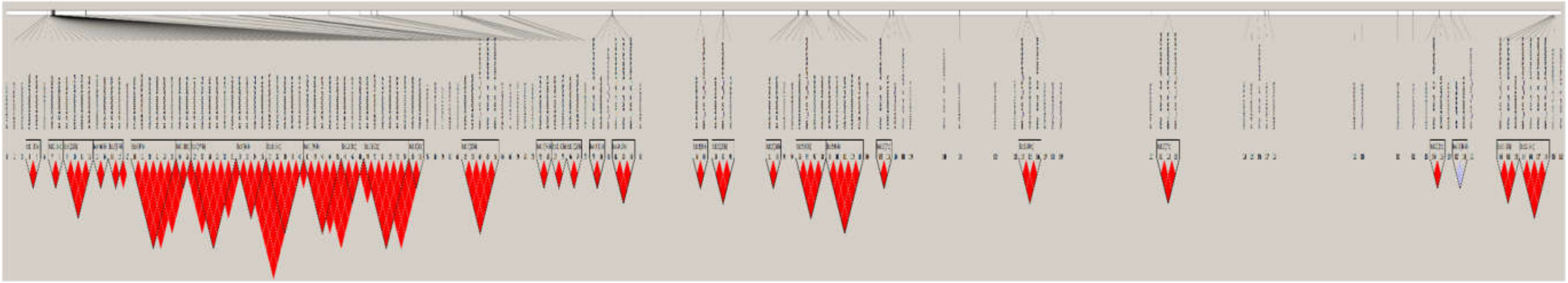

Supplementary Figure S2. Linkage disequilibrium (LD) pattern for all significant SNPs. Note: the solid line triangle indicates LD. One square indicates LD level (r^2) between two SNPs, and the squares are colored by D′ & LLOR standard scheme. D′ & LLOR standard scheme is that red indicates LLOR > 2, D′ = 1; pink indicates LLOR > 2, D′ < 1; blue indicates LLOR < 2, D′ = 1; and white indicates LLOR < 2, D′ < 1. LLOR is the logarithm of likelihood odds ratio and the reliable index to measure D′.
